# Inhibition of the hexosamine biosynthetic pathway promotes castration-resistant prostate cancer

**DOI:** 10.1038/ncomms11612

**Published:** 2016-05-19

**Authors:** Akash K. Kaushik, Ali Shojaie, Katrin Panzitt, Rajni Sonavane, Harene Venghatakrishnan, Mohan Manikkam, Alexander Zaslavsky, Vasanta Putluri, Vihas T. Vasu, Yiqing Zhang, Ayesha S. Khan, Stacy Lloyd, Adam T. Szafran, Subhamoy Dasgupta, David A. Bader, Fabio Stossi, Hangwen Li, Susmita Samanta, Xuhong Cao, Efrosini Tsouko, Shixia Huang, Daniel E. Frigo, Lawrence Chan, Dean P. Edwards, Benny A. Kaipparettu, Nicholas Mitsiades, Nancy L. Weigel, Michael Mancini, Sean E. McGuire, Rohit Mehra, Michael M. Ittmann, Arul M. Chinnaiyan, Nagireddy Putluri, Ganesh S. Palapattu, George Michailidis, Arun Sreekumar

**Affiliations:** 1 Department of Molecular and Cellular Biology and Alkek Center for Molecular Discovery, Baylor College of Medicine, Houston, Texas 77030, USA; 2 Verna and Marrs McLean Department of Biochemistry and Molecular Biology, Baylor College of Medicine, Houston, Texas 77030, USA; 3 Department of Biostatistics, University of Washington, Seattle, Washington 98195, USA; 4 Comprehensive Cancer Center, University of Michigan, Ann Arbor, Michigan 48109, USA; 5 Department of Urology, University of Michigan, Ann Arbor, Michigan 48109, USA; 6 Department of Zoology, Maharaja Sayajirao University of Baroda, Vadodara 390002, India; 7 Center for Nuclear Receptors and Cell Signaling, Department of Biology and Biochemistry, University of Houston, Houston, Texas 77204, USA; 8 Department of Pathology University of Michigan, Ann Arbor, Michigan 48109, USA; 9 Dan L Duncan Cancer Center, Baylor College of Medicine, Houston, Texas 77030, USA; 10 Genomic Medicine Program, Houston Methodist Research Institute, Houston, Texas 77030, USA; 11 Department of Molecular and Human Genetics, Baylor College of Medicine, Houston, Texas 77030, USA; 12 Department of Pathology and Immunology, Baylor College of Medicine, Houston, Texas 77030, USA; 13 Howard Hughes Medical Institute, University of Michigan, Ann Arbor, Michigan 48109, USA; 14 Department of Statistics, University of Michigan, Ann Arbor, Michigan 48109, USA

## Abstract

The precise molecular alterations driving castration-resistant prostate cancer (CRPC) are not clearly understood. Using a novel network-based integrative approach, here, we show distinct alterations in the hexosamine biosynthetic pathway (HBP) to be critical for CRPC. Expression of HBP enzyme glucosamine-phosphate N-acetyltransferase 1 (*GNPNAT1*) is found to be significantly decreased in CRPC compared with localized prostate cancer (PCa). Genetic loss-of-function of *GNPNAT1* in CRPC-like cells increases proliferation and aggressiveness, *in vitro* and *in vivo*. This is mediated by either activation of the PI3K-AKT pathway in cells expressing full-length androgen receptor (AR) or by specific protein 1 (SP1)-regulated expression of carbohydrate response element-binding protein (ChREBP) in cells containing AR-V7 variant. Strikingly, addition of the HBP metabolite UDP-N-acetylglucosamine (UDP-GlcNAc) to CRPC-like cells significantly decreases cell proliferation, both *in-vitro* and in animal studies, while also demonstrates additive efficacy when combined with enzalutamide *in-vitro*. These observations demonstrate the therapeutic value of targeting HBP in CRPC.

## Abstract (web summary)

The molecular alterations driving anti-androgen resistance in prostate cancer are unclear. Here, the authors show, using a network-based approach, that inhibition of the hexosamine biosynthetic pathway is necessary to develop resistance and that increasing the activity of the pathway enhances the anti-androgen response.

## Graphical abstract

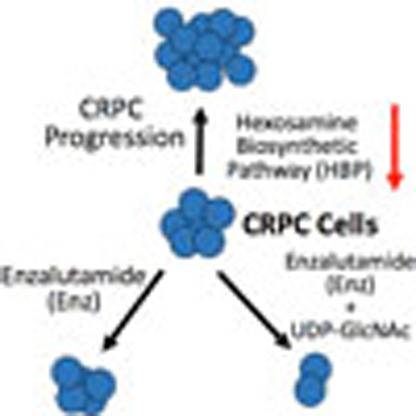

Recent scientific and technological advances have enabled biomedical researchers to collect high-throughput genomic, proteomic and metabolomic data. Functional interactions of these biomolecules have been obtained and catalogued in carefully curated databases, often in the form of networks^1^,^2^. A growing body of evidence suggests that combining information from such high-throughput data in the context of their molecular interactions may shed light on the biology of complex diseases, including cancer^3^,^4^,^5^,^6^.

The development and progression of prostate cancer (PCa) from the androgen-dependent (AD) to castration-resistant state is thought to be driven by multiple events^6^,^7^,^8^,^9^. The initial treatment of choice for advanced disease is androgen ablation, which while initially effective often leads to therapy resistance (that is, the castration resistant state). Better and more effective therapies are needed for CRPC, a lethal condition. Our previous studies^10^ identified specific metabolic alterations associated with localized PCa (treatment naive) and CRPC tumours. These findings are particularly important as they provide new insights into PCa progression and suggest metabolic targets for therapeutic intervention.

Building on this work, we employ a novel network-based integrative approach that combines patient-derived PCa gene expression with metabolomic data and identify a vital role for hexosamine biosynthetic pathway (HBP) in the castration-resistant state. Furthermore, downregulation of HBP enhances tumorigenicity of CRPC-like cells via activation of cell cycle genes regulated by either the PI3K-AKT or the SP1-ChREBP axis in cells containing either AR-full length (AR-FL) or AR-V7, respectively. Remarkably, treatment with UDP-N-acetylglucosamine (UDP-GlcNAc) significantly decreases proliferation and tumour growth of CRPC-like cells while also increases efficacy of the anti-androgen enzalutamide in *in-vitro* experiments. These findings are especially significant given that the CRPC-like cells tested, inclusive of those containing AR-V7, are inherently resistant to enzalutamide. In aggregate, our data provide novel perspectives on the influence of HBP on the castration-resistant state and supply rationale for targeting the HBP in CRPC.

## Results and Discussion

### Novel integrative analysis uncovers a role of HBP in PCa

**Figure 1:**
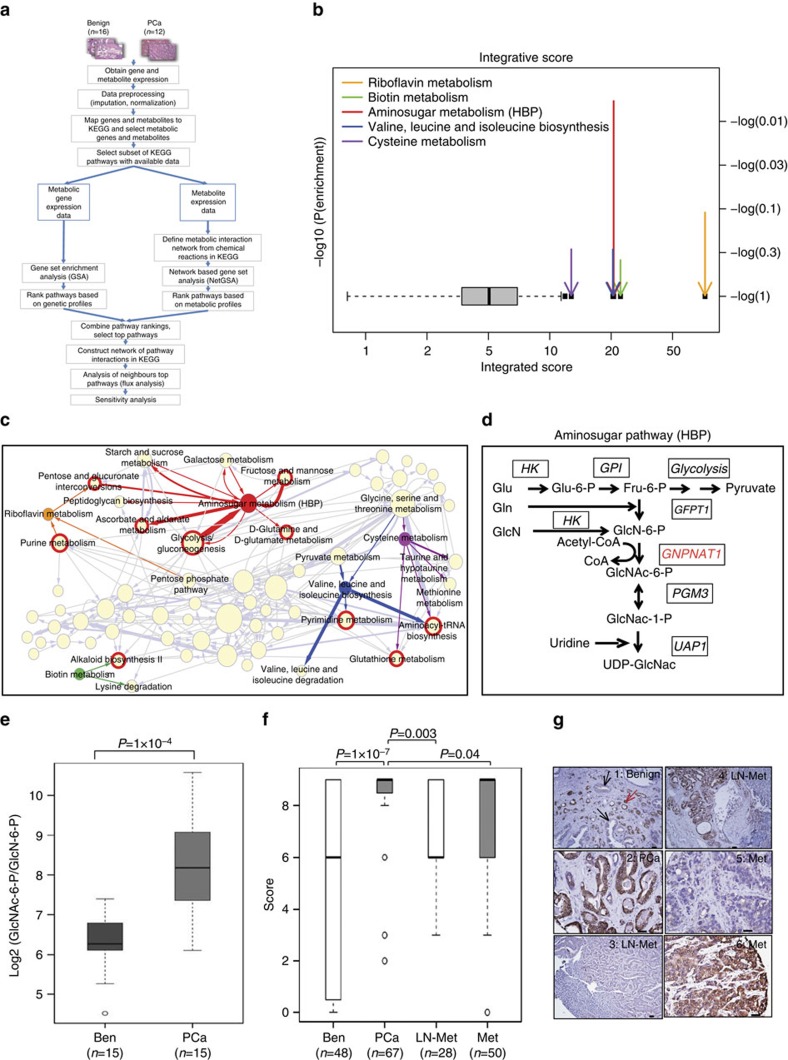
Integrative analysis of gene expression and metabolic data sets identifies alterations in the hexosamine biosynthetic pathway in prostate cancer. (**a**) Overview of integrative methodology. (**b**) Top pathways identified after integrative analysis using combined gene/metabolite-derived enrichment scores using our previously published^10^ data. Black dots indicate top six pathways identified as outliers and coloured arrows indicate the top five enriched pathways chosen for secondary analysis. (**c**) Network representation of pathways shown in **b** (solid coloured circles: enriched pathways after integrative analysis using combined gene/metabolite-derived enrichment scores; circumference is correlated to pathway connectivity). Association between interacting pathways and each of the enriched pathways (solid coloured circles) obtained after the integrative analysis is shown by coloured arrows, which also show the direction of interaction. Arrow thickness correlates with number of interacting components between two pathways. Enriched associated pathways (also termed interacting pathways) interacting with those listed in **b**, are shown in red rimmed circles. Thus, for example, amino sugar metabolism or HBP has eight interacting pathways, 5 of which are enriched (red rimmed circle). (**d**) Overview of the HBP. *GNPNAT1* (red) is the most proximal consistently upregulated HBP enzyme in PCa. (**e**) *GNPNAT1* product/substrate ratio was higher in PCa compared with matched benign-adjacent prostate tissues (*n*=15 pairs). *P*-value was calculated using Unpaired Student's *t*-test. (**f**) Boxplots showing immunostaining of *GNPNAT1* in primary PCa (*n*=67) versus benign adjacent (Ben, *n*=48), PCa versus lymph node mets (LN-Met, treatment naïve, *n*=28) and PCa versus CRPC mets (Met, *n*=50). *P*-value was calculated using Wilcoxon-Rank Sum test. (**g**) Representative photomicrograph of *GNPNAT1* staining in 1: Ben (black arrows) with tumour nodules (red arrow); 2: PCa; 3, 4: LN-Met and 5, 6: Mets. Representative scale bar for sections 1, 3 and 4 is 100 μm (low power) and for sections 2, 5 and 6 is 25 μm (high power). In all cases, *P*-value of <0.05 was considered significant. For boxplots, the horizontal line represents median value, whereas Whiskers represent either <25 or >75 quartile ranges.

To define key biochemical pathways altered in PCa, we used metabolomic and transcriptomic profiles from our previous study containing 12 treatment-naive localized PCa specimens and 16 benign adjacent prostate tissues (Ben)^10^ (Supplementary Fig. 1A, clinical information in Supplementary Table 1) and integrated these using a novel pathway-centric analytical framework (Fig. 1a). This approach combines two rankings for each pathway calculated from gene expression and metabolic data, while adjusting for variations in each of the two data sets. In particular, we used methods of Gene Set Analysis (GSA^11^,^12^) and a modified version of Network-Based Gene Set Analysis (NetGSA)^13^ to obtain rankings of each pathway based on genetic and metabolic data, respectively (overview in Fig. 1a). The modified NetGSA framework, unlike GSA-type methods, incorporates reactome-derived interactions and associated stoichiometry between metabolites allowing for adequate statistical power^13^.

The rankings of biochemical pathways enriched independently by gene expression and metabolomic profiles were calculated (Supplementary Fig. 1B) and combined into a single significance score (Fig. 1b) using a resampling procedure (see the Methods). Importantly, the individual rankings for the biochemical pathways derived from gene-expression and metabolomics data showed ∼65% concordance (as determined by calculating the number of pathways within one standard deviation of all pathway ranking differences shown in Supplementary Fig. 1C). The top five biochemical pathways enriched using the combined significance score are: riboflavin metabolism, biotin metabolism, amino sugar metabolism (also known as the Hexosamine Biosynthetic Pathway; HBP), valine-leucine and isoleucine biosynthesis and cysteine metabolism (Fig. 1b, *P*-values in Supplementary Table 2).

Next, to further select a pathway from those obtained using our integrative approach for follow-up functional studies, we employed a network-based permutation test using transcriptomics data to identify pathways that have the highest likelihood of playing a key role in coordinated biochemical pathway activity. Accordingly, we carried out an enrichment analysis using transcriptome data focusing on pathways that interacted with our primary list of enriched pathways (Supplementary Fig. 1D). The corresponding enrichment score was used as a measure of the likelihood of coordinated activity among the interacting pathways. In this way, the primary pathway exhibiting the highest likelihood was nominated for validation studies. Results of this network-based permutation test, which assesses the likelihood of observing particular connectivity patterns in a given network, are shown in Fig. 1b. HBP exhibited the smallest *P*-value (*P*=0.0060) among the top five pathways interrogated (Fig. 1b,c and Supplementary Table 3). This suggests that significant alterations in HBP are associated with PCa. Further, these results also imply that pathways interacting with HBP, namely glycolysis/gluconeogenesis, pentose and glucuronate interconversions, fructose and mannose metabolism, ascorbate and aldarate metabolism and glutamine and glutamate metabolism (Fig. 1c), are also important in PCa.

### HBP activity is downregulated in CRPC compared with AD PCa

HBP, a nutrient sensing pathway^14^, generates amino sugars by combining elemental carbon and nitrogen from glucose and glutamine (Fig. 1d). Glutamine-Fructose-6-phosphate transaminase 1 (*GFPT1*) has been described as the rate-limiting enzyme for this pathway^15^,^16^. The acetylation step represents a key entry point for carbon and nitrogen into the HBP and is catalysed by glucosamine-phosphate N-acetyltransferase 1 (*GNPNAT1*, Fig. 1d). UDP-N acetyl glucosamine pyrophosphorylase 1 (*UAP1*) catalyses the final enzymatic reaction of HBP, resulting in the synthesis of UDP-GlcNAc, that is required for N-linked and O-linked glycosylation reactions^17^,^18^.

*In silico* analysis showed that *GNPNAT1* and *UAP1* were significantly elevated in PCa compared with Ben in multiple publically available gene expression data sets including the one used here for integrative analysis (Supplementary Fig. 2A–C). *GNPNAT1* and *UAP1* were also significantly elevated at the transcript level (Supplementary Fig. 2D) in PCa. Further, increased activity of HBP in PCa was confirmed in 15 matched tumour–benign pairs by measuring the product to substrate ratio for the reaction carried out by *GNPNAT1* (N-acetylglucosamine-6-P to glucosamine-6-P; Fig. 1e) and levels of UDP-GlcNAc (Supplementary Fig. 2E), the end product of HBP. Tissue microarray analysis further confirmed significantly higher expression of both GNPNAT1 and UAP1 in PCa compared with Ben, whereas, interestingly, their expression was significantly lower in sites of lymph node metastasis and CRPC tissues compared with localized PCa (Fig. 1f,g and Supplementary Fig. 3A). Consistent with the findings in CRPC, transcript levels of HBP genes were also significantly downregulated in CRPC tissues across multiple independent publically available microarray data sets (Supplementary Fig. 3B–E). Together, these results suggest significantly diminished activity of HBP in CRPC tumours compared with AD organ-confined PCa.

### HBP modulates aggressivity in CRPC

Next, we studied the role of HBP in CRPC progression by examining the role of the proximal enzyme GNPNAT1 previously observed to be consistently downregulated in CRPC. Towards this end, we utilized a short hairpin RNA (shRNA) strategy to knockdown (KD) *GNPNAT1* expression in three CRPC-like (that is, androgen responsive, but not AD) cell lines LNCaP-ABL^19^ and C4-2 (ref. 20; containing AR-FL) and 22Rv1 (containing AR-FL and AR-V7) (ref. 21).

**Figure 2:**
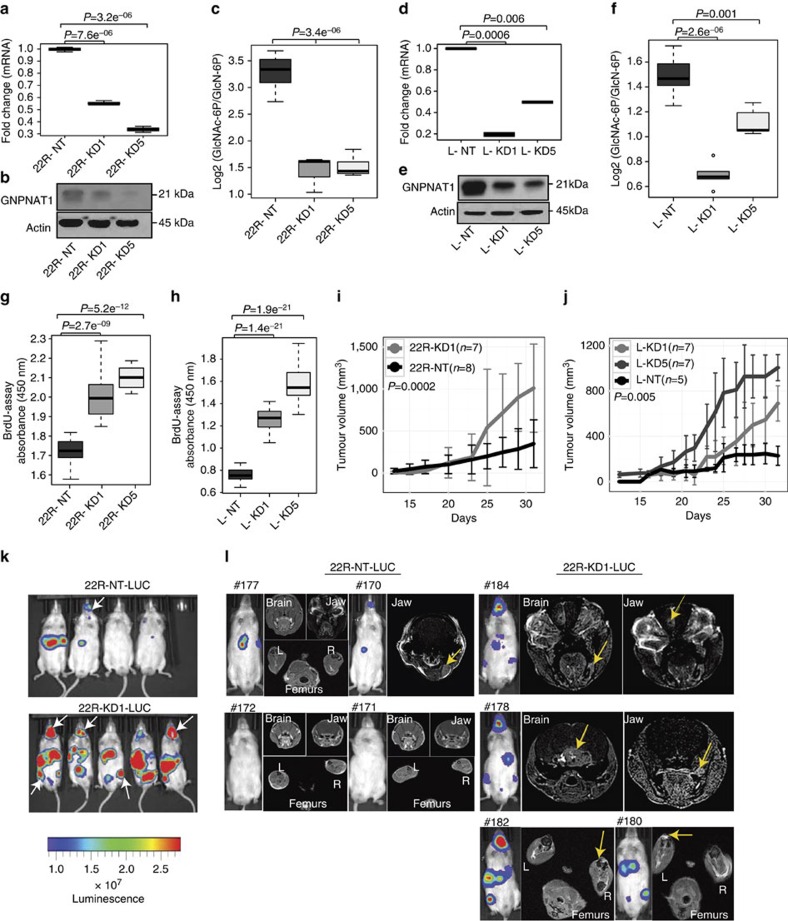
Effect of *GNPNAT1* knockdown in CRPC-like cells. shRNA knockdown (KD) of *GNPNAT1* in 22Rv1 cells (22R-KD1/5) leads to (**a**) significant reduction in mRNA (*n*=5, representative data), (**b**) loss of protein (∼21 kDa, *n*=5, representative immunoblot) and (**c**) significant decrease in product/substrate ratio (4–5 biological replicates) for *GNPNAT1*, compared with controls (22R-NT). Similarly, KD of *GNPNAT1* in LNCaP-ABL cells (L-KD1/5) leads to (**d**) significant reduction in mRNA (*n*=3, representative data), (**e**) loss of protein (*n*=3, representative immunoblot) and (**f**) significant decrease in product/substrate ratio (5 biological replicates) for *GNPNAT1*, compared with controls (L-NT). (**g**) *GNPNAT1* KD in 22Rv1 cells significantly increases proliferation compared with NT controls (5 biological replicates, 12 technical replicates). (**h**) Same as in **g**, but for *GNPNAT1* KD in LNCaP-ABL cells compared with NT controls (3 biological replicates, 20 technical replicates). (**i**) 22Rv1 xenograft tumours containing *GNPNAT1* KD show a significantly higher growth rate compared with controls (*n*=7 KD1 and *n*=8 NT). *Y* axis represents median tumour volumes in mm^3^ and associated median absolute deviation (MAD) for each group. (**j**) Same as in **i**, but for LNCaP-ABL cells containing *GNPNAT1* KD (L-KD1 and L-KD5, 7 mice per condition) and NT controls (L-NT, 5 mice). L-KD cells grew significantly faster than NT controls. (**k**) Representative images (*n*=4 NT, *n*=5 KD1) showing intra-cardiac injection of luciferase containing 22Rv1 cells with *GNPNAT1* KD or NT controls in castrated Nod-SCID Gamma mice (*n*=8 NT, *n*=10 KD1) showed significantly higher soft tissue metastasis. As shown in the representative figure, 4/5 KD mice and only 1/4 NT mice demonstrated metastatic lesions to the jaw and pelvic bones (white arrows). (**l**) Magnetic resonance imaging of representative mice shown in **k**. Yellow arrows show regions of metastasis in the brain as well as bones in the jaw and femur (L=left, R=right) supporting the luciferase data shown in **k**. ANOVA model was used to compute *P*-values for panels shown in **a**,**c**,**d**,**f**–**h**. For **i**,**j** showing *in vivo* data, a mixed effects linear model was fitted to the data as described under the Methods. For boxplots, the horizontal line represents median value, whereas Whiskers represent either <25 or >75 quartile ranges.

In 22Rv1 cells, *GNPNAT1* KD resulted in ∼50% and 70% reduction in mRNA (KD1 and KD5, respectively, Fig. 2a), decrease in protein expression (Fig. 2b and Supplementary Fig. 16), significant (*P*<0.05, analysis of variance (ANOVA)) reduction in the ratio of its product to substrate (Fig. 2c) as well as significant reduction in the levels of HBP end product UDP-GlcNAc (Supplementary Fig. 4A). Similar results were obtained in LNCaP-ABL (Fig. 2d–f and Supplementary Figs 4B and 16) and C4-2 cells (Supplementary Figs 4C–E and 19). All these cells exhibited a significant (*P*<0.05, ANOVA) increase in proliferation (Fig. 2g,h and Supplementary Fig. 4F–H), associated with no evidence of apoptosis as assessed by Poly-ADP ribose polymerase cleavage (Supplementary Figs 4I and 19) compared with their respective non-target (NT) controls; this was reversed by overexpression of *GNPNAT1* (Supplementary Fig. 4J,K). In contrast, in AD LNCaP cells, KD of *GNPNAT1* resulted in a significant (*P*<0.05, Student's *t*-test) reduction in cell proliferation, which was partially rescued by add back of HBP metabolite UDP-GlcNAc (Supplementary Figs 5A–C and 20), all of which verify our earlier observation describing elevated levels of HBP in AD PCa.

*In vivo* xenograft experiments using 22Rv1 KD cells without (Fig. 2i) or with luciferase (Supplementary Figs 6A,B), demonstrated a significantly (*P*<0.05, GLM or generalized linear model) higher rate of tumour growth compared with NT controls. Morphometric analysis revealed KD tumours to be larger, whereas both quantitative PCR (QPCR) and immunoblot analysis confirmed KD of GNPNAT1 (Supplementary Figs 6C–E and 21). Further, histological analysis revealed KD tumours to be slightly more pleomorphic, with loss of glandular architecture and significantly higher number of mitotic figures compared with their NT counterparts (Supplementary Fig. 6F). Moreover, KD cells in LNCaP-ABL (Fig. 2j) also showed a significant increase in their tumorigenic potential. Interestingly, ∼100 days post resection of primary LNCaP-ABL tumours (refer Fig. 2j for details), five of six mice (two NT and three KD5) developed metastatic lesions in lymph nodes. Metastatic tumours in KD mice were at least threefold bigger (*P*<0.038, Student's *t*-test) in volume (360–880 mm^3^) compared with the NT (15–100 mm^3^ group; Supplementary Fig. 7A,B). Consistent with this, intra-cardiac injection of luciferase-labelled 22Rv1 cells in castrated Nod-SCID Gamma mice demonstrated significantly higher number of metastatic lesions in the KD compared with NT mice (*P*<0.01, Student's *t*-test, Fig. 2k and Supplementary Fig. 7C, *n*=8 NT, *n*=10 KD1). Interestingly, additional metastasis to the brain as well as bones in the jaw and pelvic regions was observed in eight out of ten KD and only one out of eight NT mice (representative pictures shown in Fig. 2k white arrows and quantified in Supplementary Fig. 7C). Magnetic resonance imaging (MRI, on *n*=4 from each group) using Prohance contrast agent further confirmed brain metastases as well as jaw and pelvic bone disease (Fig. 2l, yellow arrows).

To further verify the tumour-promoting role of HBP pathway, we knocked down *GFPT1* either transiently or stably in CRPC-like cells. Consistent with our *GNPNAT1* KD findings, KD of *GFPT1* using short interfering RNA (siRNA) in 22Rv1 and LNCaP-ABL cells resulted in increased cell proliferation (Supplementary Figs 8A–E and 22). This was further replicated in 22Rv1 cells using shRNA-based stable KD of *GFPT1* (Supplementary Fig. 8F,G). Interestingly, stable KD of *GFPT1* in CRPC-like cells also resulted in a significant (*P*<0.05, Student's *t*-test) reduction in mRNA levels of *GNPNAT1* (Supplementary Fig. 8F). Similarly, KD of *GNPNAT1* in CRPC-like cells also significantly (*P*<0.05, Student's *t*-test) decreased *GFPT1* expression, both at the mRNA and protein level (Supplementary Figs 8H,I and 23). These findings confirm a role for HBP pathway in promoting CRPC growth and proliferation.

### HBP affects CRPC via PI3K-AKT and SP1-ChREBP

**Figure 3:**
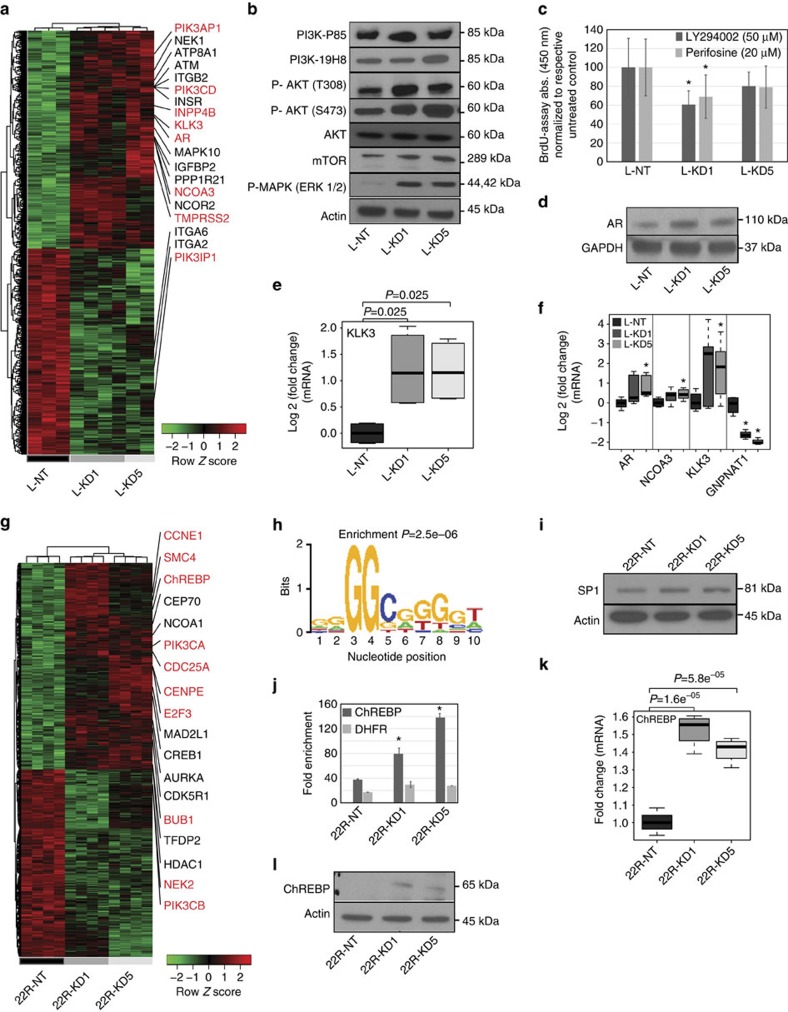
Elucidating the mechanism of *GNPNAT1* KD driving an aggressive phenotype in CRPC-like cells. (**a**) Heat map showing differential (*P*<0.05, FDR 30%) genes in LNCaP-ABL cells containing *GNPNAT1* KD (L-KD1, *n*=4; and L-KD5, *n*=2) compared with NT controls (L-NT, *n*=3). (**b**) Immunoblot showing increased expression of phosphorylated and total PI3K (∼85 kDa, *n*=3 for PI3K-P85 and *n*=2 for PI3K-19H8), AKT (∼60 kDa, *n*=4 for AKT, *n*=3 for p-AKT (T308) and *n*=4 for p-AKT (S473)), m-TOR (∼281 kDa, *n*=5) and p-MAPK (∼44-42 kDa, *n*=3) in LNCaP-ABL KD cells compared with controls. In all cases, representative immunoblots are shown. (**c**) Treatment of LNCaP-ABL NT and KD cells with either 20 μM Perifosine (AKT/PI3K inhibitor, (*n*=3) or 50 μM of LY294002 (PI3K) inhibitor (*n*=3), significantly decreased KD1 cell proliferation and modestly reduced KD5, both compared with treated NT control. (**d**) Immunoblot showing increased expression of AR in *GNPNAT1* KD LNCaP-ABL cells compared with control (∼110 kDa, *n*=3, representative immunoblot). (**e**) QPCR validating increased expression of AR-target gene *KLK3* (*PSA*) in LNCaP-ABL KD cells (*n*=3). (**f**) QPCR validating increased expression of *AR* and its co-activator *NCOA3* and downstream target *KLK3* in xenograft tumours derived from LNCaP-ABL KD cells compared with NT control (*n*=4). (**g**) Heat map showing differential (*P*<0.05, FDR 20%) genes in 22Rv1 cells containing *GNPNAT1* KD (22R-KD1 and 22R-KD5, *n*=4 each) compared with NT controls (22R-NT, *n*=4). (**h**) Promoter Scan on genes upregulated in KD cells revealed enrichment for SP1-binding sites. (**i**) Immunoblot showing significantly (*P*<0.05) increased protein levels for SP1 (∼81 kDa, *n*=5) in 22Rv1 cells containing *GNPNAT1* KD (22R-KD1 and 22R-KD5, median band intensity of 0.66±0.23 and 0.51±0.08, respectively) compared with NT control (median band intensity of 0.35±0.06). Actin was used to control for protein loading. (**j**) Chromatin immunoprecipitation (ChIP) assay-coupled to QPCR to verify SP1 binding to the promoter of ChREBP. SP1 binding to the promoter of DihydroFolate Reductase (DHFR) was used as a positive control (*n*=3, median and standard deviation shown). (**k**) QPCR validation of *ChREBP* expression in 22Rv1 KD and NT cells (*n*=3). (**l**) Immunoblot showing elevated protein expression of ChREBP in 22Rv1 KD cells compared with NT control (∼65 kDa, *n*=3, representative immunoblot). *P*-values were computed using Unpaired Student's *t*-test (for **f**) and ANOVA model (**c**,**e**,**j** and **k**). For **e** and **f**, the fold change was log 2 transformed to get normal distribution. In all cases, *P*-value: *P<0.05. For boxplots, the horizontal line represents median value, whereas Whiskers represent either <25 or >75 quartile ranges. All bar plots are represented in median±s.d.

Analysis of microarray data in LNCaP-ABL KD cells compared with control revealed a set of upregulated genes indicative of elevated expression of components of AKT signalling namely *PIK3CD* and *PIK3AP1* (Fig. 3a; GEO ID: GSE67537). In LNCaP-ABL KD cells, increased protein expression of activated PI3K (PI3K-p85), total AKT, p-AKT (T308), p-AKT (S473) and elevated levels of its downstream effectors mTOR and p-MAPK (ERK1/2) were observed (Fig. 3b and Supplementary Fig. 17). Consistent with this, treatment of LNCaP-ABL KD and NT cells with the reversible PI3K inhibitor LY294002 (ref. 22; 50 μM) and the AKT/PI3K inhibitor Perifosine^23^ (20 μM) resulted in a significant (*P*<0.05, Student's *t*-test) reduction in cell proliferation in KD1 and modest but not significant reduction in KD5 cells compared with NT controls. In addition, the upregulated gene expression signature for LNCaP-ABL KD cells revealed increased expression of *AR*, the AR co-activator *NCOA3* and AR target genes (*KLK3* and *TMPRSS2*; Fig. 3a). Elevated expression of AR at the transcript (Supplementary Fig. 9A) and protein (Fig. 3d and Supplementary Fig. 17) levels as well as *KLK3* (Fig. 3e) and *TMPRSS2* (Supplementary Fig. 9B) was verified in LNCaP-ABL KD cells and their corresponding xenograft tumours (Fig. 3f). A similar cascade of molecular alterations involving the PI3K-AKT axis was observed in C4-2 KD cells, which contain full-length AR (Supplementary Figs 10A,B,24 and 25).

In contrast, the upregulated genes in the microarray data set (GEO ID: GSE67537) obtained from 22Rv1 cells containing *GNPNAT1* KD revealed increased expression of multiple cell cycle genes (Fig. 3g, red highlight) that together enriched multiple cell cycle concepts (Supplementary Fig. 11A). Elevated expression of a subset of these cell cycle genes was verified by QPCR (Supplementary Fig. 11B) and supported our observation of increased proliferation seen *in vitro* and *in vivo* upon KD of *GNPNAT1*. Furthermore, unlike in LNCaP-ABL KD cells, AR protein expression (both AR-FL and AR-V7) was unchanged upon *GNPNAT1* KD in 22Rv1 cells (Supplementary Figs 11C and 26). Intriguingly, however, in 22Rv1 cells, transient KD of *GFPT1* modestly increased protein expression of AR-FL and AR-V7 (Supplementary Figs 8B and 22) and its stable KD significantly (*P*<0.05, Student's *t*-test) increased the mRNA levels for *AR-FL* and *AR-V7* (Supplementary Fig. 8F).

To obtain insight into the regulatory components driving the upregulated gene signature in *GNPNAT1* KD 22Rv1 cells, promoter analysis using Pscan^24^ revealed significant enrichment of SP1-binding sites (Fig. 3h and Supplementary Fig. 11D). Corroborating this, overall protein expression of SP1 was significantly (*P*<0.05, Student's *t*-test) increased in 22Rv1 KD cells (Fig. 3i and Supplementary Fig. 18). *In silico* analysis confirmed higher expression of SP1 in metastatic PCa tissues compared with primary tumours and benign prostate (Supplementary Fig. 11E). These findings are consistent with earlier reports that suggest negative regulation of SP1 activity by glycosylation^25^. Consistent with this, global assessment of O-glycosylation using immunoblot analysis in both 22Rv1 and LNCaP-ABL cells containing *GNPNAT1* KD revealed reduced glycosylation in a subset of proteins (Supplementary Figs 12A,C,E,27 and 28). This altered glycosylation profile was reversed in a number of proteins by add back of HBP end product UDP-GlcNAc (60 mM, 72 h, Supplementary Figs 12B,D,E,27 and 28).

We next asked how cell cycle genes could be regulated by SP1 in 22Rv1 KD cells. Our prior integrative analysis (Fig. 1c) revealed changes in glycolysis to be associated with changes in HBP. Earlier studies have demonstrated a link between carbohydrate response element-binding protein (ChREBP) and glycolytic intermediates (for example, xylulose-5-P) (ref. 26). Using LC-MS, we detected high levels of ribulose-5-P (Supplementary Fig. 13A), a structural isomer of xylulose-5-P, whereas chromatin immunoprecipitation (ChIP)-QPCR analysis demonstrated significantly (*P*<0.05, Student's *t*-test) increased binding of SP1 on the *ChREBP* promoter (Fig. 3j). Consistent with all of the above, mRNA microarray (Fig. 3g) and QPCR (Fig. 3k), protein (overall, Fig. 3l and Supplementary Fig. 18 nuclear and cytoplasmic, Supplementary Figs 13B and 29), combined nuclear and cytoplasmic staining (Supplementary Fig. 13C,D) and activity (Supplementary Fig. 13E) of ChREBP were elevated in 22Rv1 KD cells. A similar increase in *ChREBP* expression was also observed with KD of *GFPT1* in 22Rv1 cells (Supplementary Fig. 8F). These findings suggest a role for HBP in regulating ChREBP expression in CRPC-like cells. Moreover, a subset of cell cycle-associated genes previously shown to be transcriptional targets of ChREBP^27^ was also identified in the upregulated gene expression signature associated with *GNPNAT1* KD in 22Rv1 cells (Supplementary Fig. 13F). This suggests an important role for ChREBP in regulating proliferation in CRPC-like cells upon modulation of HBP. Consistent with this, increased expression of ChREBP was also observed in human metastatic PCa tissues (Supplementary Fig. 13G).

**Figure 4:**
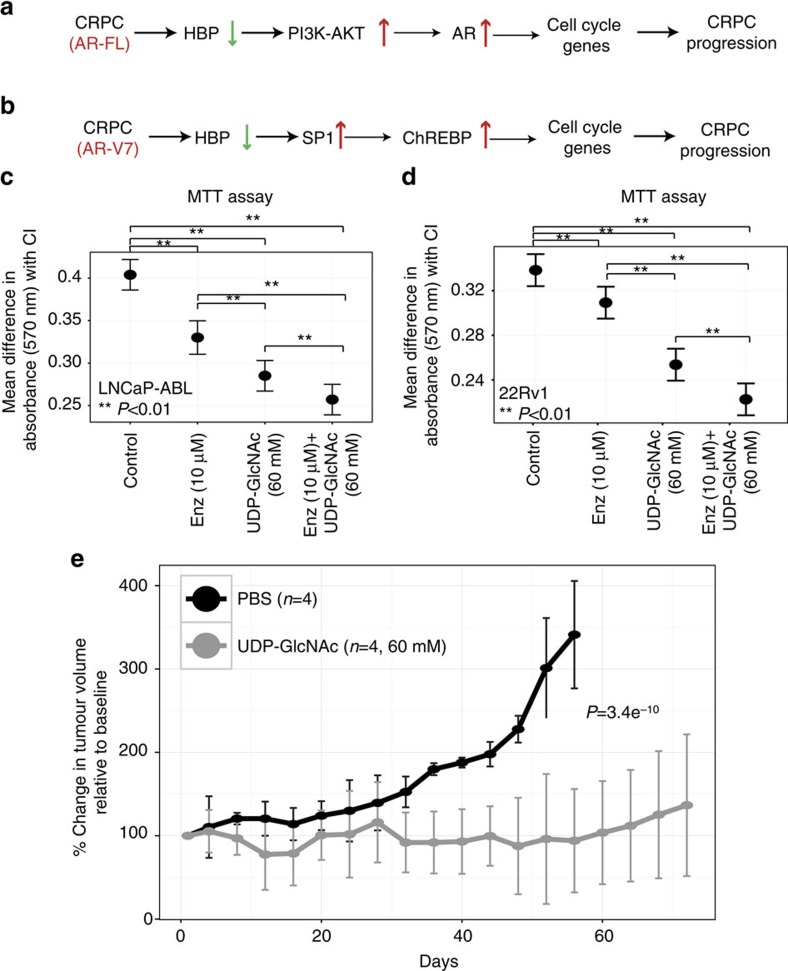
Therapeutic targeting of HBP in CRPC. (**a**) Decreased HBP expression in CRPC tumours containing AR-FL is associated with increased activity of PI3K-AKT that could activate AR. This activates cell cycle genes leading to increased proliferation driving CRPC progression. (**b**) In CRPC containing AR-V7, decreased HBP expression activates SP1-regulated ChREBP expression leading to stimulation of cell cycle genes, increased proliferation and tumour progression. (**c**) MTT assay results for LNCaP-ABL (*n*=9) cells treated with vehicle (PBS+dimethylsulphoxide) or 10 μM enzalutamide (Enz) or 60 mM UDP-N-acetylglucosamine (UDP-GlcNAc) or both enz+UDP-GlcNAc for 96 h. (**d**) Same as in **c** but treatments were done on 22Rv1 cells (*n*=8). In both **c** and **d**, differences in the level of mean absorbance with corresponding confidence interval (CI) to control obtained for each of the conditions (*x* axis) is represented on the *y* axis. Comparisons of all treatments with control group in **c**,**d** were significant at a *P*-value of 0.01 (Bonferroni corrected). Pairwise comparisons of either Enz or combination of Enz+UDP-GlcNAc with other treatments were significant at a *P*-value<0.05. Treatment with Enz resulted in a modest to pronounced decrease in proliferation of 22Rv1 and LNCaP-ABL cells, respectively. Compared with Enz, treatment with UDP-GlcNAc alone resulted in a significant reduction in cell proliferation in both the CRPC-like cells. Furthermore, the effect of Enz+UDP-GlcNAc was significantly higher in CRPC-like cells, compared with any single treatment. Error bars represent s.e.m. (**e**) Plot showing rate of growth of LNCAP-ABL xenograft tumours (set 1, see the Methods for details) treated bi-weekly with either 60 mM UDP-GlcNAc (grey lines, *n*=4 mice) or PBS (black lines, *n*=4 mice), for a period of 72 days. For each animal, the tumour volume at each time point was normalized to its baseline value obtained before starting the treatment. The percent change in tumour volume for all the animals in group was calculated along with the associated median absolute deviation (MAD) and plotted against time (in days). *P*-value was calculated using GLM model as described in the text. Importantly, mice treated with UDP-GlcNAc showed a significant reduction in rate of tumour growth compared with the control-treated tumours (*P*-value=3.4e^−10^). Error bars represent MAD.

Collectively, the above results imply that inherently decreased HBP activity in CRPC cells modulates proliferation via PI3K-AKT (in CRPC containing full-length AR; Fig. 4a) or SP1-ChREBP (in CRPC possessing AR-V7; Fig. 4b) to drive CRPC progression.

### UDP-GlcNAc has therapeutic efficacy in CRPC

As CRPC cells innately show reduced expression and activity of HBP components, which in turn activates the cell cycle, we hypothesized that addition of a HBP downstream metabolite could slow down proliferation and reduce cell viability. Accordingly, addition of 60 mM UDP-GlcNAc for 96 h to both 22Rv1 and LNCaP-ABL cells resulted in a significant decrease in both viability and cell numbers by MTT and Celigo-based quantification assay (Bonferroni corrected *P*=0.01, Fig. 4c,d and Supplementary Fig. 14A,B), with a concomitant downregulation of predicted cell cycle genes (Supplementary Fig. 14C, in 22Rv1 cells). Mass spectrometry-based quantification confirmed internalization of UDP-GlcNAc into both LNCaP-ABL and 22Rv1 cells (Supplementary Fig. 14D). A similar significant reduction in cell numbers was obtained when the CRPC-like cells were treated either with 10 mM glucosamine (GlcN, 96 h, Supplementary Fig. 14A,B) or with 20 mM UDP-GlcNAc (96 h, Supplementary Fig. 14A,B), whereas no effect on cell numbers was observed when exposed to 20 mM mannose, an unrelated metabolite (96 h, Supplementary Fig. 14A,B).

This motivated us to combine 60 mM UDP-GlcNAc with the anti-androgen enzalutamide (a drug commonly used to treat men with CRPC^28^) to test whether the metabolite could improve efficacy of anti-androgen therapy on CRPC cells. As expected, addition of 10 μM enzalutamide to LNCaP-ABL for 96 h resulted in a modest reduction in cell numbers (Celigo, Supplementary Fig. 14A) and a pronounced decrease in viable cells, both of which were significant (MTT: Fig. 4c). Similarly, treatment of *AR*-*V7*-containing 22Rv1 cells with 10 μM enzalutamide for 96 h had negligible effect on cell numbers (Celigo: Supplementary Fig. 14B) but a statistically significant effect on viability (MTT: Fig. 4d). This modest cytostatic effect seen in 22Rv1 cells was primarily due to inhibition of AR signalling by enzalutamide as evidenced by significant reduction in expression of two candidate AR targets, *PSA* and Hexokinase 2 (*HK2*, Supplementary Fig. 14E). Furthermore, combination of 20 or 60 mM UDP-GlcNAc with 10 μM enzalutamide displayed a significant additive cytostatic effect in both 22Rv1 and LNCaP-ABL cells (MTT assay: Fig. 4c,d and Celigo assay: Supplementary Fig. 14A,B), compared with vehicle-treated controls.

As our findings indicated a potential therapeutic value for the metabolite alone, we tested its efficacy on CRPC-like xenograft tumours *in vivo*. Intra-tumoral injection of 60 mM UDP-GlcNAc followed by mass spectrometry analysis of the CRPC xenografts showed the presence of 4–8 mM levels of the metabolite up to 48 h post injection (Supplementary Fig. 14F). Furthermore, treatment of LNCaP-ABL xenograft tumours with 60 mM UDP-GlcNAc bi-weekly resulted in a significant (*P*<0.05, GLM) reduction in the rate of tumour growth compared with vehicle controls or relative to an unrelated metabolite, 60 mM mannose (Fig. 4e and Supplementary Fig. 15A,B).

Overall, our novel integrative analysis takes into account pathway-based evidence, obtained independently from gene expression and metabolic compartments. This method overcomes the potential lack of agreement between data points in transcriptomics and proteomics/metabolomics data sets, which has been an inherent bottleneck for concordance-based integrative approaches^29^. Our method relies on networks of reactomes that constitute pathways, as well as the interactions of these pathways with each other. Based on this approach, we identified HBP as a key biochemical mediator of CRPC progression. HBP-associated metabolic re-wiring observed in CRPC supports cell cycle via PI3K-AKT/SP1-ChREBP-axis. Further, replenishing the HBP in these cells via the addition of a downstream metabolite rescues the KD phenotype. We observed a contrasting effect of HBP on growth of AD PCa and CRPC cells further suggesting the existence of metabolic re-wiring during PCa progression. Notably, HBP has been known to be a nutrient sensor and shown by others to regulate glycolysis^30^. In light of this, one may speculate that reduction in HBP in CRPC could be a selective adaptation to the increased need for glycolysis required to meet the bioenergetic demands of an invasive tumour. This is supported by our recent publication that describes increased glycolysis as one of the main metabolic adaptations seen in CRPC-like cells^31^. Provocatively, treatment of CRPC with HBP metabolites UDP-GlcNAc and GlcN, *in vitro* and UDP-GlcNAc *in vivo*, significantly reduced the proliferation of CRPC-like cells. The therapeutic effect *in vitro* was further enhanced by combining the metabolite with the anti-androgen enzalutamide. These findings are particularly noteworthy given that CRPC cells containing the AR-V7 variant (that is, 22Rv1) are essentially resistant to enzalutamide. Although preliminary, our results introduce the innovative concept of using metabolites to overcome therapeutic resistance in advanced tumours via the exploitation of altered metabolic pathways.

## Methods

### Cell culture and lentiviral transduction or siRNA transfection

Human prostate cell line 22Rv1 was purchased from American Type Culture Collection, C4-2 and LNCaP-ABL cells were obtained from Drs Nancy Weigel and Nicholas Mitsiades, respectively. LNCaP cells were obtained from the Tissue Culture Core at Baylor College of Medicine (BCM). All cell lines were verified using Short-Tandem Repeat DNA fingerprinting at the MD Anderson Cancer Center and were tested negative for mycoplasma contamination using MycoAlert Detection Kit (Lonza, Cat# LT07-418). C4-2 cells were grown in T-media with 10% fetal bovine serum (FBS), whereas 22Rv1, LNCaP and LNCaP-ABL were grown in RPMI-1640 medium. Both 22Rv1 and LNCaP were grown in 10% FBS and phenol red, whereas LNCaP-ABL was grown in 10% charcoal stripped FBS without phenol red. One hundred micrograms per millilitre of Normocin was added to all the cell culture media. To generate stable *GNPNAT1* and *GFPT1* KD in CRPC-like cells, lentiviral transduction using shRNA (Sigma Aldrich, Product# SHCLNV-NM_198066 (*GNPNAT1*, sequence in Supplementary Table 6), SHCLNV-NM_002056 (*GFPT1*, sequence in Supplementary Table 6), two independent shRNA clones each) targeting these genes was carried out at a viral titre of 5 MOI. Cells with stable KD of the gene were cultured in respective media added with 0.5 μg ml^−1^ of puromycin. In addition, where indicated, GFPT1-specific siRNA from Life Technologies (Cat numbers SASI_Hs01_00162618, SASI_Hs01_00162619 and SASI_Hs02_00333099) was used to create transient KDs according to the manufacturer's protocol. Similarly, luciferase (GenTarget Inc, cat# LVP402-PBS) expression in 22Rv1 cells was achieved using a virus titre of 2 MOI and cells selected using 800 μg ml^−1^ Geneticin. Overexpression vector for GNPNAT1 was obtained from the Cell-Based Assay Screening Service core at BCM.

### Integrative analysis and biostatistics

The data set used in this study are supplied as Supplementary Data 1. For the integrative analysis, gene expression and metabolomics data for benign adjacent prostate tissue and localized PCa were used. Gene expression and metabolic data were filtered, imputed and normalized as described^32^.

An integrated analysis of Kyoto Encyclopedia of Genes and Genomes (KEGG) pathways based on gene and metabolic expression data was performed^13^,^33^,^34^ as described below. Briefly, pathway enrichment based on gene expression and metabolic data was assessed using GSA (a variant of Gene Set Enrichment Analysis (GSEA) with improved power properties^33^) and NetGSA^13^,^34^, respectively. NetGSA belongs to the class of network topology-based pathway enrichment methods^35^ and incorporates information about connections between biomolecules (genes, proteins and metabolites) in a mixed effects linear regression model^13^,^34^. This mixed effects linear regression model allows for a rigorous testing procedure for pathway enrichment. In our earlier study^13^,^34^ using NetGSA to enrich pathways using gene expression data, we used existing gene network databases, such as BioGrid, YeastNet and BioCarta, to create the interaction network. In contrast, to apply NetGSA to metabolomics data, biochemical reactions among metabolites from KEGG were used to define their respective interaction network. In this network, the nodes are metabolites and edges between metabolites are drawn based on information from biochemical reactions. In other words, an edge is drawn between two metabolites if they constitute substrate–product pair of at least one biochemical reaction.

Pathways were ranked separately based on enrichment results from the gene expression and metabolic data. Subsequently, a combined score was derived and normalized for the variability in rankings observed in each data set. To estimate the variability of the combined scores, a bootstrap resampling procedure^36^ was employed wherein data from the tissue samples (in PCa and Benign classes) were randomly sampled with replacement.

For the resampling procedure, we denote the *averag*e ranking of the pathways *j*=1,…87 in 1,000 bootstrap samples of the gene expression (*G*) and metabolic expression (*M*) using the terms *R(j,G)* and *R(j,M)* and their standard deviation using the terms *S(j,G)* and *S(j,M)*, respectively. The final integrative score for the *j*th pathway is then defined as the mean of the normalized rankings of each pathway across 1,000 bootstrap samples computed using the equation: R*(j)=[88*-R(j,G)/S(j,G)*]+[88*-R(j,M)/S(j,M)*]. It is worth noting that the procedure implicitly self-adjusts for signal strength and noise levels in the gene expression and metabolomics data sets, thus proving more powerful and robust than concordance methods^37^.

Upon examination of the distribution of the combined scores (Fig. 1b), four pathways were found to have a significantly larger integrative score (*P*<0.03) than the rest, based on a normal test for outliers (Supplementary Table 2). To ensure that potentially important pathways were not missed, an additional pathway namely Cysteine metabolism (*P*=0.2), which was a borderline outlier, was also included along with the top four enriched pathways for secondary analysis. Overall, we selected the top five pathways from the integrative analysis, termed henceforth as pathways derived from the ‘primary enrichment analysis'. In the next step, KEGG pathways interacting with these top five enriched pathways were determined using information obtained from KEGG.

Next, we examine whether the neighbours of these five pathways (obtained from our integrative analysis) are also enriched based solely on gene expression data (Fig. 1c). This is based on the premise that gene expression data capture the encoded functional potential of the cell and hence could reveal interacting pathways that are most likely to be activated in the near term.

Procedurally for this secondary analysis, we first constructed the interaction network between 87 KEGG-defined pathways. The nodes of this network are KEGG pathways and an edges was drawn between two pathways if gene/metabolites members of one pathway interacted with gene/metabolite members of the other. We then used this information to determine the number of interacting pathways for each of the top five pathways obtained using the primary enrichment analysis. A total of 20 interacting pathways were identified for the top five pathways derived from the integrative analysis. Following this, using gene expression data we looked for enrichment of these 20 interacting pathways and rank ordered them based on their enrichment score. From this list, we next selected the top ten interacting pathways (that is, pathways that were ranked within the top 50%) and asked how many of these interact with the top five pathways derived from the primary enrichment analysis (Supplementary Fig. 1D) and used a network-based permutation test to assess their significance. This procedure identified the HBP as the key pathway in PCa (Supplementary Table 3).

To carry out the network-based permutation test, we denoted by **N(1), …, N(5)** the number of enriched neighbours of each of the top five pathways based on the gene expression data. We then generated *B*=10,000 random networks by scrambling the patterns of interactions among pathways, while preserving the number of interactions for each pathway as defined based on KEGG (Fig. 1c). That is to say that the randomization process maintained the number of interacting nodes (pathways) and their degree distribution. Procedurally, this was done as follows. Let **N(1,b), …, N(5,b)** denote the number of enriched interacting nodes for the five pathways obtained from the integrative analysis above in the **b**-th generated random network. The significance measure for association of the interacting neighbours for the top five enriched pathways resulting from the original integrative analysis is determined using the equation *P(j)=1/B ∑ (N(j,b)>N(j))*, where *j*=1,…5 and *B*=10,000. Importantly, the proposed network-based permutation test preserves the topology of the pathway interaction network thus preventing inflated false positive errors. R software code used for integrative analysis is available as Supplementary Information.

### Microarray analyses

Human Affymetrix Human Genome U219 Array Plate was used for gene expression analysis with values for each gene being calculated using robust multi-array average^38^. Data were fitted in weighted linear models, which are designed specifically for microarray analysis^39^. The data were further filtered using principal component analysis to identify samples that do not cluster with the rest of the samples within a group. In LNCaP-ABL cells, this resulted in removal of three samples (one NT and two KD) from further analysis. ANOVA statistics was computed for each probe set, corrected for false discovery rate (FDR^40^) and mapped for over-represented pathways using ConsensusPathDB (http://cpdb.molgen.mpg.de).

### Metabolomic analyses

Mass spectrometry-based multiple reaction monitoring (MRM) studies were done as described^41^. A list of MRM transitions is given in Supplementary Table 4.

For the targeted metabolic measurements described in this manuscript, *P*-value was computed using *t*-test/ANOVA coupled to multiple comparison correction using FDR correction method described in FDRtool package in R software.

### Reagents used for metabolite analysis

Reagents such as acetonitrile, methanol and water for high-performance liquid chromatography were purchased from Burdick and Jackson. Formic acid and internal standards, which included a mixture of Jasmonic acid, [^15^N]_2_-Tryptophan, Thymine-d4, Glutamic acid-d5, Gibberellic acid, Trans-Zeatin, [^15^N]-Anthranilic acid and Testosterone-d3, were purchased from Sigma-Aldrich. For UDP-N-Acetyl glucosamine quantification, [^13^C]_6_ N-acetyl glucosamine was used as an internal standard and the calibration curve for quantification was generated using the purified metabolite purchased from Sigma-Aldrich.

### Quality controls for metabolic analysis

Aliquotes of isotopic labelled internal standards described above were used at a final concentration of 0.25 mM. Instrument performance was monitored using matrix-free mixture of internal standards reconstituted in a 50:50 (v/v) mixture of methanol and water. Furthermore, for relative quantification of metabolite levels, isotopic labelled internal standards spiked into the samples were used to control for variation in metabolite extraction procedure. Along with experimental samples, we also extracted the metabolites from pooled liver samples (spiked with internal standards), which were analysed in tandem with experimental samples. This allowed us to control for variation from both extraction procedure and instrument performance. Similarly, for absolute quantification of UDP-N-acetyl glucosamine, calibraton curve was generated by spiking in [^13^C]_6_ N-acetyl glucosamine in a matrix containing dialysed tissue extract.

### Metabolite extraction

The metabolite extraction protocol used in this study was described earlier^32^. Briefly, both cell line and tissue samples were either sonicated or homogenized, respectively, in methanol/water (4:1) containing mixture of internal standards. Before this step, the cell line samples were subjected to multiple freeze–thaw cycles to achieve homogenous rupture of cell membranes. The cell line or tissue homogenate was then diluted with 450 μl of ice-cold chloroform and vortexed using a Multi-Tube Vortexer for 10 min. Following this, 150 μl of ice-cold water was added to the homogenate and vortexed for an additional 2 min. The homogenate was then incubated at −20 ° C for about 20 min to allow for aqueous and organic phase separation. Following separation of organic and aqueous phases, each of them was dried at 37 °C for 45 min using EZ-2 series Genevac Speed Vac system. Next, the dried aqueous samples were reconstituted in 50:50 mixture of methanol/water and subjected to de-proteination using a 3-kDa molecular filter at 4 °C for 90 min. Following this, the filtrate was dried, reconstituted in 50:50 mixture of methanol/water containing 0.1% formic acid and separated by liquid chromatography before mass spectrometry analysis.

### Liquid chromatography and mass spectrometry analysis

Separation of metabolites prior mass spectrometry was carried out by reverse phase chromatography on a Phenomenex Synergi Max-RP (250 × 4.6 mm^2^, 80 Å, Phenomenex) column using an Agilent 1290 series HPLC system (Agilent Technologies). Mobile phase used was composed of 1 mM ammonium acetate with 0.05% ammonium hydroxide/water (v/v; solvent A) and 1 mM ammonium acetate with 0.05% ammonium hydroxide/acetonitrile (v/v; solvent B). Gradient parameters used for the separation were: 0 min-5% B; 15–17 min-10% B, 22 min-90% B, 23–28 min-5% B, followed by re-equilibration to the initial starting condition. Flow rate of 0.3 ml min^−1^ was optimized for the experiments.

Relative or absolute levels of metabolites were quantified using MRM (refer Supplementary Table 4 for MRM transitions). MRM-based mass spectrometry analysis was performed on 6490 QQQ-LC/MS (Agilent Technologies) using electrospray ionization. Both positive and negative ionization modes were employed using capillary voltages of 3,500 V (positive ionization) and −3,000 V (negative ionization), respectively, a collision gas flow rate of 10 l min^−1^ and a nebulizer gas flow rate of 35 l min^−1^. Throughout these experiments, nebulizer gas temperature was maintained at 350 °C. Nitrogen was used as the collision gas at a collision cell pressure of 2.39 × 10^−5^ Torr. For maximum sensitivity, parameters for fragmentor voltage and collision energy for each of the metabolites assessed were optimized using the optimizer software (Agilent Technologies).

### RNA and QPCR analysis

Standard RNA extraction (RNeasy Mini Kit from Qiagen), reverse transcription (Quanta Biosciences, cat# 95048-500) and SYBR green (Life Technologies, cat # 4385614) Q-PCR were performed^41^. Primers used in this study are listed in Supplementary Table 5.

### Western blot

Antibodies to GNPNAT1 (Proteintech, cat # 16282-1-AP, 1:2,000), GFPT1 (Santa Cruz Biotechnology, cat# sc-377479), SP1 (Cell Signaling, cat # 9389, 1:1000), ChREBP (Novus, cat # NB400-136V, 1:1,000), AR (Santa Cruz Biotechnology, cat # sc-816, sc-7305), AR-V7 (Precision Antibody, cat # AG10008), PI3K-p85 (BD Biosciences, Cat # 610045) and Actin (used as loading control; Sigma, 1:7500) were used. All the remaining antibodies were from Cell Signaling Technology and used at 1:1,000 dilution. Nuclear and cytoplasmic lysates were prepared using NE-PER Nuclear and Cytoplasmic Extraction Reagents (Life Technologies, Cat # 78833) per the manufacturer's instructions.

O-glycosylation immunoblots were performed on total protein lysates using O-GlcNAc antibody (mouse, Santa Cruz Biotechnology, cat# sc-59623, 1:1,000). Blots were scanned and immunoreactive bands having molecular weight greater than 50 kDa were quantified using Image J software. For each sample, the intensity of glycosylated immunoreactive band(s) was normalized first to their respective GAPDH. Following this, the median intensity of each normalized band was calculated across multiple biological replicates (*n*=3 for 22Rv1 and *n*=2 for LNCaP-ABL) and data represented as plots in Supplementary Fig. 12E. Original scans of all western blots are shown in Supplementary Figs 16–29.

### Tissue microarray

Tissue microarray was immunostained using GNPNAT1 antibody (Proteintech, 1:500 dilution) and UAP1 antibody (Abcam, 1:500 dilution) and scored by Dr Ittmann, a genito-urinary specialty trained pathologist. All tissues were collected under an institutional review board protocol and with informed consent at BCM and University of Michigan (UMICH).

### BrdU and ChIP assays

BrdU assay (Calbiochem/Millipore, cat # 80002-206) was performed per the manufacturer's instructions. ChIP assays were done following the manufacturer's protocols (EMD Millipore Corporation, cat # 17–10461). Immunoprecipitation was carried out using either 10 μg of rabbit SP1 antibody (Cell Signaling) or using rabbit non-immune IgG (Sigma), as a control. Fold change in enrichment of SP1 occupancy on gene-specific promoters was calculated relative to the promoter binding obtained using non-immune IgG (negative control).

### FluoReporter assay

The assay was performed by quantification of DNA content using fluoreporter assay (Themo Fisher Scientific, Cat # F-2962) as per the manufacturer's protocol and as described previously^42^. Briefly, number of cells seeded in a 96-well plate were optimized for both LNCaP-ABL and 22Rv1 cells with GFPT1 siRNA or control siRNA. For each experiment, empty wells were used as background control. Furthermore, fluorescence was detected at 360 nm (excision) and 460 nm (emission) using a fluorescence microplate reader. The intensity values of blank wells were substrated from experimental data and for each sample (GFPT1 targeted siRNA) data were normalized to control wells (with control siRNA).

### Celigo-based cell counting

Celigo-based counting was used to determine change in cell numbers either after overexpression of GNPNAT1 or to assess the effect of metabolite treatments in CRPC-like cells. For all cell lines used for Celigo-based cell counting assay, optimal cell numbers were determined using parameters suggested by the manufacturer (Nexcelom Bioscience LLC). Approximately 2,000 and 3,000 cells, respectively, for 22Rv1 and LNCaP-ABL were seeded in replicates on day 0 in independent 96-well plates (Greiner 655090). After 24 h of plating, cells were subjected to treatment if any and scanned for their cell numbers at 0, 24, 48, 72 and 96 h post treatment or followed up to 6 days post transfection (GNPNAT1 over expression). At the end of 96 h of culture or 6 days post transfection, cells were visualized with diamidino-2-phenylindole, imaged using a blue filter and counted. For each treatment group, the median cell numbers across all replicates were calculated across the various time points. Following this, the cell numbers in the treatment group were represented as percentage of cells relative to the control group, along with their associated standard deviation. *P*-values to assess significance of the effect were computed using an ANOVA test in R software.

### Luciferase reporter assay

Plasmid containing ChREBP-luciferase was a kind gift from Dr Lawrence Chan, BCM. This was transfected using Lipofectamine 2000 (Life Technologies) into 22Rv1 KD and NT cells. Forty-eight hours later, luciferase signal was measured using a luminometer after adding Steady-Glo Luciferase reagent from Promega Inc. (cat # E2510) per the manufacturer's protocol (data were normalized using total protein content in the cells).

### High throughput microscopy and image analysis

22Rv1 NT, KD1 and KD5 cells were seeded on optical glass-bottom 96-well plates (Greiner Bio-One), fixed and labelled with ChREBP (Novus, cat # NB400-136V, 1:500) antibody following standard immunofluorescence protocols. Plates were imaged using an IC-200 high-throughput microscope (Vala Sciences) with a × 20/0.75 numerical aperture objective. For each sample, a minimum of eight fields per well were imaged. Image analysis was performed for each cell using an automated mIA software platform^43^ and the total averaged intensity was normalized to the respective cell area. The normalized intensity was then used to calculate population marginal mean difference for expression of ChREBP in 22Rv1 NT and KD cells.

### *In vivo* xenografts

All mouse experiments performed at either the BCM or the UMICH were approved following the guidelines of BCM Institutional Animal Care and Use Committee and Unit of Laboratory Animal Medicine- UMICH, respectively. Male SCID-Beige Fox Chase and Nod-SCID Gamma mice (between 6 and 8 weeks of age) were obtained, respectively, from Michael Lewis laboratory at BCM and Charles River laboratory (Frederick, MD). The mice were used to generate xenografts using either 22Rv1 (experiments conducted at both BCM and UMICH) or LNCaP-ABL (study conducted at UMICH) cells that were stably transduced with either control shRNA or shRNA targeting GNPNAT1. To generate the xenografts, subcutaneous injection of 100 μl of a mixture containing cells (50 μl of 3 × 10^6^ 22Rv1 or 1 × 10^6^ LNCaP-ABL in 1 × DPBS) and Matrigel (50 μl, 5 mg ml^−1^ protein concentration; Corning cat #354248) was carried out in the hind-flank area of the mice under isoflurane anaesthesia. Tumours were measured using calipers either twice (LNCaP-ABL) or thrice (22Rv1) per week for minimum of 8 weeks. Tumour volume was calculated using the formula TV=(length × width^2^) × 0.52 (ref. 44). In case of tumours generated using luciferase-labelled 22Rv1 cells, tumour growth was additionally monitored using bioluminescence imaging with a Xenogen IVIS 200 after intraperitoneal D-luciferin (100 μl of 10 μg ml^−1^ solution) injection. Intra-cardiac injections (performed at UMICH) using luciferase-labelled 22Rv1 cells used 2.5 × 10^5^ cells in 100 μl of DPBS. Mice were anaesthetized using isoflurane. Successful injections were assessed by measuring the initial bioluminescence at the tumour locations. Mice with high bioluminescent signal in areas around or in the heart during the first 2 weeks were deemed unsuccessful, and removed from the study. At the end of the study, the tumours were harvested and flash frozen for further analysis. Tumours were observed in all the mice injected with LNCaP-ABL cells (both NT and KD). L-KD5 tumours reached 1,000 mm^3^ by day 53 at which point these animals were killed. Figure 2j shows the relative tumour growth curve across all groups up to this point. L-KD1 and L-NT mice were killed around days 55–87. By the end of the experiment, the median tumour volumes for L-KD1 and L-NT were 908 and 859 mm^3^, respectively. All the molecular analysis was performed on tumours resected at the end of the experiment. In case of mice injected with 22Rv1 cells (Fig. 2i), 7 and 8 mice had tumours in the KD and NT groups, respectively. Mice in which tumours did not form were removed from the study. Mice injected with 22Rv1 labelled with luciferase (Supplementary Fig. 6A–C) had 9 and 10 tumours, respectively, in the NT and KD groups. For the analysis of the data on tumour volume (Fig. 2i,j and Supplementary Fig. 6A), a GLM in the R software was used. Here both the treatment (that is, NT versus KD1 versus KD5 for ABL, and NT versus KD for 22Rv1) and the time of treatment were used as fixed effects. The sample replicates were treated as random effects. Model parameter estimates were obtained using a maximum likelihood method and implemented in the lme4 package in the R statistical language. The corresponding *P*-values of the test statistic computed using GLM for the treatment effects are reported. We have not selected a specific time point to assess significance. Rather, the GLM model uses the rate of tumour growth over the entire time period of treatment to assess the overall significance of the effect.

### Magnetic resonance imaging

MRI was used to confirm the presence of metastasis in jaw and pelvic bones in mice injected intra-cardially using 22Rv1 GNPNAT1 KD and NT control cells. MRI was carried out on a 7T Agilent Direct Drive system (Agilent, Inc.) with a quadrature mouse body volume coil (m2m Imaging Corp.). During all MRI procedures, animals were anaesthetized with a 1–2% isoflurane/air mixture while maintaining body temperature using a heated air system (Air-Therm Heater, World Precision Instruments). Anatomical MR images were acquired by a fast spin echo sequence with the following parameters: repetition time/echo time=4,000/15 ms, field of view=40 × 30, matrix size=128 × 128, slice thickness=0.5 mm, echo train=8, echo spacing=15 ms and number of slices: 25∼30. Mice with tumours detected by bioluminescence imaging were imaged with MRI to confirm the tumour presence. Contrast-enhancement was performed by i.p. administration of 50 μl of 0.5 M gadolinium-DTPA (Magnevist, Bayer Healthcare Pharmaceuticals) 5 min prior to image data acquisition. Delineation of tumour from healthy tissue was determined using a contrast-enhanced T1- or T2-weighted spin-echo images.

### UDP-GlcNAc and mannose treatments *in vivo*

For UDP-GlcNAc and mannose treatments, the metabolites (Sigma-Aldrich) were resuspended in PBS. Intra-tumoral UDP-GlcNAc or mannose injections (50 μl at 60 mM) or PBS alone, were performed twice a week into castrated Nod-SCID Gamma male mice bearing xenograft tumours generated using wild-type LNCaP-ABL cells. The experiments were done in three independent sets. To generate tumours, 5 million cells were injected in set 1 and 10 million cells were used in sets 2 and 3, respectively. Set 1 contained four animals each treated with PBS (vehicle control) and 60 mM UDP-GlcNAc, after the xenografts reached an average volume of 300 mm^3^ (Fig. 4e). Tumours were monitored for 72 days. Set 2 contained seven animals each treated with PBS (vehicle control) and 60 mM UDP-GlcNAc, after the xenografts reached an average volume of 100 mm^3^ (Supplementary Fig. 15A). Tumours were monitored for 38 days. Set 3 contained five and six animals treated with mannose (60 mM, un-related metabolite control) and 60 mM UDP-GlcNAc, respectively, after the xenografts reached an average volume of 100 mm^3^ (Supplementary Fig. 15B). Tumours were monitored for 40 days. Tumour implantation sites were pre-treated with iodine swab stick before an injection. Measurement of tumour volume was carried out every 4 days in first and third set of experiments and every 3 days in the second set of experiments. For each animal, the tumour volume at each time point was normalized to its baseline value obtained before starting the treatment. The percent change in the median tumour volume for all the animals in group was calculated along with the associated median absolute deviation and plotted against time (in days). *P*-value was calculated using GLM model as described earlier. Animals were euthanized once the tumour size reached a volume of 1,000 mm^3^.

### Treatment of CRPC cells using metabolites or enzalutamide

Cell viability and cell numbers post treatment with metabolites or enzalutamide or both were done using MTT assay and Celigo-based cell counting (refer to section marked Celigo-based cell counting, above), respectively. For MTT assay, 10,000 LNCaP-ABL or 22Rv1 cells were plated independently in replicates (total of either 6 or 12 replicates) in 48-well plates and treated with either PBS/dimethylsulphoxide (vehicle) or 10 μM enzalutamide or 60 mM UDP-GlcNAc or a combination of 10 μM enzalutamide and 60 mM UDP-GlcNAc. Ninety-six hours post treatment, cell viability was assessed using MTT assays per the manufacturer's protocols. Data were analysed using an ANOVA model generated with three factors: (i) treatment, (ii) cell line and (iii) experimental replication. Subsequently, differences in the mean level of the treatments for different cell lines were compared controlling the Family Wise Error Rate (for multiple comparisons) at the *α*=0.01 level, using Bonferroni method^45^. ANOVA model was used to compute *P*-values comparing either enzalutamide or combination of enzalutamide and UDP-GlcNAc with other treatments.

## Additional information

**Accession codes:** The gene-expression data for GNPNAT1 KD 22Rv1 and LNCaP-ABL cells have been deposited in the GEO database and can be accessed using the GEO ID GSE67537.

**How to cite this article:** Kaushik, A. K. *et al*. Inhibition of the hexosamine biosynthetic pathway promotes castration-resistant prostate cancer. *Nat. Commun.* 7:11612 doi: 10.1038/ncomms11612 (2016).

## Supplementary Material

Supplementary Information Supplementary Figures 1-29, Supplementary Tables 1-6 and Supplementary Reference.

Supplementary Data 1 This is the matched gene expression and metabolomics data that was used for the integrative analysis. The metabolomics data was originally published in Nature 2009, PMID 19212411.

Supplementary Software R software code used for integrative analysis
