# Supplementary material for: Inhibition of the hexosamine biosynthetic pathway promotes castration-resistant prostate cancer
Source: Nat Commun. 2016 May 19;7:11612. doi: 10.1038/ncomms11612 (PMC4874037; doi:10.1038/ncomms11612)
Supplement: Supplementary Information — Supplementary Figures 1-29, Supplementary Tables 1-6 and Supplementary Reference. [file ncomms11612-s1.pdf]

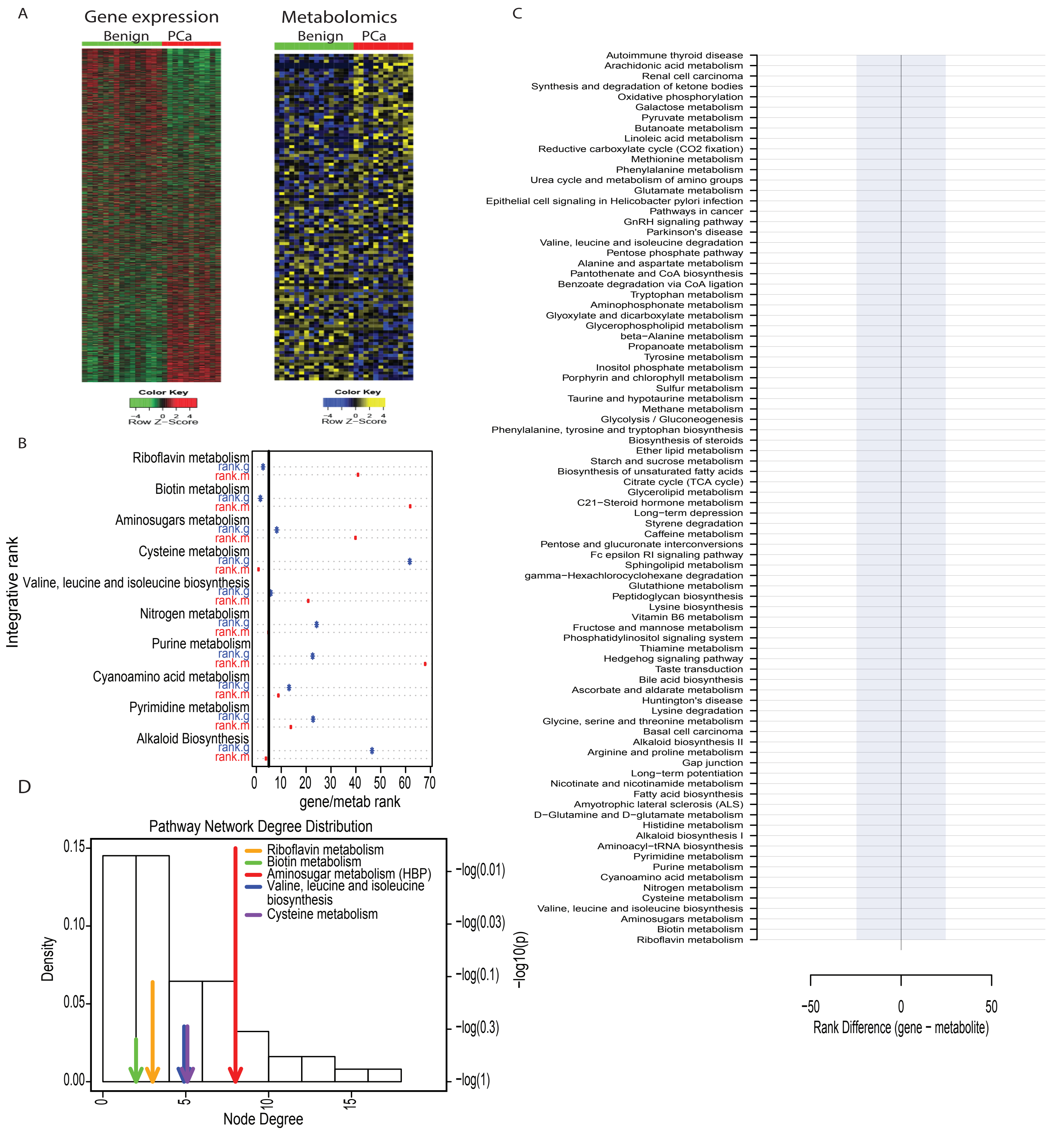

Supplementary Figure 1. Results of integrative analysis carried out using gene-expression and metabolomics data. **A)** Heatmap overview of matched prostate cancer derived gene expression and metabolomic data (Benign=16, PCa=12). **B)** Top pathways independently enriched using gene expression and metabolomic data with associated gene (blue)/metabolite (red) ranks. **C)** Chart representing the concordance between gene-expression and metabolomics data. X axis represents the pathway rank difference derived from gene and metabolite data. Y axis represent common pathways independently enriched using gene and metabolite data. **D)** Graphical representation of overall enrichment of 5 pathways (refer to main text). The length of each arrow correlates with overall enrichment p value. Y axis represents overall density of interaction across biochemical network in KEGG. X axis depicts the total number of interactions for 5 enriched pathways.

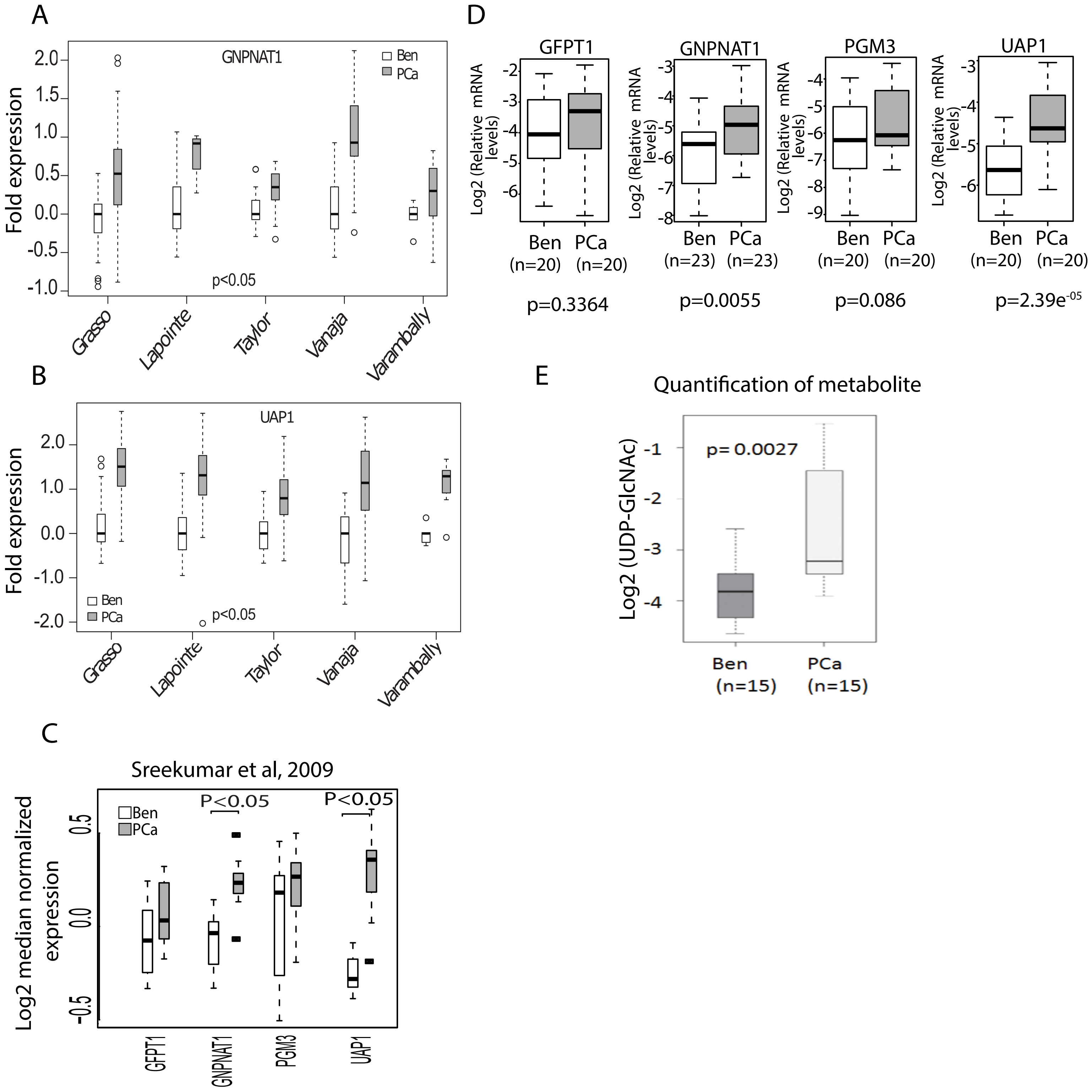

Supplementary Figure 2. Gene-expression profile of HBP genes in localized prostate cancer. **A)** GNPNAT1 was found to be significantly upregulated in 5 different publically available microarray data sets. **B)** Same as in A, but for UAP1. **C)** Both GNPNAT1 and UAP1 were significantly upregulated at mRNA level in localized PCa compared to benign prostate tissue in the gene-expression data used for integrative analysis (Ben =16, PCa=12). **D)** QPCR based analysis of HBP associated genes: GNPNAT1, UAP1, GFPT1and PGM3 in localized prostate cancer and benign tissues. **E)** Mass spectrometry analysis confirmed elevated levels of HBP end product UDP-N-Acetylglucosamine (UDP-GlcNAc) in localized prostate cancer compared to benign tissue (n=15, biological replicates each). For boxplots the horizontal line represents median value while Whiskers represent either <25 or >75 quartile ranges. In all the cases P value <0.05 was considered significant and was computed using student's t-test.

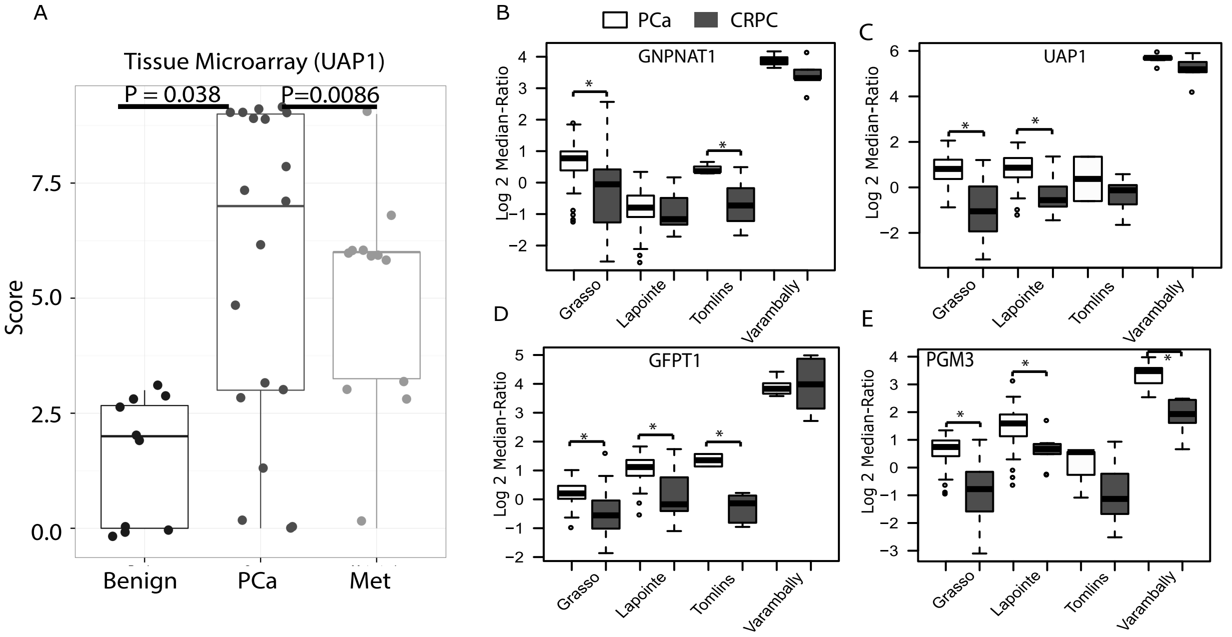

Supplementary Figure 3. Expression profile of HBP genes in metastatic prostate cancer. **A)** Tissue microarray (TMA) analysis reveled significantly elevated expression of UAP1 in PCa (n=19) compared to benign adjacent (n=10). UAP1 is significantly reduced in metastatic tissues (n=12). **B)** GNPNAT1 expression in 4 independently published microarray data sets. Boxplot represent expression in localized tumors (PCa) while solid gray shaded boxplots represent expression in metastatic or CRPC tumor. **C)** Same as in B but for UAP1. **D)** Same as in B but for GFPT1. **E)** Same as in B but for PGM3. For boxplots the horizontal line represents median value while Whiskers represent either <25 or >75 quartile ranges. In all the cases P value <0.05 (*) was considered significant and was computed using Student's t-test.

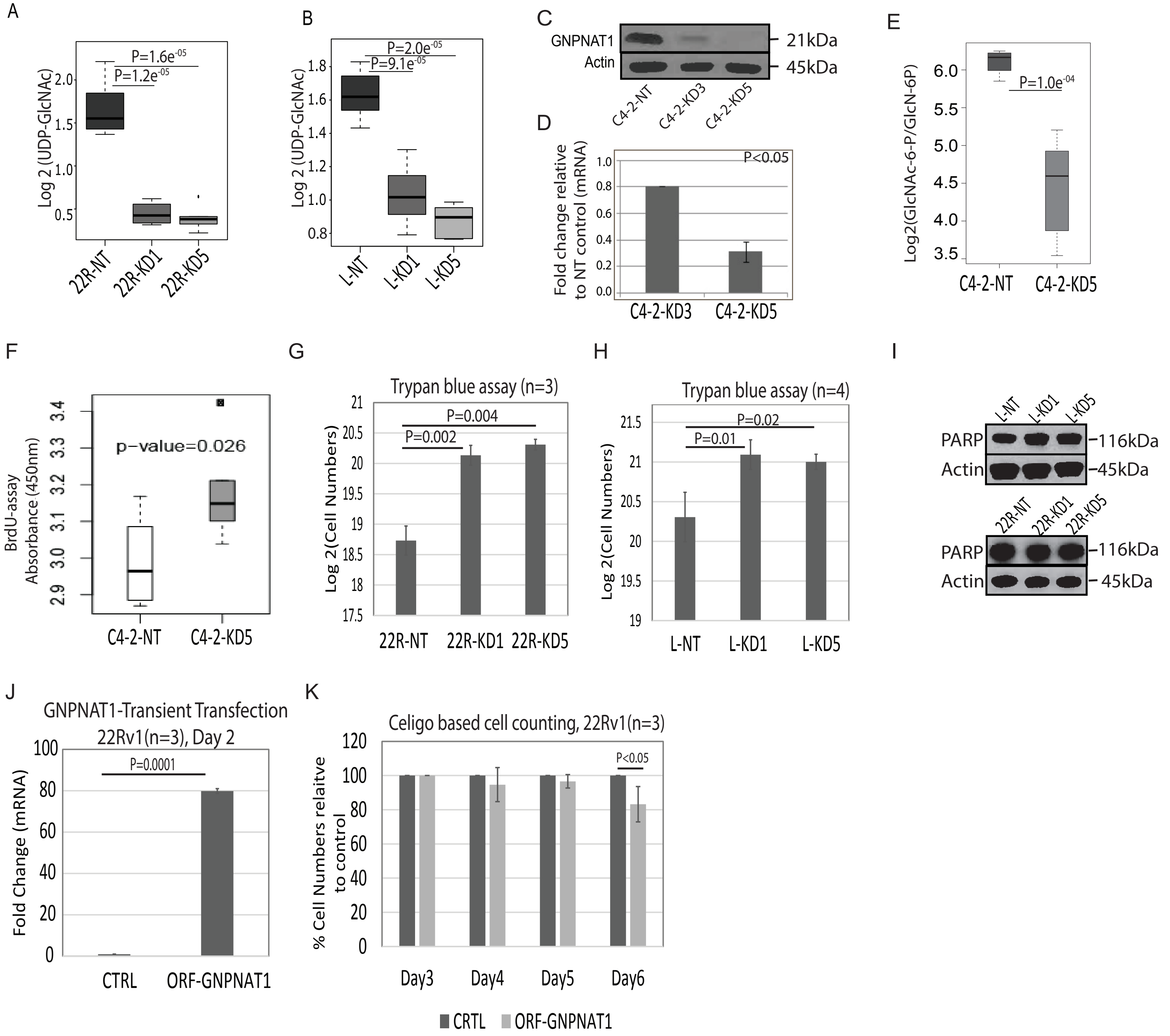

Supplementary Figure 4. Effect of GNPNAT1 knockdown (KD) and over-expression in CRPC-like cells. **A)** Decreased levels of UDP-GlcNAc in GNPNAT1 KD 22Rv1 cells compared to control (n=5, biological replicates). **B)** Same as in A but for LNCaP-ABL cells **(**n=5, biological replicates) **C)** shRNA KD of GNPNAT1 in C4-2 cells leads to loss of GNPNAT1 protein expression (n=3, representative blot), **D)** significant decrease in GNPNAT1 mRNA (n=3, biological replicates) and **E)** significant decrease in product:substrate ratio for GNPNAT1 (n=5, biological replicates). **F)** KD of GNPNAT1 in C4-2 cells (C4-2-KD5) significantly increased its proliferation (n=3, biological replicates, 10 technical replicates/biological replicate). **G)** GNPNAT1 KD in 22Rv1 cells resulted in increased cell numbers when measured using a trypan blue assay (n=3, biological replicates). **H)** Same as in H but for LNCaP-ABL cells (n=3, biological replicates). **I)** Immunoblots showing protein levels of PARP in GNPNAT1 KD LNCaP-ABL and 22Rv1 cells compared to NT controls (n=2: LNCaP-ABL, n=2: 22Rv1; representative blot). **J)** Over-expression of GNPNAT1 in 22Rv1 cells significantly increases its mRNA level assessed by QPCR (n=3, biological replicates). **K)** Over-expression of GNPNAT1 in 22Rv1 cells results in significant increase in cell numbers 6 days post transfection. For boxplots the horizontal line represents median value while Whiskers represent either <25 or >75 quartile ranges. All bar plots are represented in median ± s.d. P values were calcualted using either Student's t-test or ANOVA.

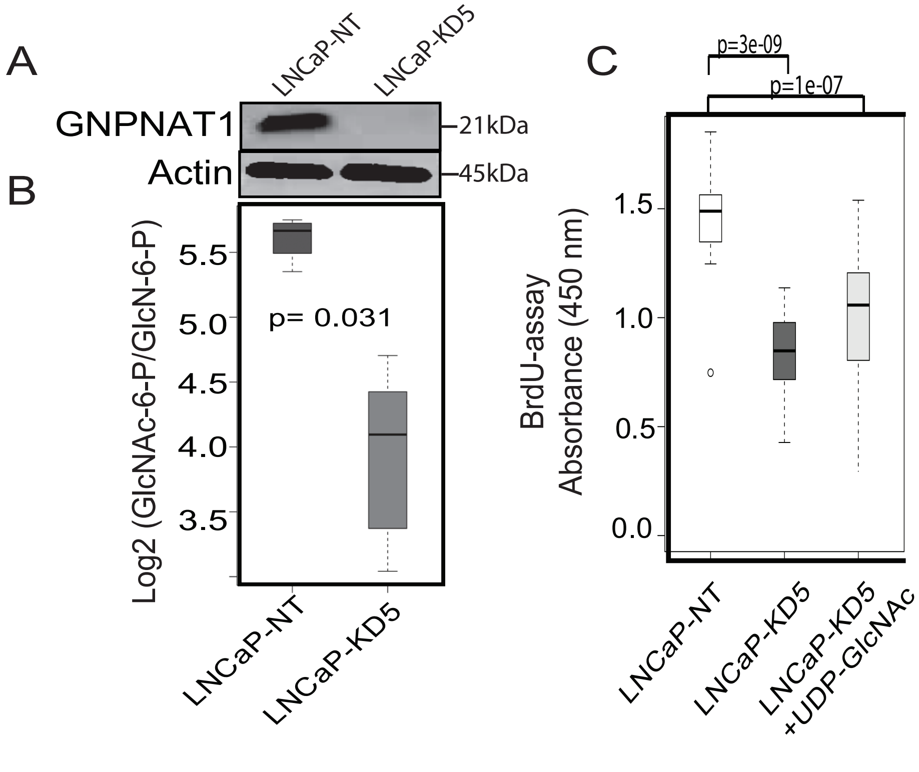

Supplementary Figure 5. Stable KD of GNPNAT1 in androgen dependent (AD) PCa LNCaP cells results in **A)**loss of GNPNAT1 protein expression (n=3, representative blot). **B)** Significant decrease in product:substrate ratio for GNPNAT1(n=3 biological replicate) and **C)** significant decrease in proliferation in KD cells (n=3 biological replicates and 12 technical replicates) which is partially rescued by treating the cells with 15mM of UDP-GlcNAc (LNCaP-KD5 +UDP GlcNAc). For boxplots the horizontal line represents median value while Whiskers represent either <25 or >75 quartile ranges. P values were calcualted using Student's t-test.

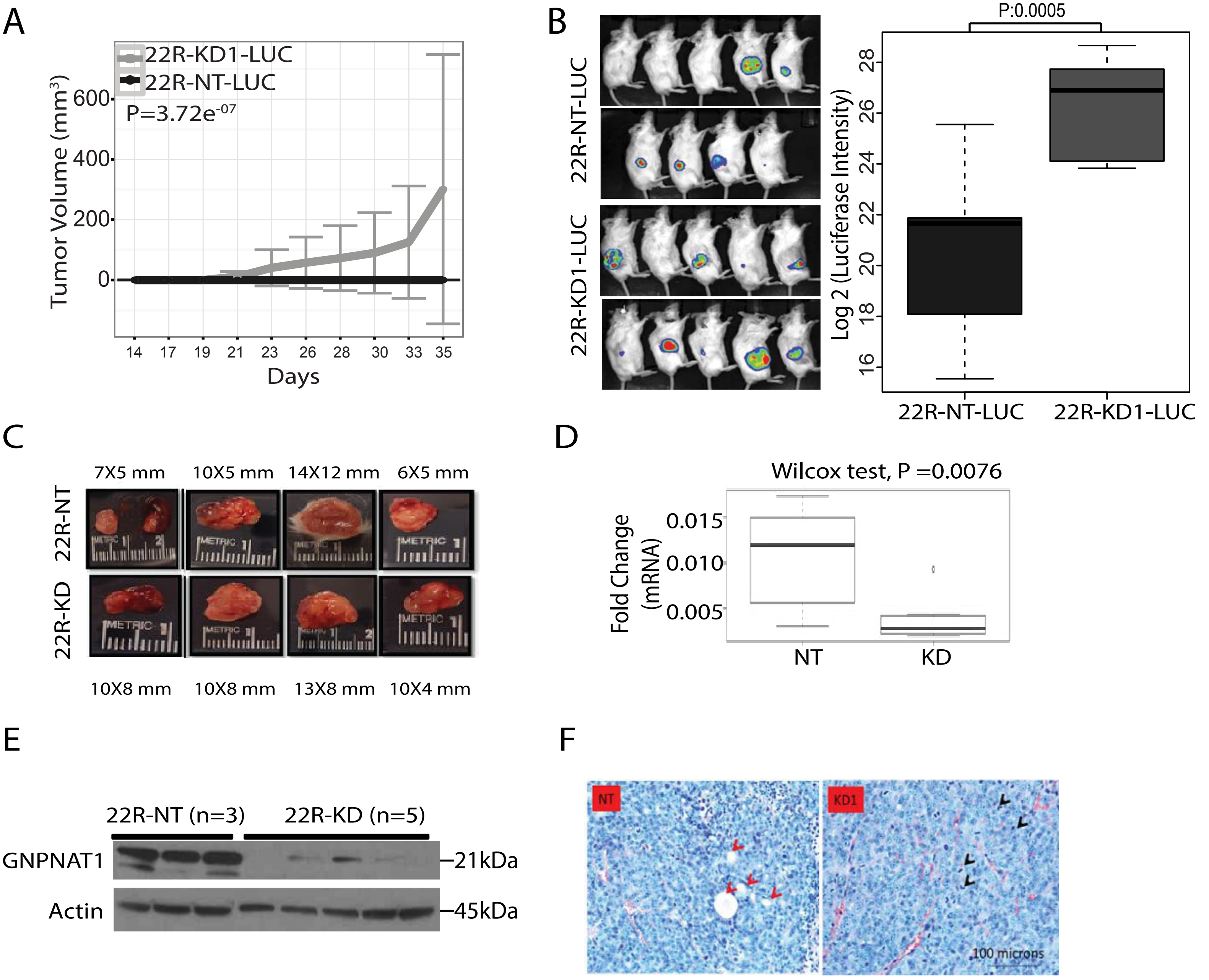

Supplementary Figure 6. Analysis of xenograft tumors generated using GNPNAT1 KD 22Rv1 cells. **A)** Xenograft tumors generated in NOD-SCID male mice using GNPNAT1 KD 22Rv1 (n=10) cells expressing luciferase show significantly higher tumor growth during experimental period as compared to corresponding NT controls (n=9). Significance was assessed using either GLM model **B)** Left panel: Luminescene images of xenograft tumors in mice bearing 22R-KD1-LUC and 22R-NT-LUC cells. Right panel: 22RR-KD1-LUC (n=10) tumors were significantly bigger than their 22R-NT-LUC (n=9) counterparts at the time of euthanasia. Average intensity of luciferase signal was log 2 transformed and the significance was assessed using Student's t test. **C)** Images and morphometric measurement of tumors from 22Rv1 KD1 and NT mice. Numbers indicate the diameter of the tumors in mm (n=4, each group).
D) Representative Q-PCR data of KD and NT-derived tumors for GNPNAT1. GAPDH was used as the house keeping gene (For NT and KD1, 5 and 9 biological replicates were used, respectively). Significance was assessed using Wilcoxon signed-rank test. **E)** same as in D, but for GNPNAT1 protein levels. Actin was used to control for protein loading. **F)** Representative histological section of NT tumors showing lumen (red arrow heads) and in KD tumor showing mitotic figures (black arrow heads). For boxplots the horizontal line represents median value while Whiskers represent either <25 or >75 quartile ranges.

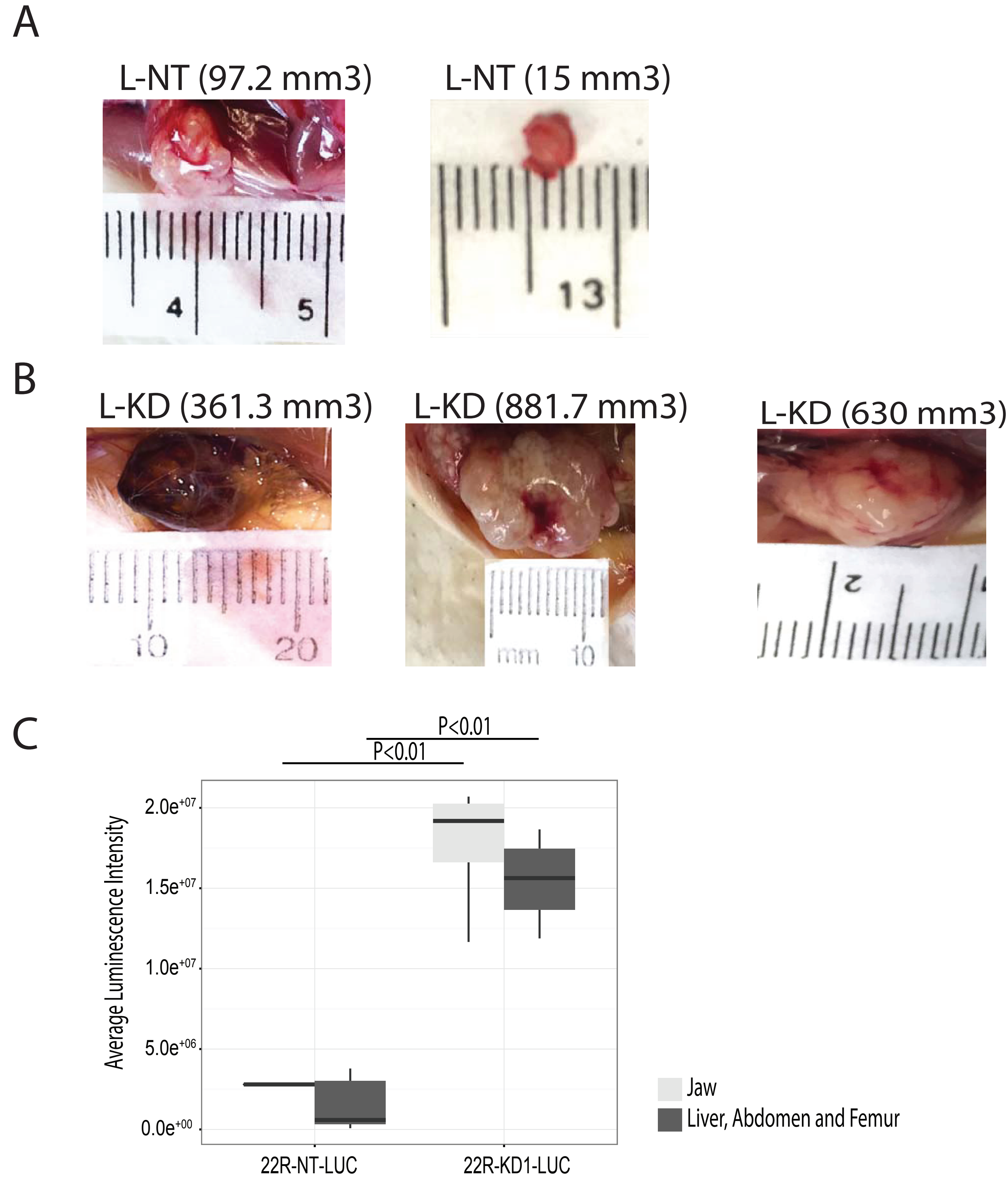

Supplementary Figure 7. Morphometric analysis of metastatic LNCaP-ABL tumors. **A)** Morphometric measurement of metastatic tumors in the lymph nodes in LNCaP-ABL NT (L-NT) mice. **B)** Morphometric measurement of metastatic tumors in the lymph nodes in LNCaP-ABL KD (L-KD) mice. 5/6 mice (2 NT and 3 KD) that were followed after resection of primary tumor for 104 days showed metastatic lesions. The volume of these lesions are in parentheses. Notably L-KD mice formed significantly (P<0.038) bigger lesions compared to L-NT mice. P value was computed using student's t test. **C**) 22R-KD1-LUC cells when injected intracardiac into male castrated NOD SCID gamma mice formed significantly more metastatic lesions in the abdomen, brain, jaw and pelvic bones compared to 22R-NT-LUC cells (refer Fig. 2K) (For NT, n=4 and for KD n=5). For boxplots the horizontal line represents median value while Whiskers represent either <25 or >75 quartile ranges. P value for average luminescence intensity of the luciferase signal was assessed using student's t test.

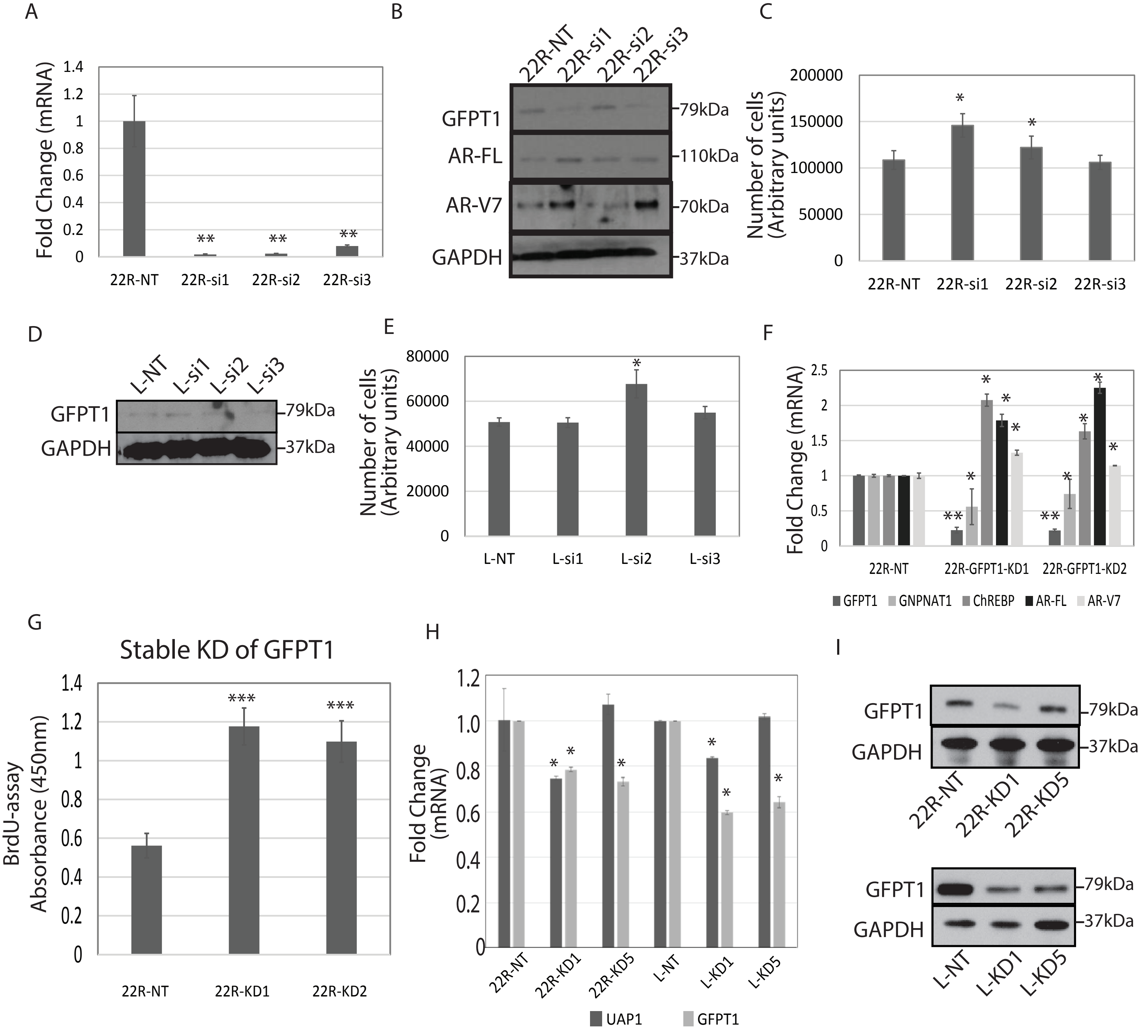

Supplementary Figure 8. Knockdown of GFPT1 increases cell proliferation in CRPC-like cells. **A)** QPCR showing decreased GFPT1 mRNA in 22Rv1 cells tranfected with siRNA targeting GFPT1 compared to scambled control (n=3). **B)** Protein levels for GFPT1, AR-FL and AR-V7 assessed using immunoblot analysis in GFPT1 KD 22 Rv1 cells and NT controls. GAPDH is used as loading control. **C)** Proliferation of 22Rv1 upon GFPT1 KD using two independent siRNA (22R-si1, 22R-si2) is significantly elevated compared to NT control, as measured by fluoReporter assay (n=3). **D)** same as in B, but for LNCaP-ABL cells. **E)** same as in C, but for LNCaP-ABL cells. Significant increase in proliferation was seen in L-si2compared to NT control (n=3). **F)** Stable KD of GFPT1 using two independent shRNA's in 22Rv1 cells. In addition, the QPCR also shows significantly elevated mRNA levels for AR, AR-V7 and ChREBP in these cells (n=3). **G)** BrdU based cell proliferation assay shows singificantly increased proliferation in 22Rv1 cells containing stable KD of GFPT1 (22R-KD1, 22R-KD2) comapred to control cells (22R-NT) (n=3). **H)** QPCR showing mRNA levels for GFPT1 and UAP1 in 22Rv1 and LNCaP-ABL cells containing GNPNAT1 KD (n=3). Importantly, mRNA levels of GFPT1 are down regulated in both KD clones in 22Rv1 and LNCaP-ABL. However, mRNA levels of UAP1 are reduced in only one of the KD clones in 22Rv1 and LNCaP-ABL cells. **I)** Decreased protein levels for GFPT1 in GNPNAT1 KD 22Rv1 and LNCaP-ABL cells assessed using immunoblot (representative blots, n=2 each). Pvalues: *<0.05, **<0.01, ***<0.005. All bar plots are represented in median ± s.d. P values were calcualted using either Student's t-test or ANOVA.

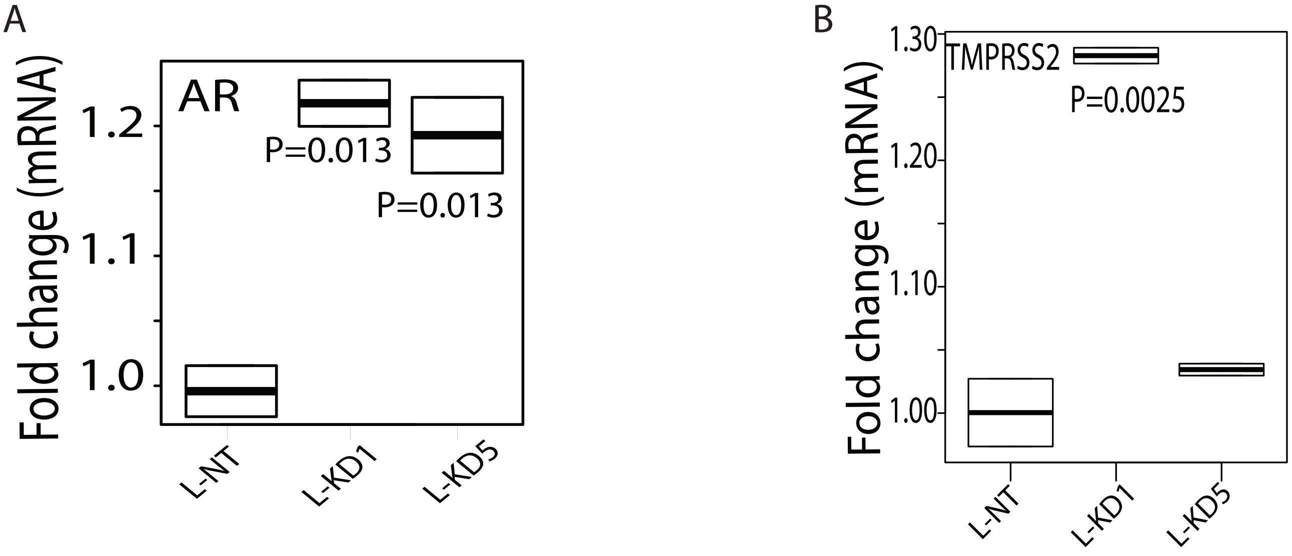

Supplementary Figure 9. Molecular changes associated with GNPNAT1 KD in LNCaP-ABL cells. **A)** Q-PCR showing increased mRNA levels of Androgen Receptor (AR) in LNCaP-ABL KD compared to NT controls (n=3) **B)** same as in A, but for AR-regulated gene TMPRSS2 (n=3). For boxplots the horizontal line represents median value while Whiskers represent either <25 or >75 quartile ranges. P values were calculated using Student's t-test.

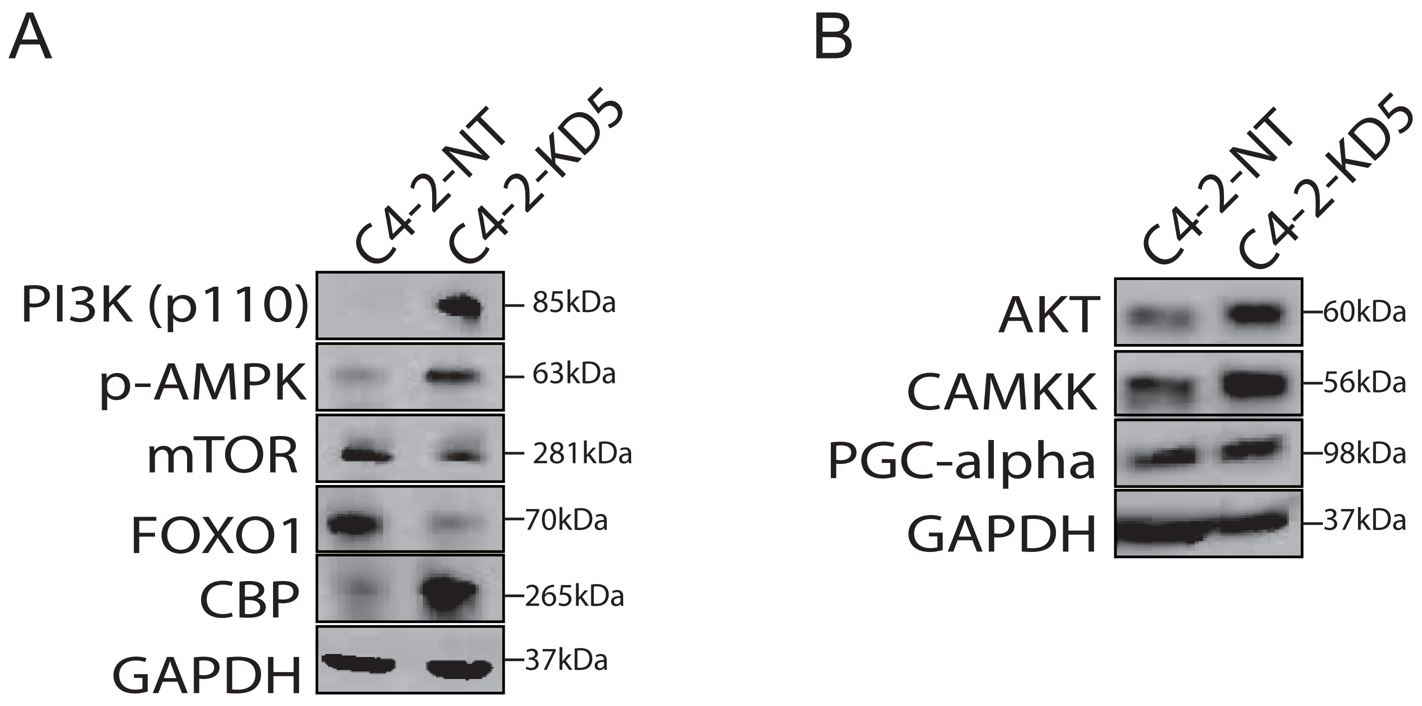

Supplementary Figure 10. **A)** and **B)** Immunoblots showing altered expression of PI3K-AKT pathway in C4-2 cells containing GNPNAT1 KD compared to NT controls.

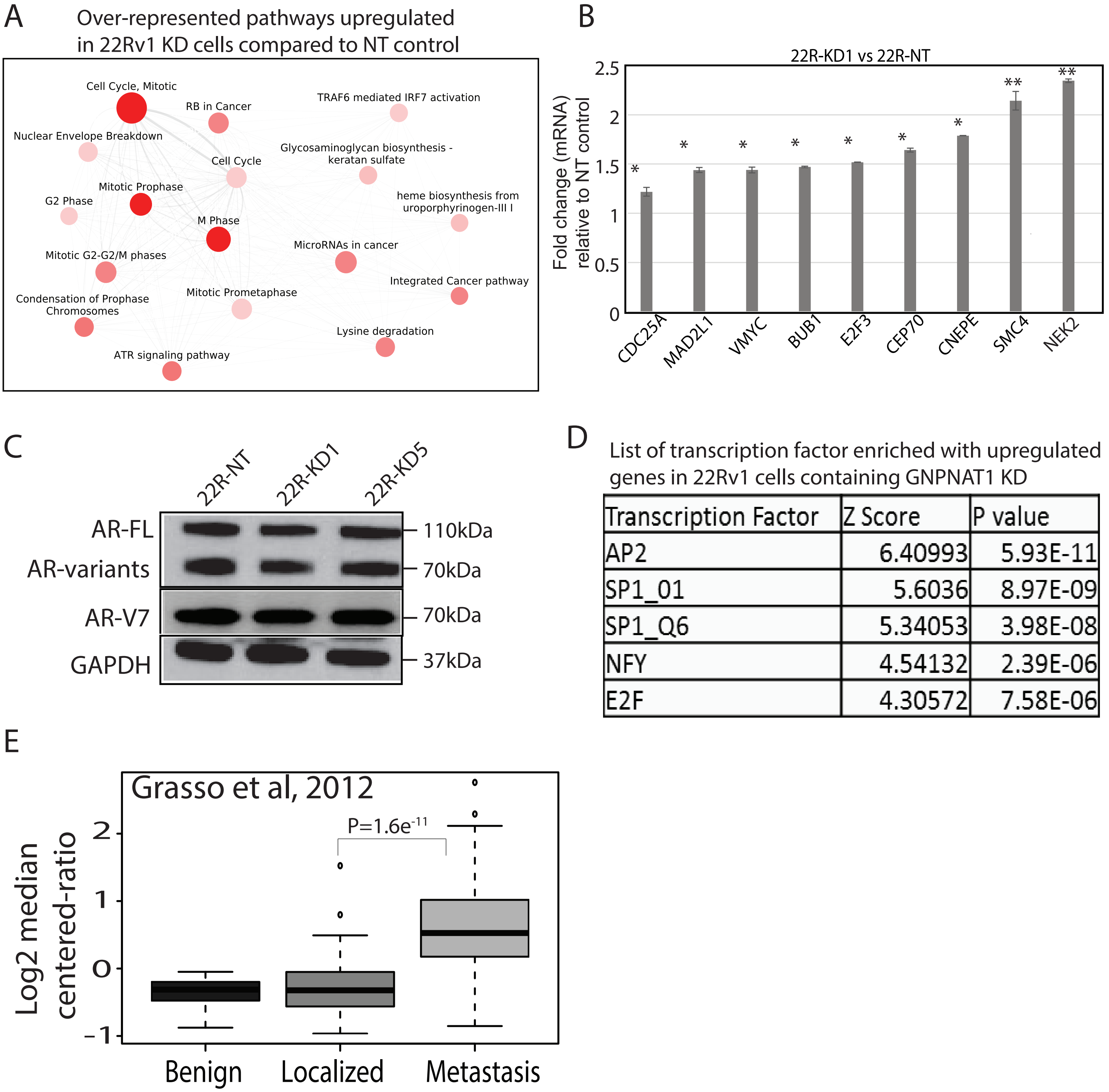

Supplementary Figure 11. Molecular changes associated with GNPNAT1 KD 22Rv1 cells as compared to NT control cells. **A)** Network showing enriched pathway derived using set of up-regulated genes in 22Rv1 cells containing GNPNAT1 KD compared to NT controls. Enrichment analysis was done using Consensus Pathway Database (CPDB) ($P<0.05$). **B)** Q-PCR validation of a subset of cell cylce associated genes in 22Rv1 cells containing GNPNAT1 KD compared to NT control (n=3). **C)** Immunoblot showing protein levels for full length AR and AR-variants (AR antibody (441) from Santa Cruz Biotech., cat# sc-7305) and AR-V7 (Precision antibody, cat# AG10008) in 22Rv1 cells containing GNPNAT1 KD and NT controls. GAPDH was used to control for protein loading (n=3). **D)** Results of transcription factor enrichment analysis in 22Rv1 cells containing GNPNAT1 KD. Z-score defines the extent of enrichment. P-values are reported for each of the enrichments. **E)** mRNA expression profile for SP1 in benign prostate tissue, localized prostate cancer and metastatic disease in Grasso dataset. (P value: *<0.05, **<0.01). All bar plots are represented in median ± s.d.

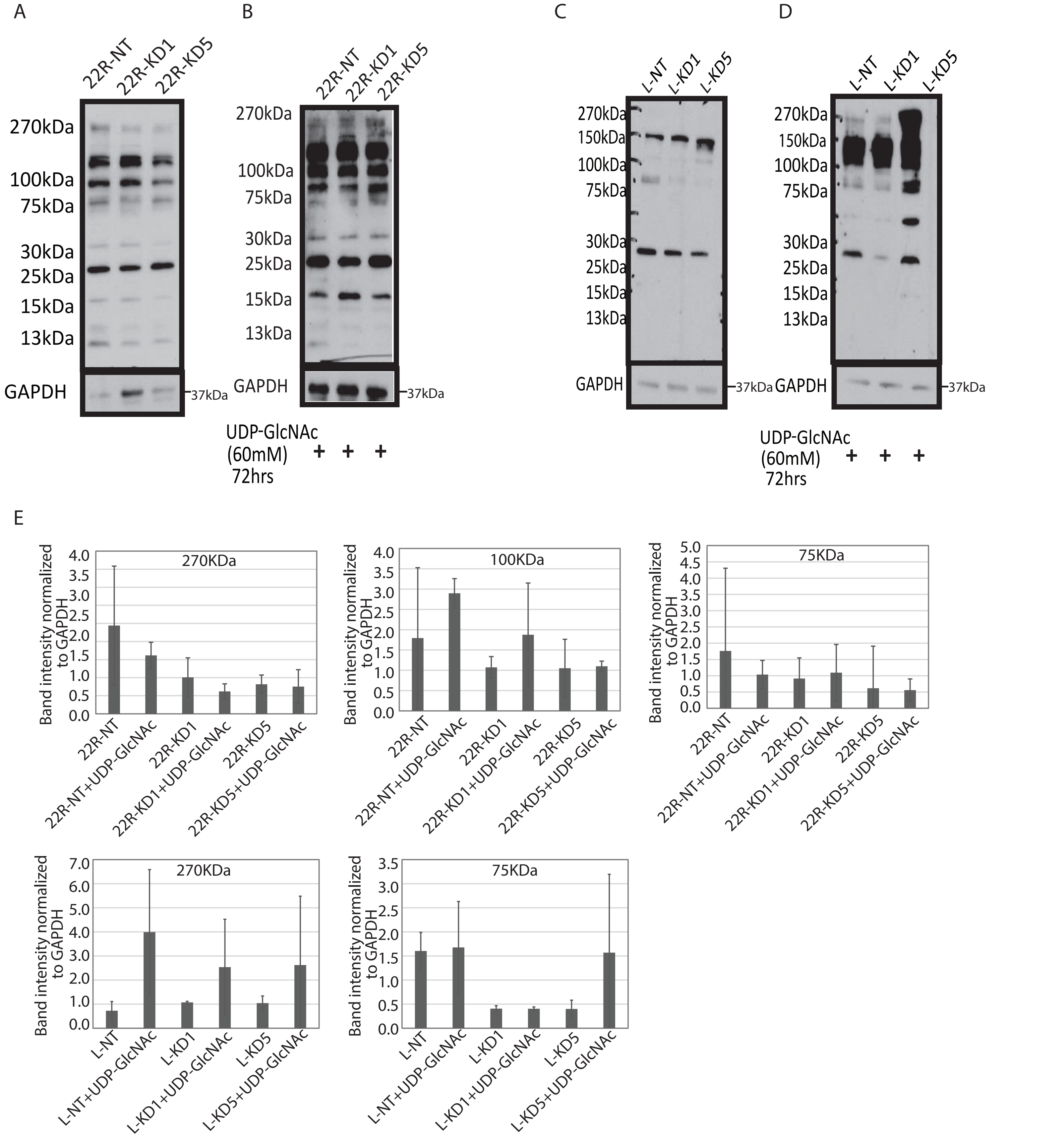

Supplementary Figure 12. Immunoblot showing the overall O-Glycosylation profile and quantification of select glycosylated proteins of molecular weight greater than 50 KDa in 22v1 and LNCaP-ABL containing GNPNAT1 KD, treated with or without 60 mM UDP-GlcNAc for 72 h. **A)** O-Glycosylation profile of GNPNAT1 KD 22Rv1 cells (n=3, representative blot shown). **B)** Same as A, but treated with UDP-GlcNAc (60mM) for 72 hours (n=3, representative blot shown). **C)** O-Glycosylation profile of GNPNAT1 KD LNCaP-ABL cells (n=2), representative blot shown). **D)** same as in C, but treated with UDP-GlcNAc (60mM) for 72 hours (n=2, representative blot shown). **E)** Quantification of selected protein bands of molecular weight greater than 50 KDa in A-D. GAPDH normalized median band intensity is shown in the plots. All bar plots are represented in median ± s.d.

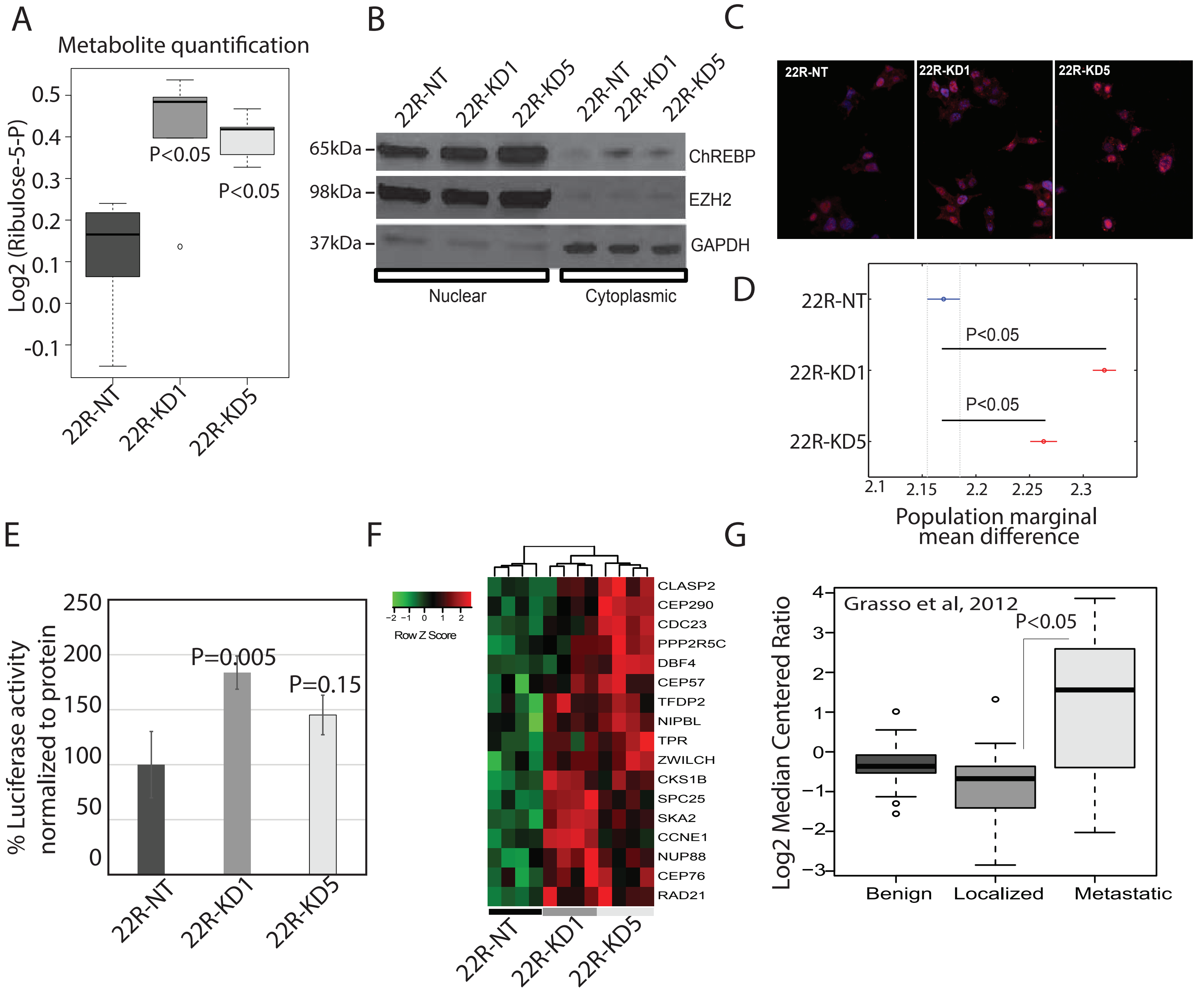

Supplementary Figure 13. **A**) Relative levels of ribulose-5-phosphate measured using Multiple Reaction Monitoring in KD and NT 22Rv1 cells (n=3, biological replciates). **B**) Immunoblot showing the expression of ChREBP in the nuclear and cytoplasmic compartments in 22R-KD and 22R-NT cells. EZH2 and GAPDH were used to examine enrichment of nuclear and cytoplasmic fractions respectively. **C**) Representative image of immunoflourescence staining of ChREBP (red) in 22R-KD and NT cells. DAPI (Blue) used for staining cell nucleus. **D**) Quantification of ChREBP staining intensity normalized to cell area in 22R-KD1/5 and NT cells (n=8 technical replicates). The data were generated using high throughput method published previously (doi: 10.1089/adt.2013.532). **E**) ChREBP activity in 22Rv1 cells containing KD of GNPNAT1 and NT. ChREBP-luciferase was transfected into 22Rv1 NT and KD cells and the luciferase activity was measured using a luminometer 48 hrs post-transfection (n=3). Data was normalized to the total protein content. **F**) Heat map showing higher mRNA expression of ChREBP regulated genes in 22R-KD cells compared to NT controls (n=4, biological replicates each). List of ChREBP genes were obtained from the ChiP-Seq data published recently (doi:10.1210/en.2014-1666). **G**) mRNA expression profile for ChREBP in prostate cancer progression derived from the Grasso et al, 2012 dataset. For boxplots the horizontal line represents median value while Whiskers represent either <25 or >75 quartile ranges. All bar plots are represented in median ± s.d. P values were calculated using either Student's t-test or ANOVA.

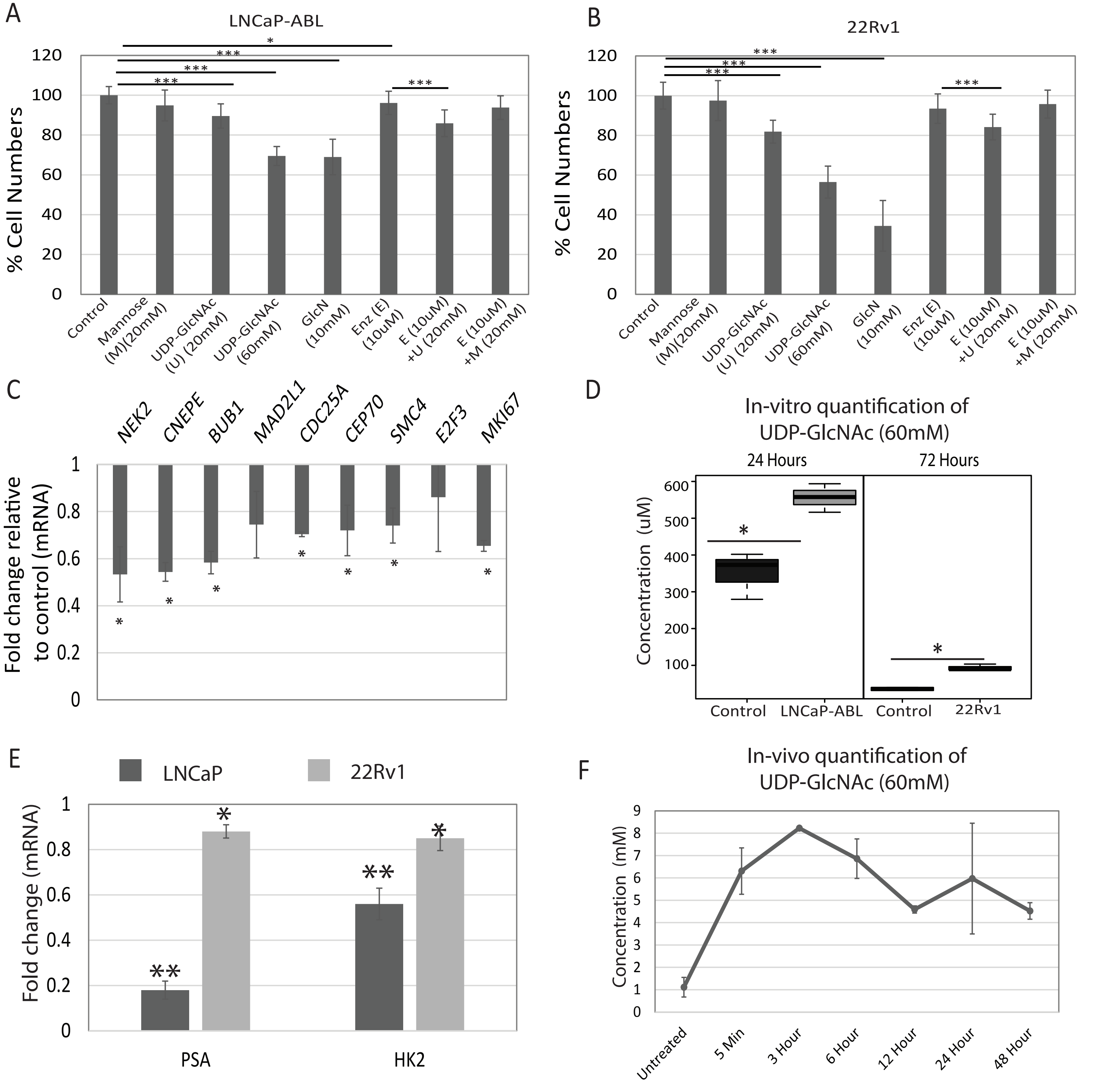

Supplementary Figure 14. In-vitro treatment of CRPC with UDP-GlcNAc. **A)** Percent live LNCaP-ABL cells with no treatment or after treatment for 96h with indicated concentrations of Mannose (non-specific metabolite, 20 mM), UDP-GlcNAc (20 and 60 mM), GlcN (10 mM), enzalutamide (10 uM) and combination of enzalutamide (10 uM) with either UDP-GlcNAc (20 mM) or Mannose (20 mM). Data was obtained using cell counting software in the Celigo instrument (n=4). **B)** Same as in C, but for 22Rv1 cells (n=4). **C)** Treatment of 22Rv1 cells with UDP-GlcNAc (60mM) for 96 hours (n=3) significantly decreased the expression of a subset of cell cycle genes that were elevated in 22Rv1 cells containing GNPNAT1 KD (refer Supplementary Fig. 11 B). **D)** Quantification of internalized UDP-GlcNAc using LC-MS after treatment of LNCaP-ABL (n=3) and 22Rv1 (n=3) cells with the metabolite for 24 hours and 72 hours, respectively. **E)** mRNA fold change showing decrease in the expression of PSA and HK2 in 22Rv1 cells when treated with enzalutamide (n=3). LNCaP cells were used as a positive control for the effect of enzalutamide on the expression of PSA and HK2**. F)** Same as in D, but in xenograft tumors. Each mice bearing 22Rv1 xenograft tumor was given intra-tumoral injection of UDP-GlcNAc (60 mM, 50 ul). At different time points as indicated in the X-axis, tumors were harvested from one mouse and analyzed for UPD-GlcNAc levels in technical triplicates. Level of the metabolite was elevated (~ 7 fold compared to baseline) upto 6 h post-injection following which it remained ~ 4 fold higher than baseline (~1 mM) until 48 hours. For boxplots the horizontal line represents median value while Whiskers represent either <25 or >75 quartile ranges. All bar plots are represented in median ± s.d. P values were calcualted using either Student's t-test or ANOVA.
P value: *<0.05, **<0.01, ***<0.005.

A

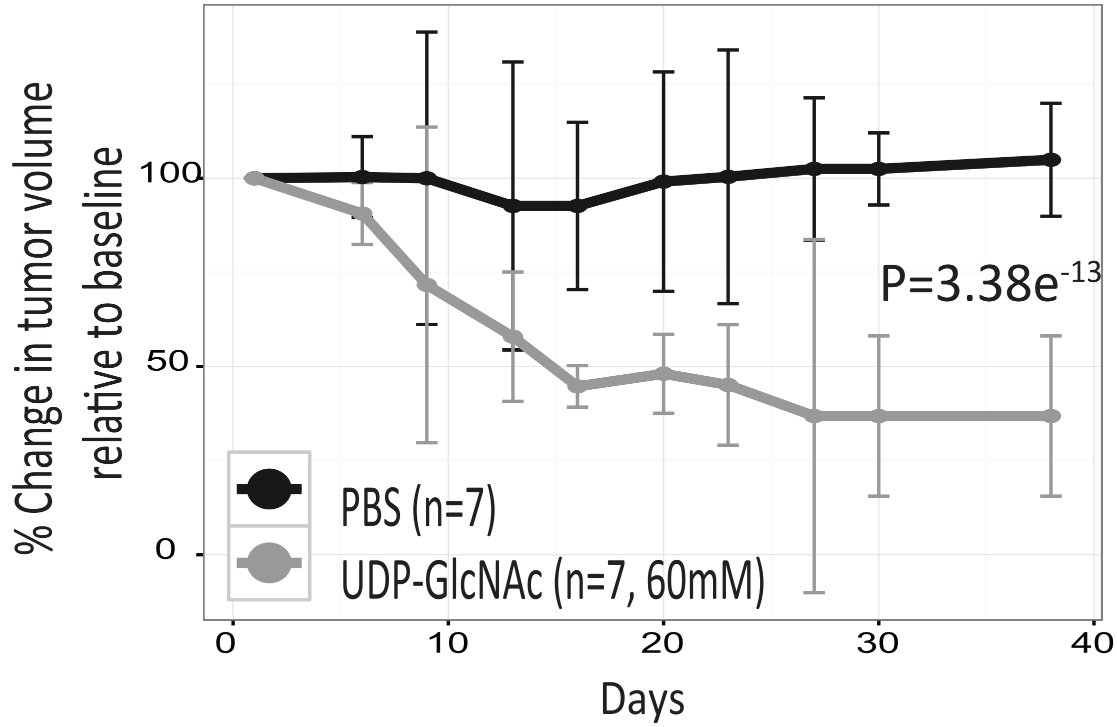

B

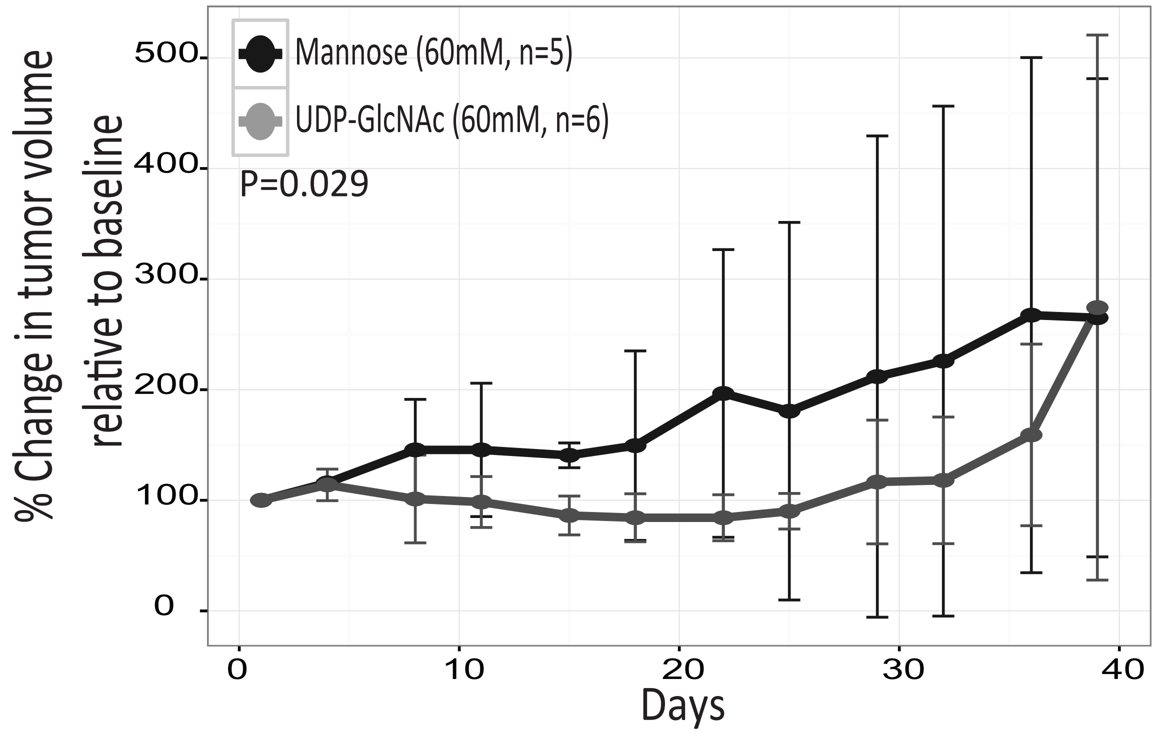

Supplementary Figure 15. in vivo treatment of CRPC with UDP-GlcNAc. **A)** Rate of growth of LNCAP-ABL tumor in the 2nd set of mice that received bi-weekly intra-tumoral injection of either PBS or UDP-GlcNAc (60mM, n=7 each). Tumors treated with UDP-GlcNAc exhibited a significantly reduced gorwth rate compared to those treated with PBS. Treatments with UDP-GlcNAc (60mM) were started when tumor volumes reached an average size of 100 mm^3. **B)** Comparing effect of UDP-GlcNAc (60mM) (n=6) and Mannose (60mM) (n=5) on LNCAP-ABL tumors. Treatments were started when tumors reached 100 mm^3 in volume. Tumors treated with UDP-GlcNAc but not Mannose showed significant reduction in growth rate. P values were calculated using GLM model (refer methods Section in the main text for details).

Supplementary Figure 16: Original scans of the immunoblots used in Figure 2, confirming the knockdown of GNPNAT1 in LNCaP-ABL (KD1, KD5) and 22Rv1 cells (KD1, KD5) compared to respective controls (NT).
Actin was used as loading control. Boxed area highlights the bands that were included in the main Figure 2.

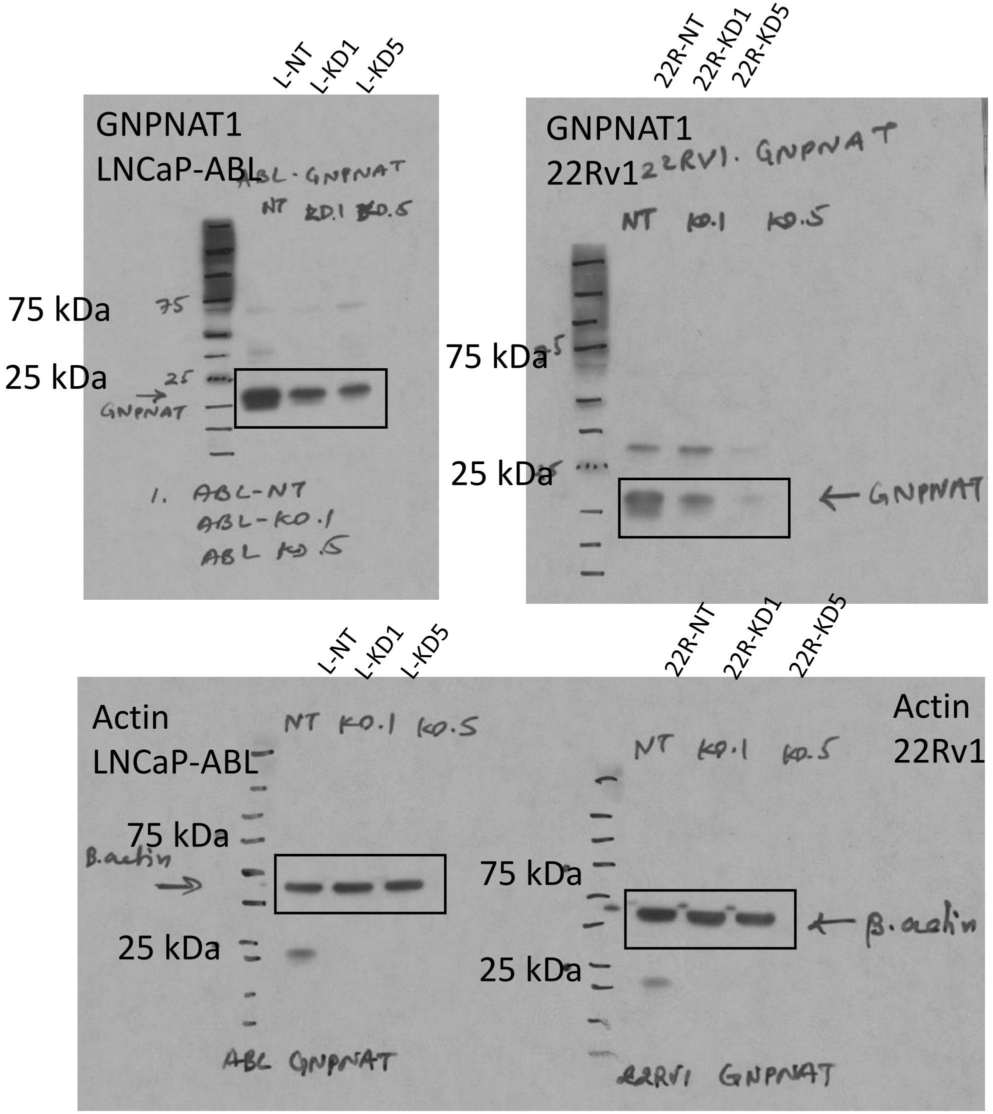

Supplementary Figure 17: Original scans of immunoblots used in Figures 3 B, D showing altered levels of proteins associated with PI3K-AKT signaling axis and AR in GNPNAT1 KD LNCaP-ABL cells compared to control. Actin and GAPDH were used as loading controls for PI3K-AKT and AR immunoblots, respectively. Boxed area highlights the bands that were included in the main Figures 3 B, D.

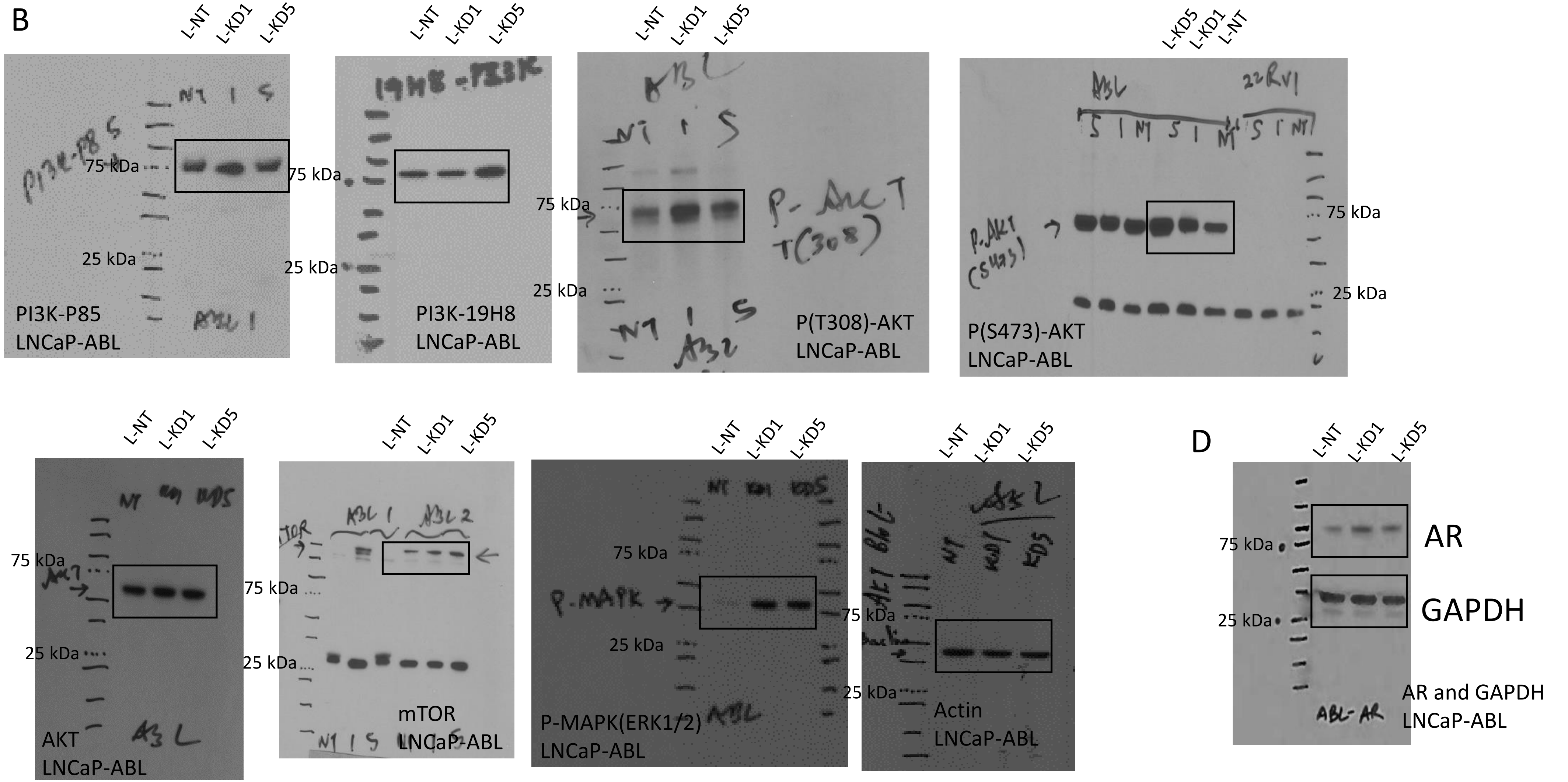

Supplementary Figure 18: Original scans of the immunoblots used in Figures 3 I, L showing elevated levels of SP1 and ChREBP in 22Rv1 cells with GNPNAT1 KD compared to control. Actin was used as control for loading. Boxed area highlights the bands that were included in the main Figures 3 I, L.

I

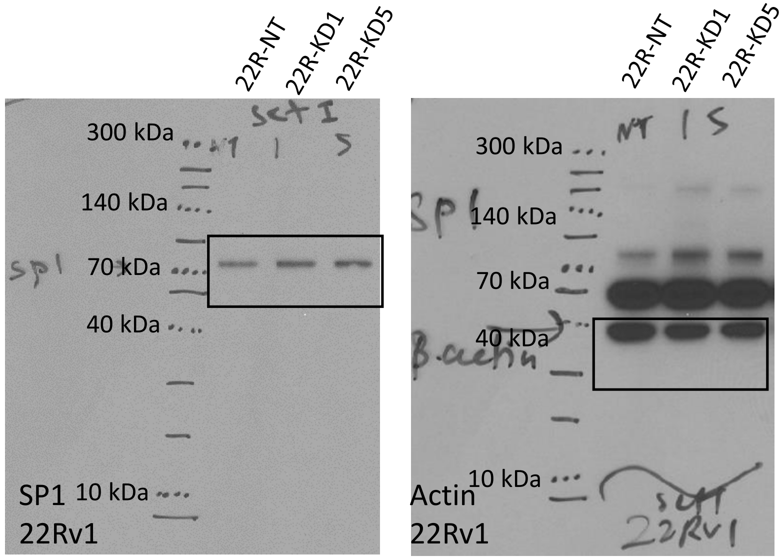

L

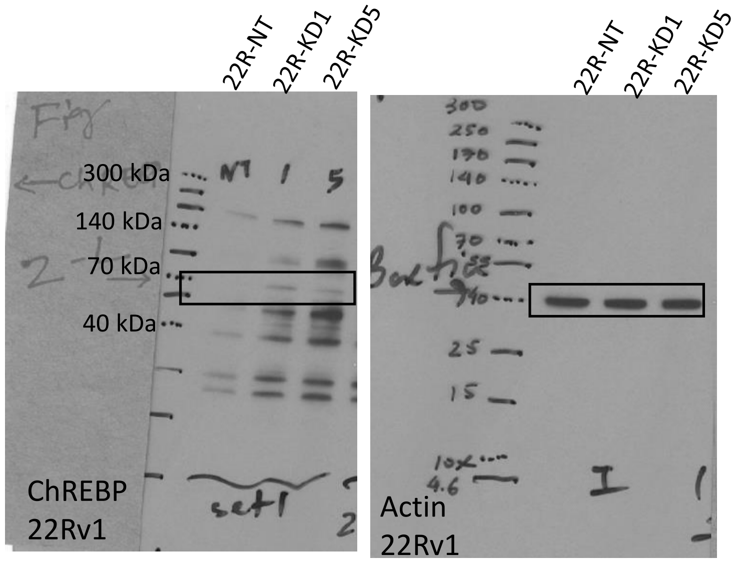

Supplementary Figure 19: Original scans of the immunoblots used in Supplementary Figures 4 C and 4 I showing the knockdown of GNPNAT1 protein in C4-2 cells and levels of PARP in GNPNAT1 KD LNCaP-ABL and 22Rv1 cells compared to control, respectively. Actin was used as control for loading. Boxed area highlights the bands that were included in the main Supplementary Figures 4C and 4I.

C.

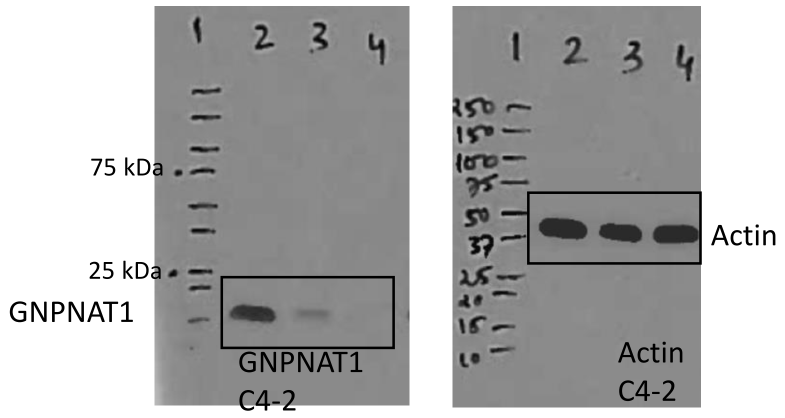

1: Marker
2: C4-2-NT
3: C4-2-KD3
4: C4-2-KD5

I.

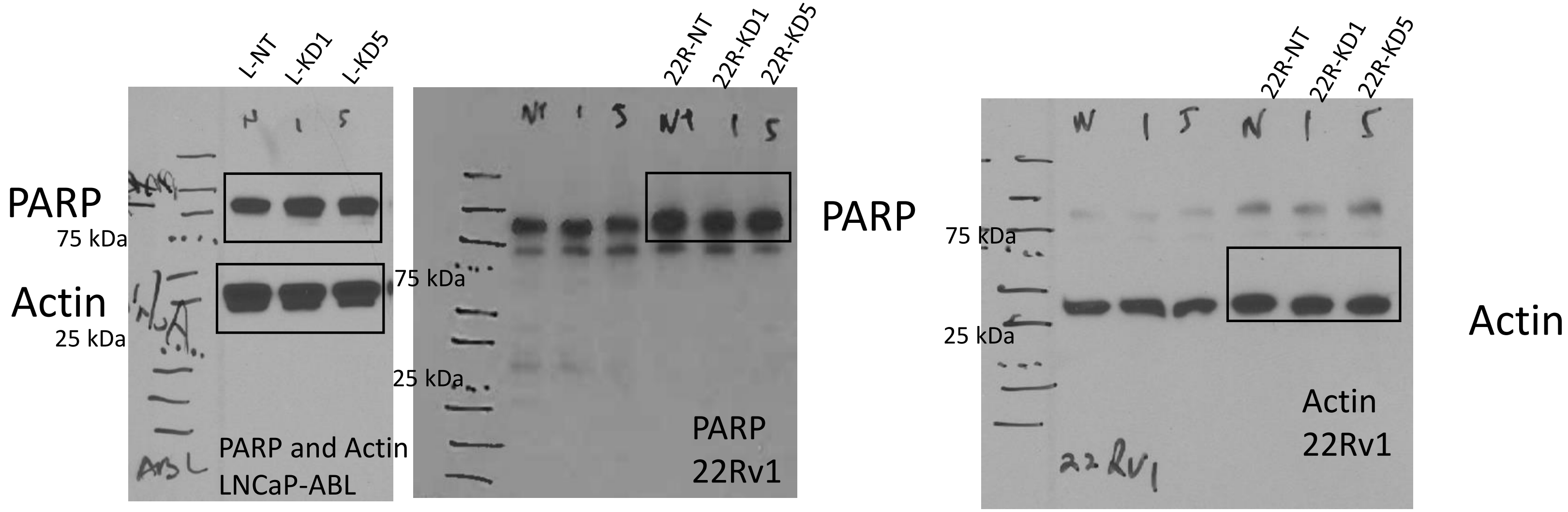

Supplementary Figure 20: Original scans of the immunoblots used in Supplementary Figure 5A confirming the knockdown of GNPNAT1 in LNCaP (KD5) cells compared to control (NT). Actin was used as control for loading. Boxed area highlights the bands that were included in the main Supplementary Figure 5A.

A

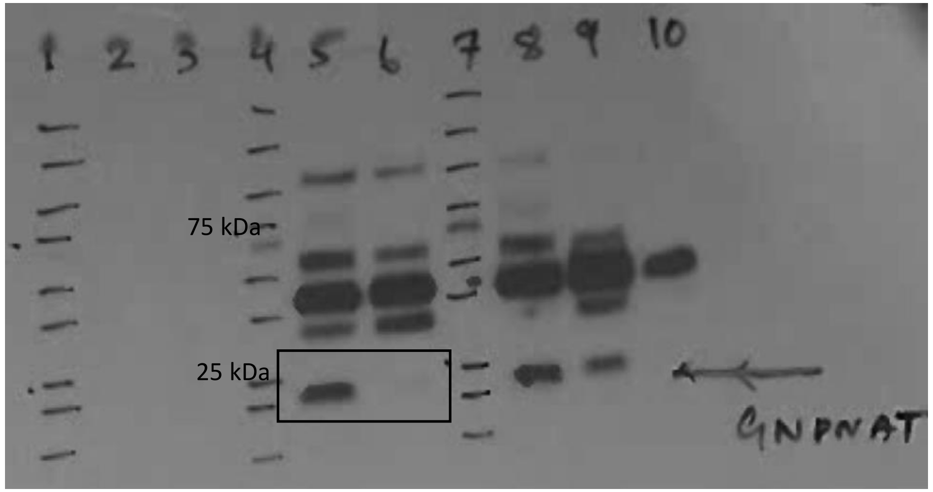

4: Marker
5: LNCaP-NT
6: LNCaP-KD5

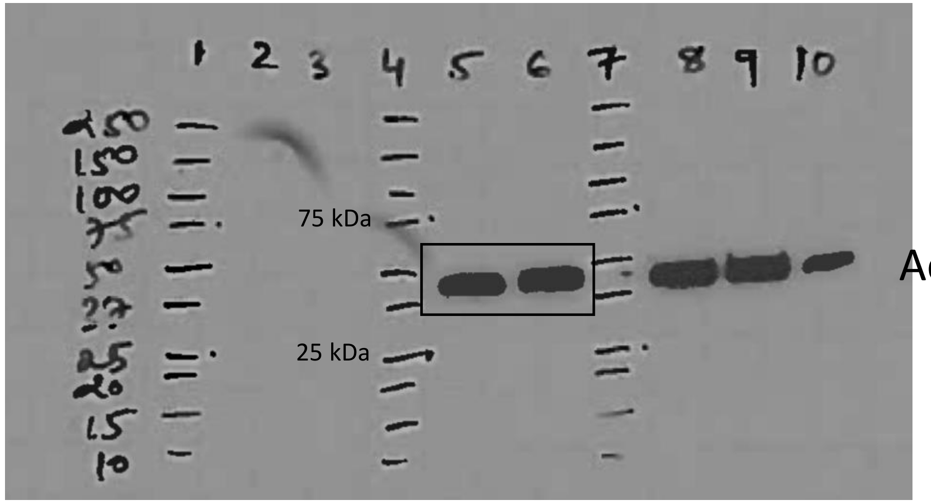

Actin

Supplementary Figure 21: Original scans of immunoblots used in Supplementary Figure 6E showing the expression of GNPNAT1 in 22Rv1 xenograft tumors generated using GNPNAT1 KD and NT controls. Actin was used as control for loading . Boxed area highlights the bands that were included in the main Supplementary Figure 6E.

E

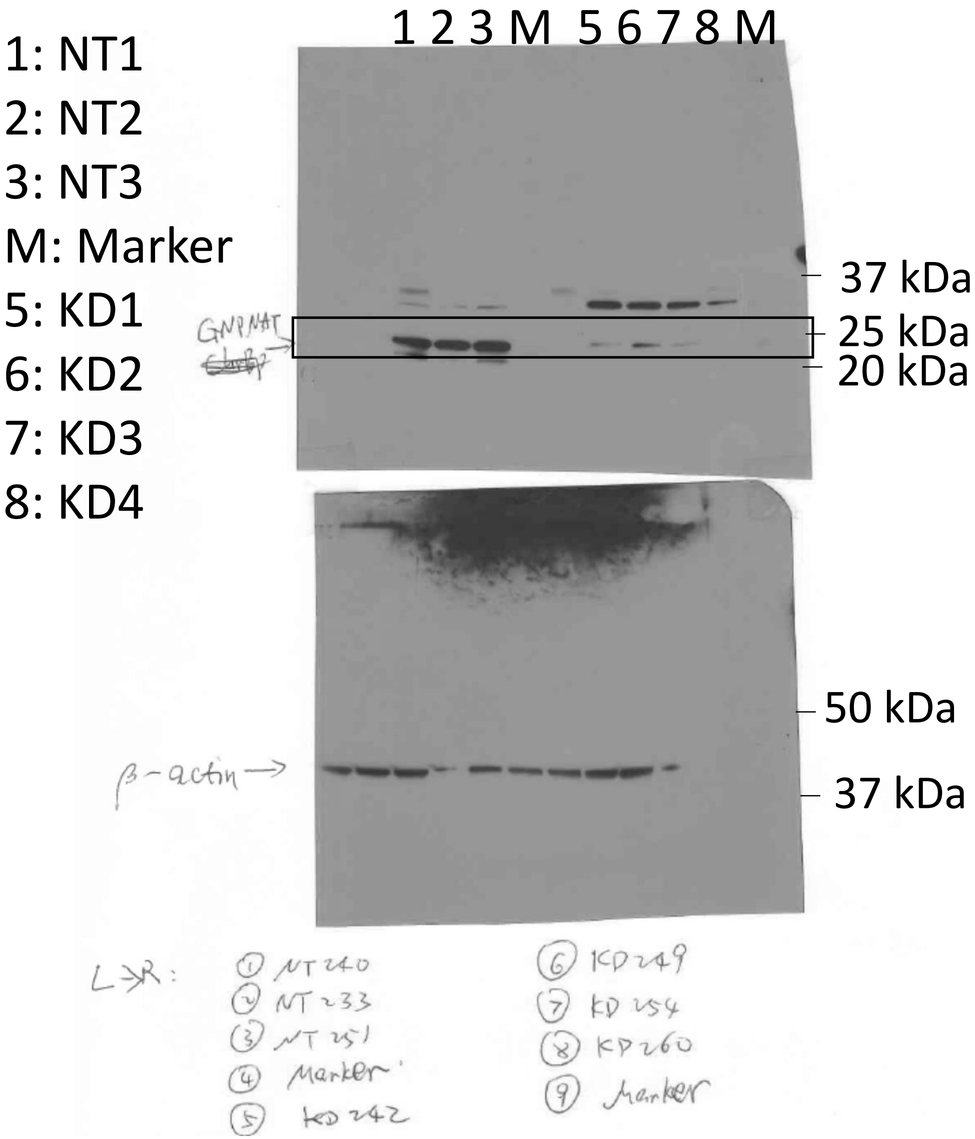

Supplementary Figure 22: Original scans of the immunoblots used in Supplementary Figures 8 B and D showing the downregulation of GFPT1 in both 22Rv1 and LNCaP-ABL cells and moderate changes in AR and AR-V7 in 22Rv1 cells after knockdown of GFTP1 using siRNA compared to scrambled control. GAPDH was used as control for loading. Boxed area highlights the bands that were included in the main Supplementary Figures 8 B and D.

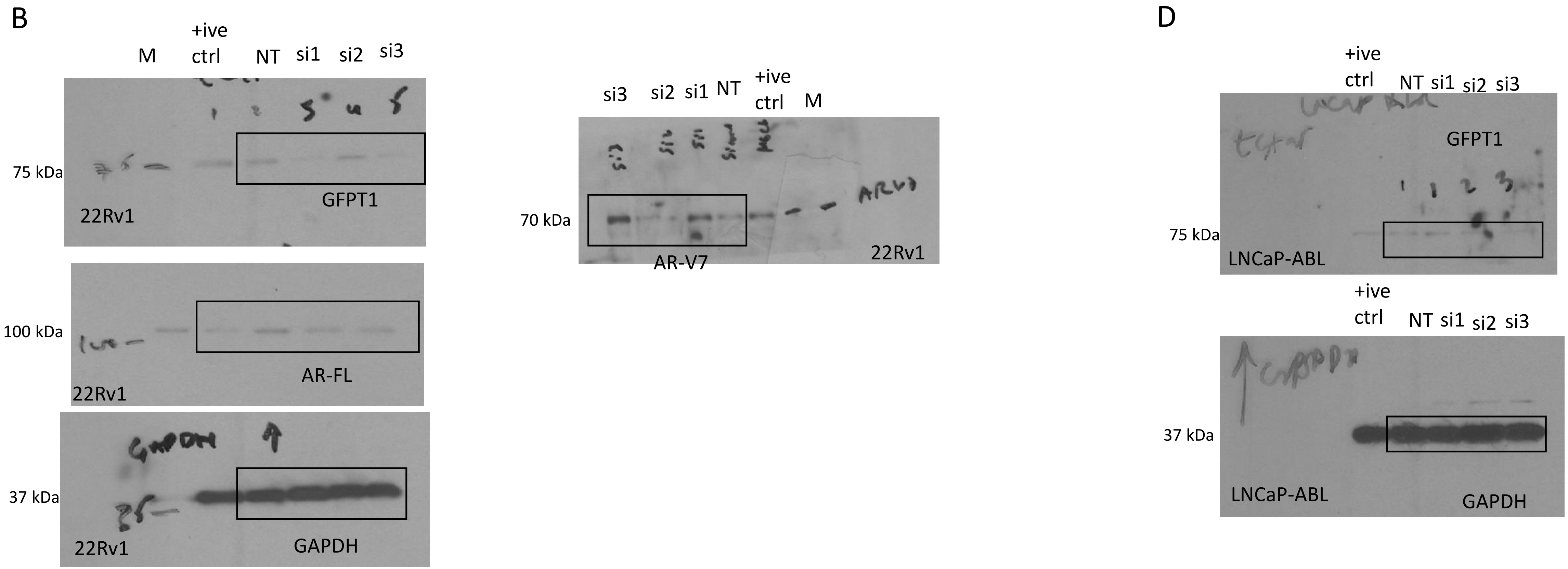

Supplementary Figure 23: Original scans of the immunoblots used in Supplementary Figure 8 I showing the downregulation of GFPT1 in 22Rv1 and LNCaP-ABL cells containing GNPNAT1 knockdown compared to NT control. GAPDH was used as control for loading. Boxed area highlights the bands that were included in the main Supplementary Figure 8 I.

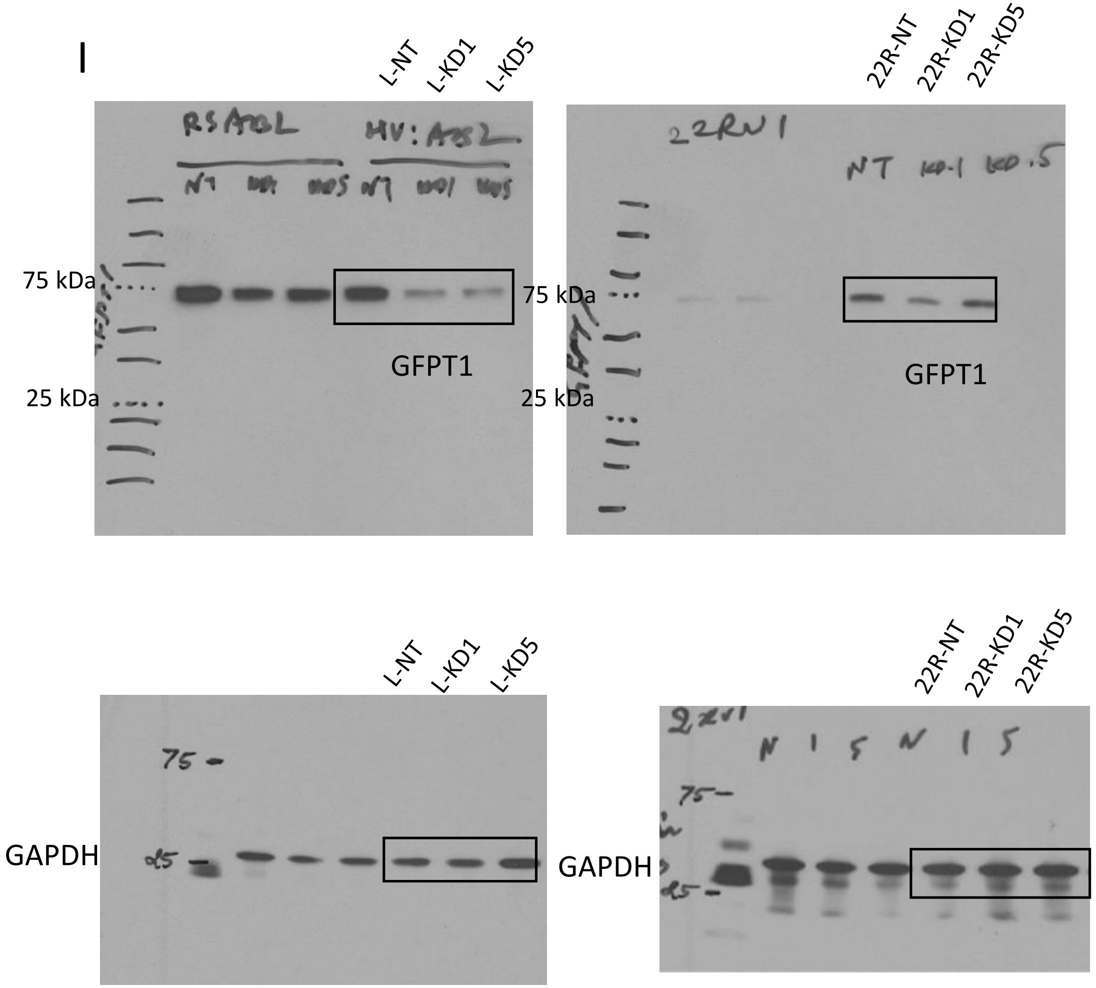

Supplementary Figure 24: Original scans of the immunoblots used in Supplementary Figure 10 A showing the altered levels of proteins associated with PI3K-AKT axis in C4-2 cells containing GNPNAT1 KD compared to NT control. Actin was used as control for loading. Boxed area highlights the bands that were included in the main Supplementary Figure 10 A.

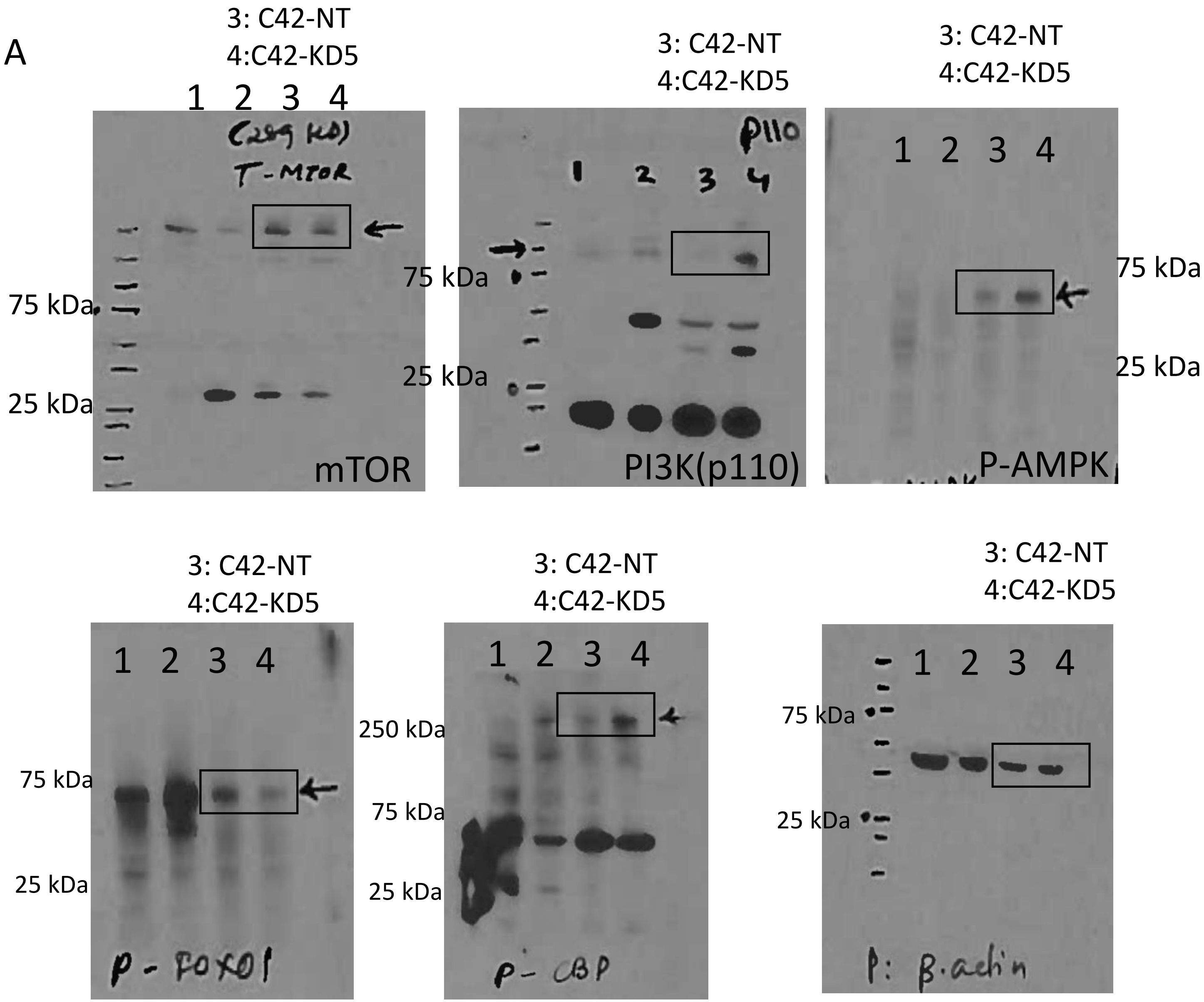

Supplementary Figure 25: Original scans of the immunoblots used in Supplementary Figure 10 B showing the altered levels of proteins associated with PI3K-AKT axis in C4-2 cells containing GNPNAT1 KD compared to NT control. Actin was used as control for loading. Boxed area highlights the bands that were included in the main Supplementary Figure 10 B.

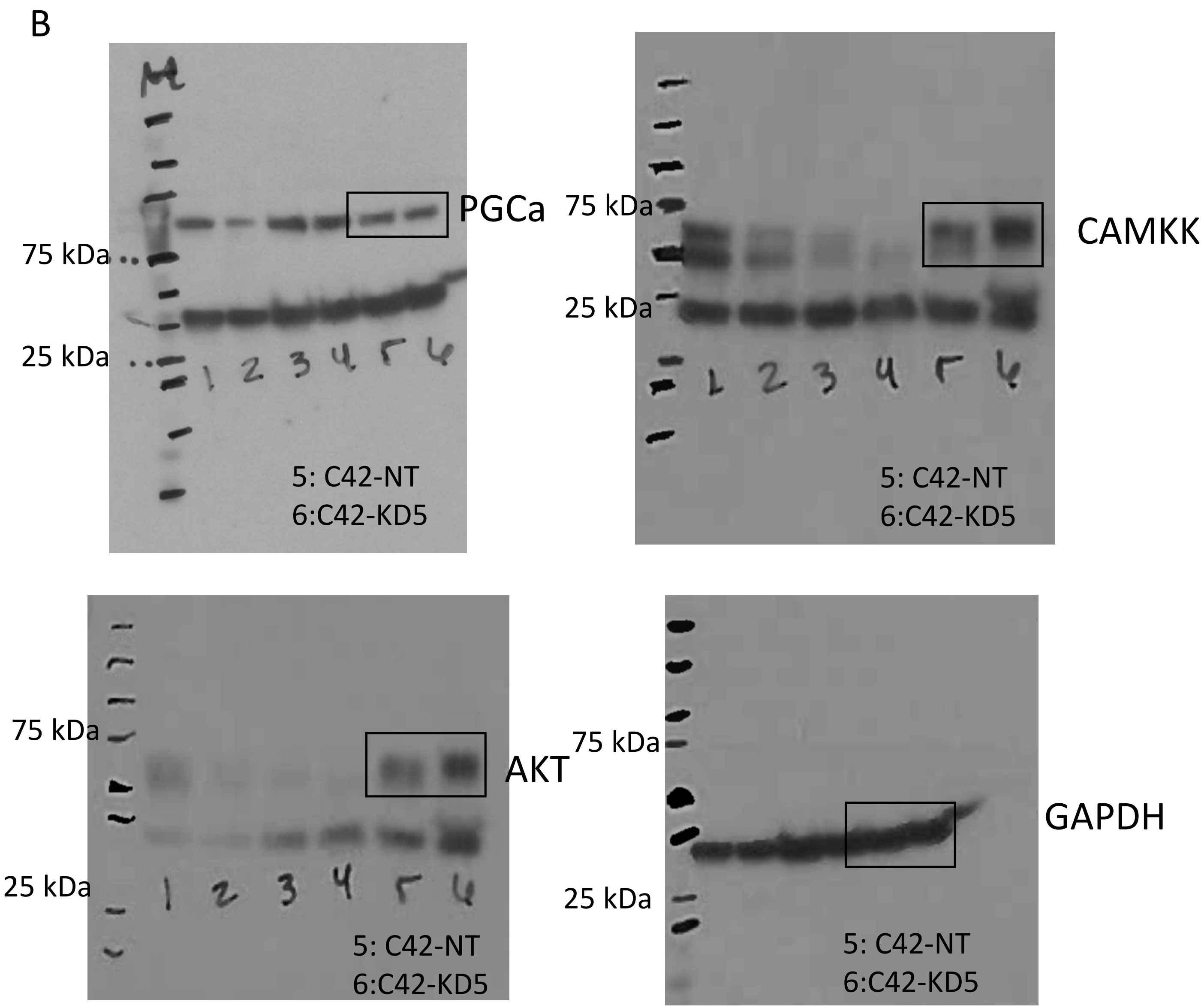

Supplementary Figure 26: Original scans of the immunoblots used in Supplementary Figure 11 C showing unchanged levels of AR-FL and AR-V7 in 22Rv1 cells containing GNPNAT1 KD compared to NT control. GAPDH was used as control for loading. Boxed area highlights the bands that were included in the main Supplementary Figure 11 C.

C

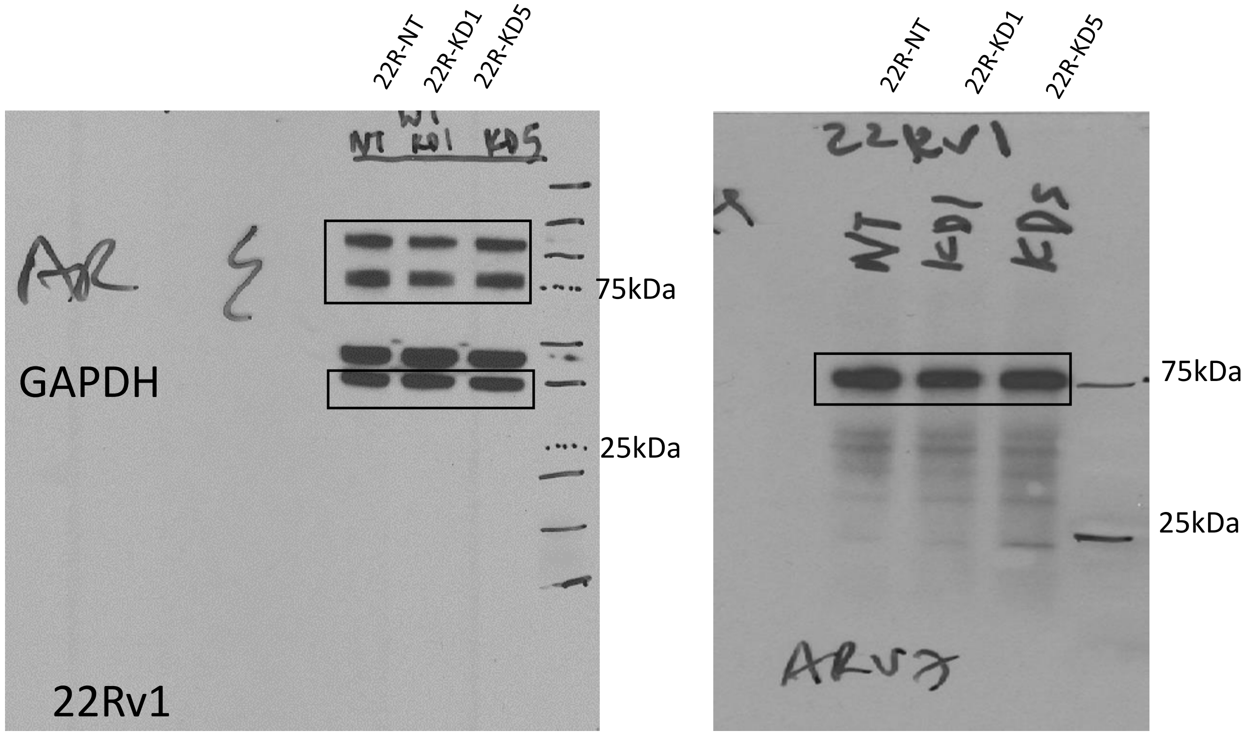

Supplementary Figure 27: Original scans of the immunoblots used in Supplementary Figures 12 A, B showing altered O-linked glycosylation profile with or without UDP-GlcNAc treatment in 22Rv1 cells containing GNPNAT1 knockdown and NT control. GAPDH was used as control for loading. Boxed area highlights the bands that were included in the main Supplementary Figures 12 A, B.

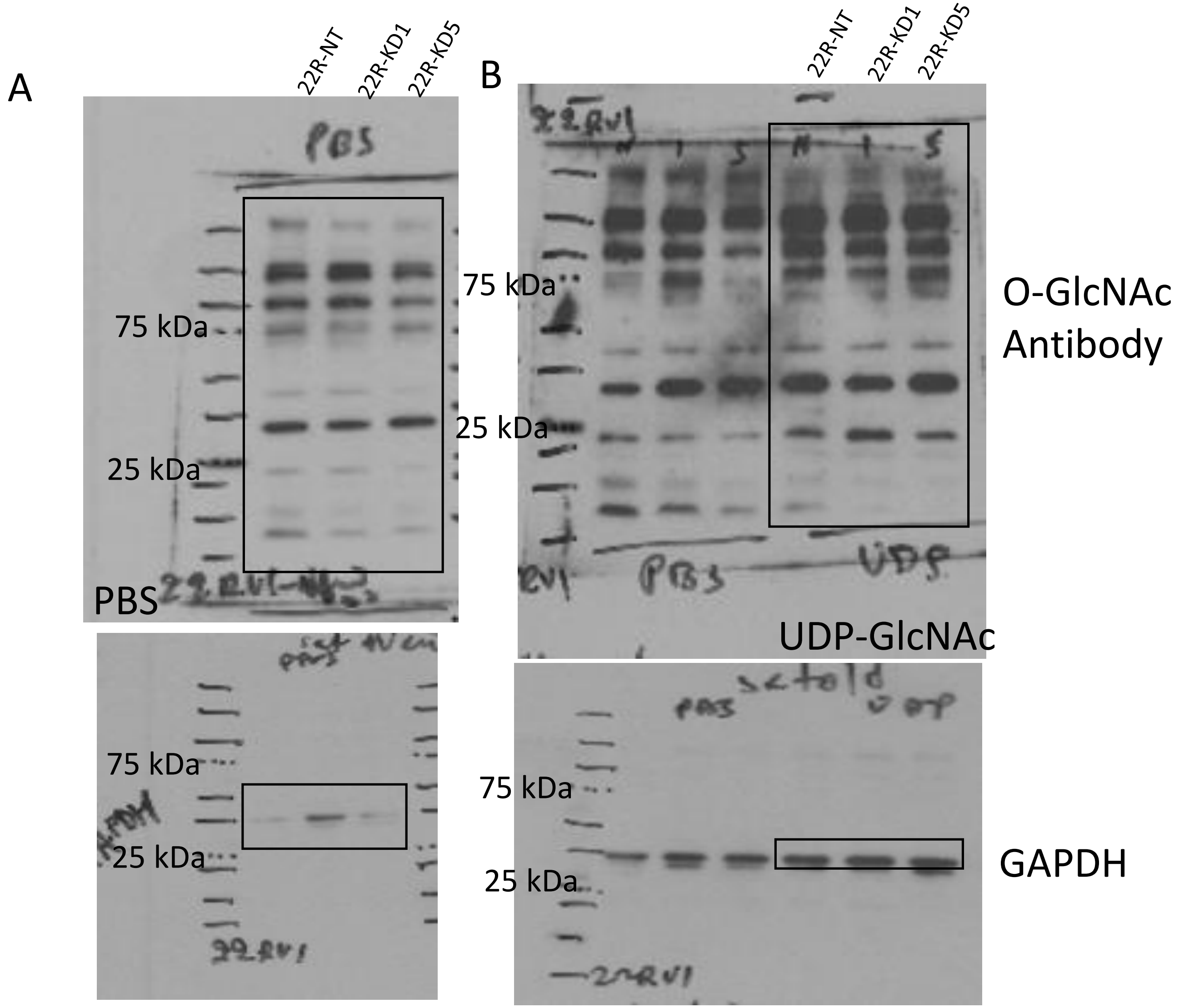

Supplementary Figure 28: Original scans of the immunoblots used in Supplementary Figures 12 C, D showing altered O-linked glycosylation profile with or without UDP-GlcNAc treatment in LNCaP-ABL cells containing GNPNAT1 knockdown compared to NT control. GAPDH was used as control for loading. Boxed area highlights the bands that were included in the main Supplementary Figures 12 C, D.

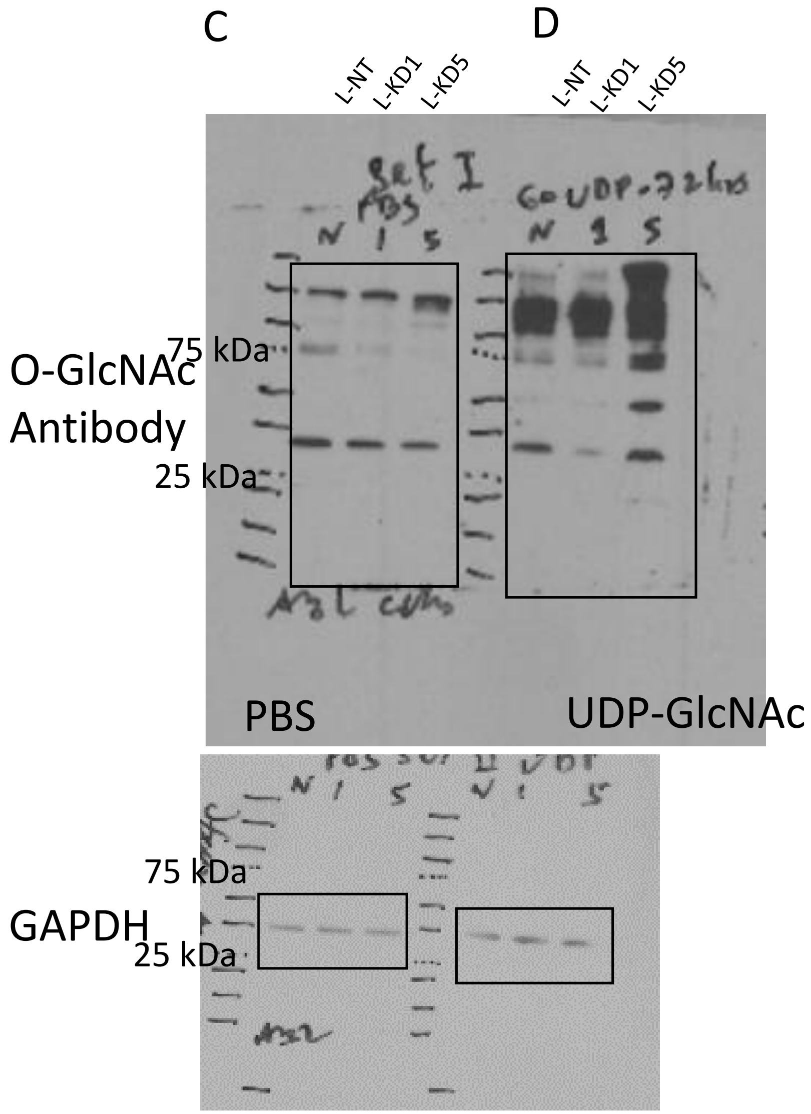

Supplementary Figure 29: Original scans of the immunoblots used in Supplementary Figure 13 B, showing elevated levels of ChREBP in both cytoplasmic and nuclear extracts of 22Rv1 cells containing GNPNAT1 KD compared to control. GAPDH and EZH2 were used as controls to verify the degree of enrichment of the cytoplasmic and nuclear extracts, respectively. Boxed area highlights the bands that were included in the main Supplementary Figure 13 B.

B

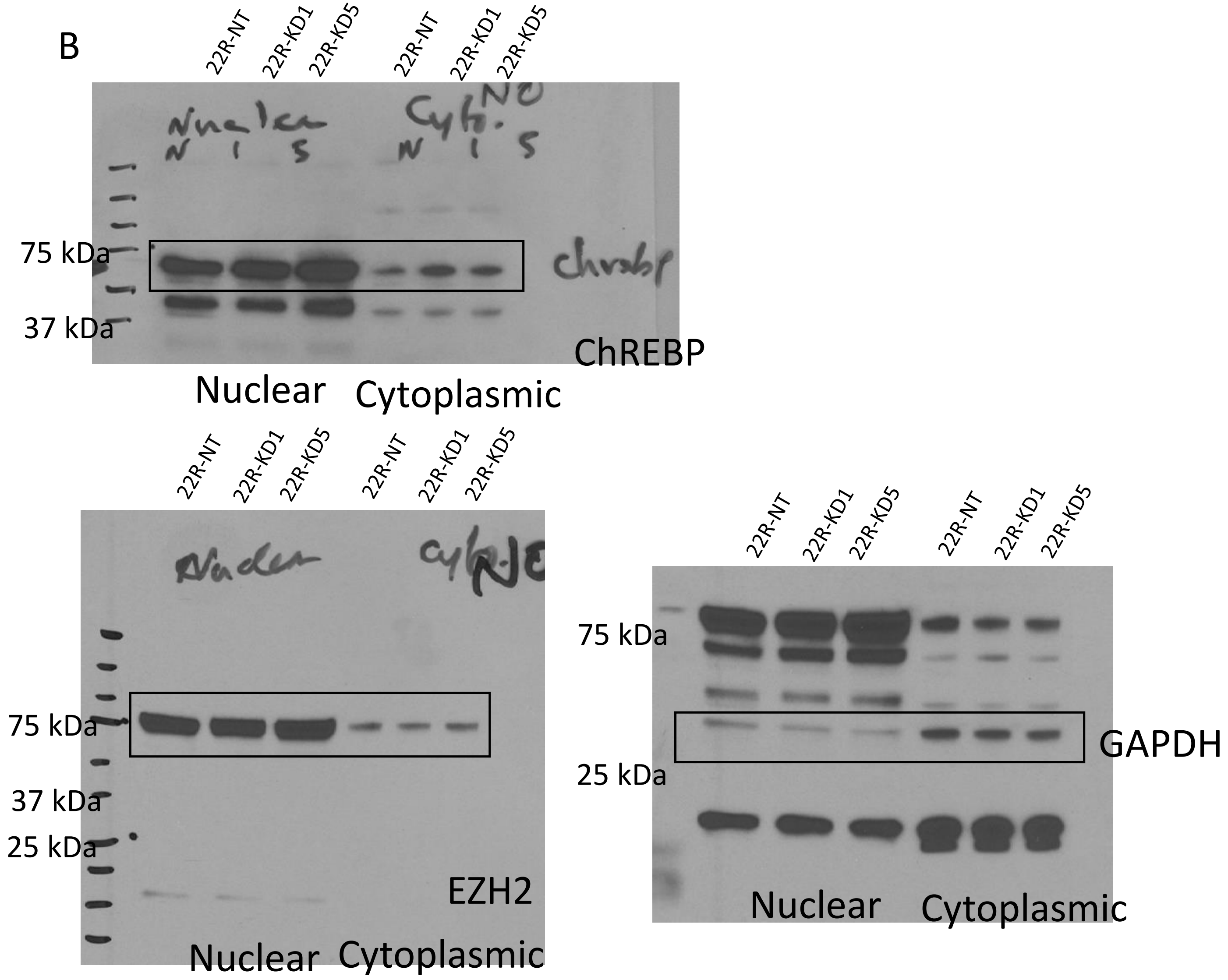

Supplementary Table 1: Clinical information associated with tissue specimens used for metabolomic profiling taken from previously published study[1].

**Characteristic**	**Value+**
**Benign: Benign adjacent prostate tissues from patients with prostate cancer**	
No. of patients	16*
Age at biopsy (years)	56 + 6.7 [40, 63]
Race	
White(non-Hispanic origin)	12 (92.3%)
Other	1 (7.7%)

**PCA: Patients with clinically localized prostate cancer**	
No. of patients	11*
Age at biopsy (years)	57 + 7.7 [40, 63]
Sample Gleason Grade (minor + major)	
3 + 3	3 (25%)
3 + 4	5 (41.7%)
4 + 3	3 (25%)
4 + 4	1 (8.3%)
Baseline PSA	10.4 + 8.1 [2.4, 24.6]
Stage	
T2a	3 (30%)
T2b	4 (40%)
T3a	2 (20%)
T3b	0 (0%)
T4	1 (10%)
Race	

White (non-Hispanic origin) (%)	8 (80%)
Other (%)	2 (20%)

*There are 16 benign tissue samples from 16 men. Clinical information is available for 13 of these men. There are 12 local prostate cancer tumor samples from 11 men. Clinical information is available for 10 of these men. There are 14 metastatic tumor samples from 13 men. Clinical information is available on all 13 men.

Supplementary Table 2: Outliers p-value for integrative score of top 5 pathways**.**

Pathway	Outliers p-value
Riboflavin metabolism	<0.0001
Biotin metabolism	0.0148
Amino sugar metabolism	0.0269
Valine, leucine and isoleucine biosynthesis	0.0285
Cysteine metabolism	0.2117

Supplementary Table 3: Pathway combined scores determined using integration of gene-expression and metabolomics data derived from PCa patient samples.

	Ranking (Combined Score)	Average Gene Ranking	Average Metabolic Ranking	Robustness (from sensitivity analysis)	Number of Significance Neighbors/Total Number of Neighbors
Riboflavin Metabolism	1	**<5**	>5	**100%**	2/3 (*P* = **0.1127**)
Biotin Metabolism	2	**<5**	>5	82%	1/2 (*P* = **0.3944**)
Amino sugar Metabolism (HBP)	3	>5	>5	**100%**	**5/8 (*P* = 0.0060)**
Valine Leucine and Isoleucine Biosynthesis	4	>5	>5	**100%**	2/5 (*P* = **0.2977**)
Cysteine Metabolism	5	>5	**<5**	56%	2/5 (*P* = **0.2971**)

Supplementary Table 4: MRM transition of metabolites used for steady state quantification.

Compound Name	Precursor Ion	Product Ion	Polarity	Separation Type	Gradient Type	Column
UDP-N-Acetylglucosamine	606	385	Negative	Reverse Phase	A	RPMAX
UDP-N-Acetylglucosamine	606	282	Negative	Reverse Phase	A	RPMAX
UDP-N-Acetylglucosamine	606	79	Negative	Reverse Phase	A	RPMAX
N-AcetylGlucosamine-6-Phosphate	300.1	199	Negative	Reverse Phase	A	RPMAX
N-AcetylGlucosamine-6-Phosphate	300.1	97	Negative	Reverse Phase	A	RPMAX
Glucosamine-6-Phosphate	258.1	199.1	Negative	Reverse Phase	A	RPMAX
Glucosamine-6-Phosphate	258.1	97.1	Negative	Reverse Phase	A	RPMAX

Supplementary Table 5: List of primer sequences used in this study.

List of primers	5'-(Sequence)-3'
AR_FL_(Exon6-7)F	CAAATCACCCCCCAGGAAT
AR_FL_(Exon6-7)R	CACTGGAATAATGCTGAAGAGTAGCA
AR-V7-F	TGCCAACCCGGAATTTTTCT
AR-V7-R	GTCGTCTTCGGAAATGTTATGAA
HK2_FP	GAGCCACCACTCACCCTACT
HK2_RP	CCAGGCATTCGGCAATGTG
UAP1_FP	CTCCAGGCCATGAACTTTGAG
UAP1_RP	TCCATTCGTGCATCCACATTC
PSA_FP	CCTGTCCGTGACGTGGAT
PSA_RP	CAGGGTTGGGAATGCTTCT
NEK2_F	TGCTTCGTGAACTGAAACATCC
NEK2_R	CCAGAGTCAACTGAGTCATCACT
CENPE_F	GATTCTGCCATACAAGGCTACAA
CENPE_R	TGCCCTGGGTATAACTCCCAA
BUB1_F	TGGGAAAGATACATACAGTGGGT
BUB1_R	AGGGGATGACAGGGTTCCAAT
MAD2L1_F	GTTCTTCTCATTCGGCATCAACA
MAD2L1_R	GAGTCCGTATTTCTGCACTCG
CDC25A_F	GTGAAGGCGCTATTTGGCG
CDC25A_R	GTGAAGGCGCTATTTGGCG
VMYC_F	GGCTCCTGGCAAAAGGTCA

VMYC_R	CTGCGTAGTTGTGCTGATGT
CEP70_F	AGAACAACGAGCTAATGACTTGG
CEP70_R	AGCCCTACTTAGTGATTCATCCT
SMC4_F	CGCCTCCAGCAATGACCAAT
SMC4_R	CCCCAGCATAGGATTTGAAGTT
E2F3_F	AGAAAGCGGTCATCAGTACCT
E3F3_R	TGGACTTCGTAGTGCAGCTCT
GNPNAT1_FP	CCTCTACGGACCTTACTAGAAAAA
GNPNAT1_RP	GACAATTTTCTTACAAGATTTTTACTTTAGAA
GFPT1_FP	AACTACCATGTTCCTCGAACGA
GFPT1_RP	CTCCATCAAATCCCACACCAG
ChREBP_FP	CAAGTGGCGCATCTACTACAA
ChREBP_RP	CGGACTGAGTCATGGTGAAGA
Human_GAPDH_F	GGAGCGAGATCCCTCCAAAAT
Human_GAPDH_R	GGCTGTTGTCATACTTCTCATGG
SP1 ChIP Primers	
ChREBP_F	TTGCACAGAGAAAAGATCAAGG
ChREBP_R	AATTATCAACCGAAAACCCGTA
DHFR_F	GTTAGCACTGCCCTGAGCAT
DHFR_R	GCCAAGCCTAGGAACTGTTG

Supplementary Table 6: List of shRNA sequences used in this study.

GNPNAT1 (Sigma)	Sequence
TRCN0000034621	CCGGGAGTCAGAATACAGCTACATTCTCGAGAATGTAGCTGTATTCTGACTCTTTTTG
TRCN0000034623	CCGGGTTACAAGATTACCCTTGAATCTCGAGATTCAAGGGTAATCTTGTAACTTTTTG
GFPT1 (Sigma)	Sequence
TRCN0000333025	CCGGGCAGATACTTTGATGGGTCTTCTCGAGAAGACCCATCAAAGTATCTGCTTTTTG
TRCN0000363728	CCGGCGTCTTTCTATCCATCGAATTCTCGAGAATTCGATGGATAGAAAGACGTTTTTG

References

1. Sreekumar, A. *et al.* Metabolomic profiles delineate potential role for sarcosine in prostate cancer progression. *Nature* **457**, 910-914 (2009).
