# Supplementary figures and images for: Inhibition of the hexosamine biosynthetic pathway promotes castration-resistant prostate cancer
Source: Nat Commun. 2016 May 19;7:11612. doi: 10.1038/ncomms11612 (PMC4874037; doi:10.1038/ncomms11612)

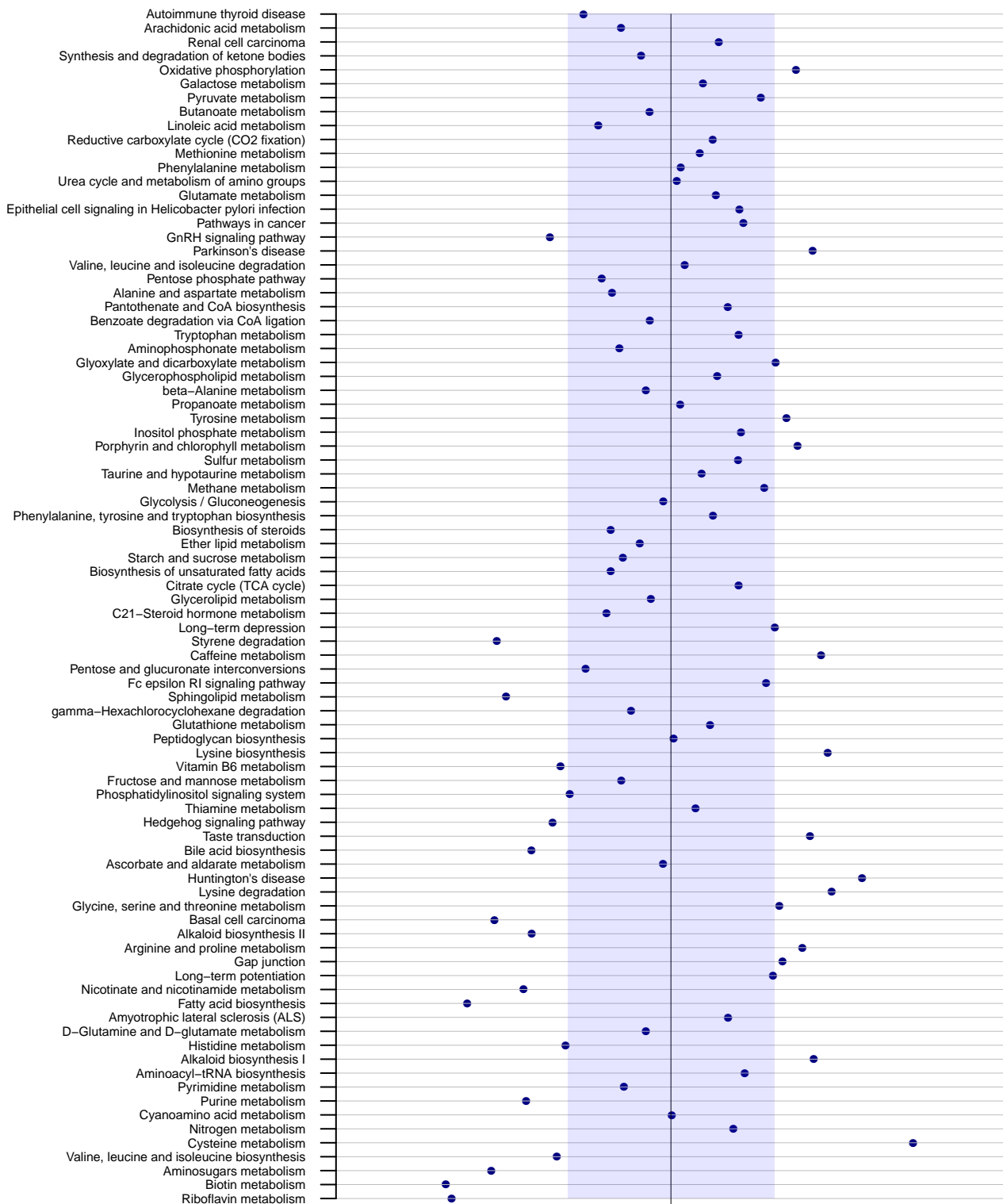

-50 0 50  
Rank Difference (gene - metabolite)

Supplement: Supplementary Software — R software code used for integrative analysis [file ncomms11612-s3.zip › integHBP/Analysis/concordance_ranks.pdf]

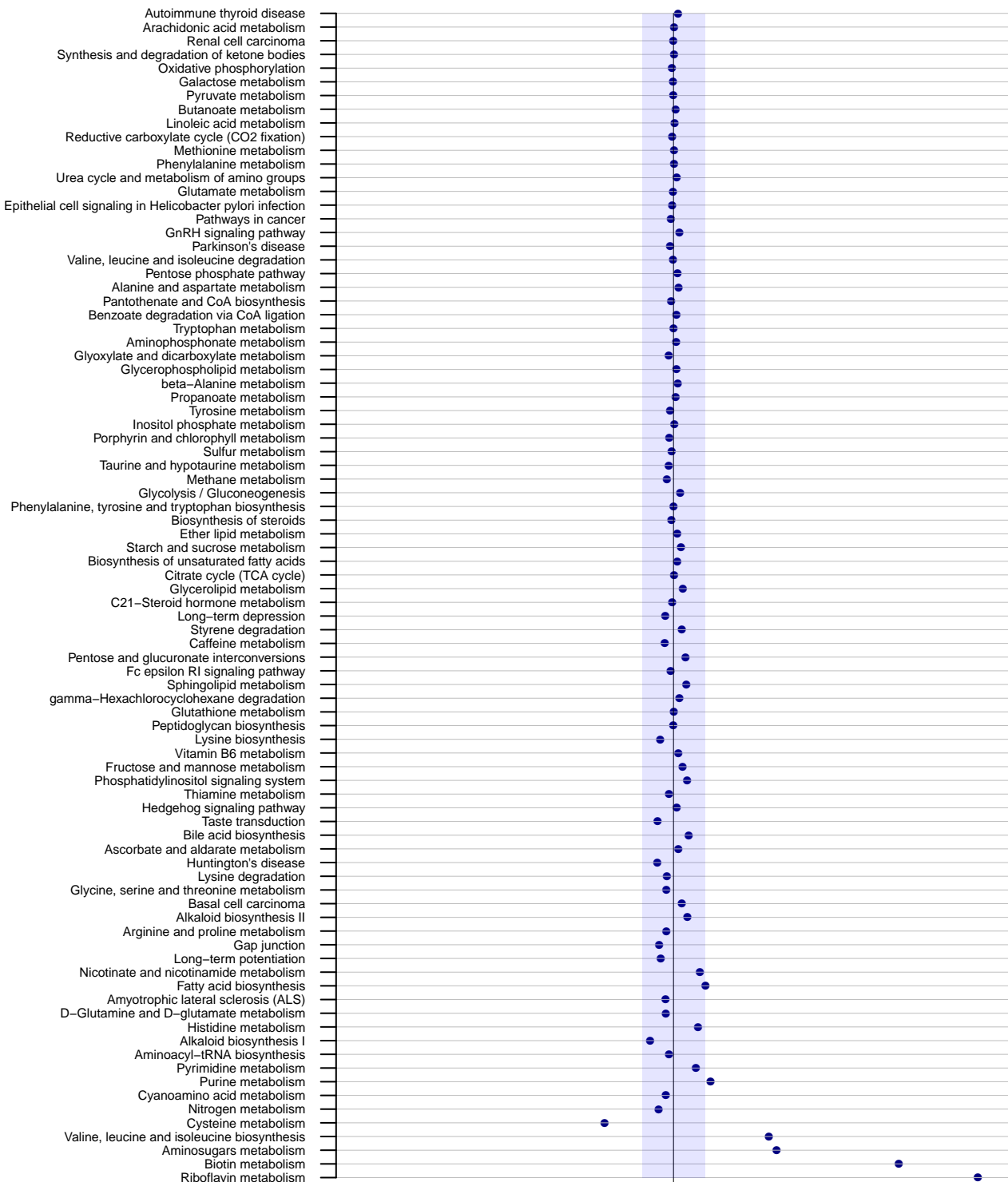

-60 -40 -20 0 20 40 60

Rank Difference (gene – metabolite)

Supplement: Supplementary Software — R software code used for integrative analysis [file ncomms11612-s3.zip › integHBP/Analysis/concordance_scores.pdf]

## Pathway Network Degree Distribution

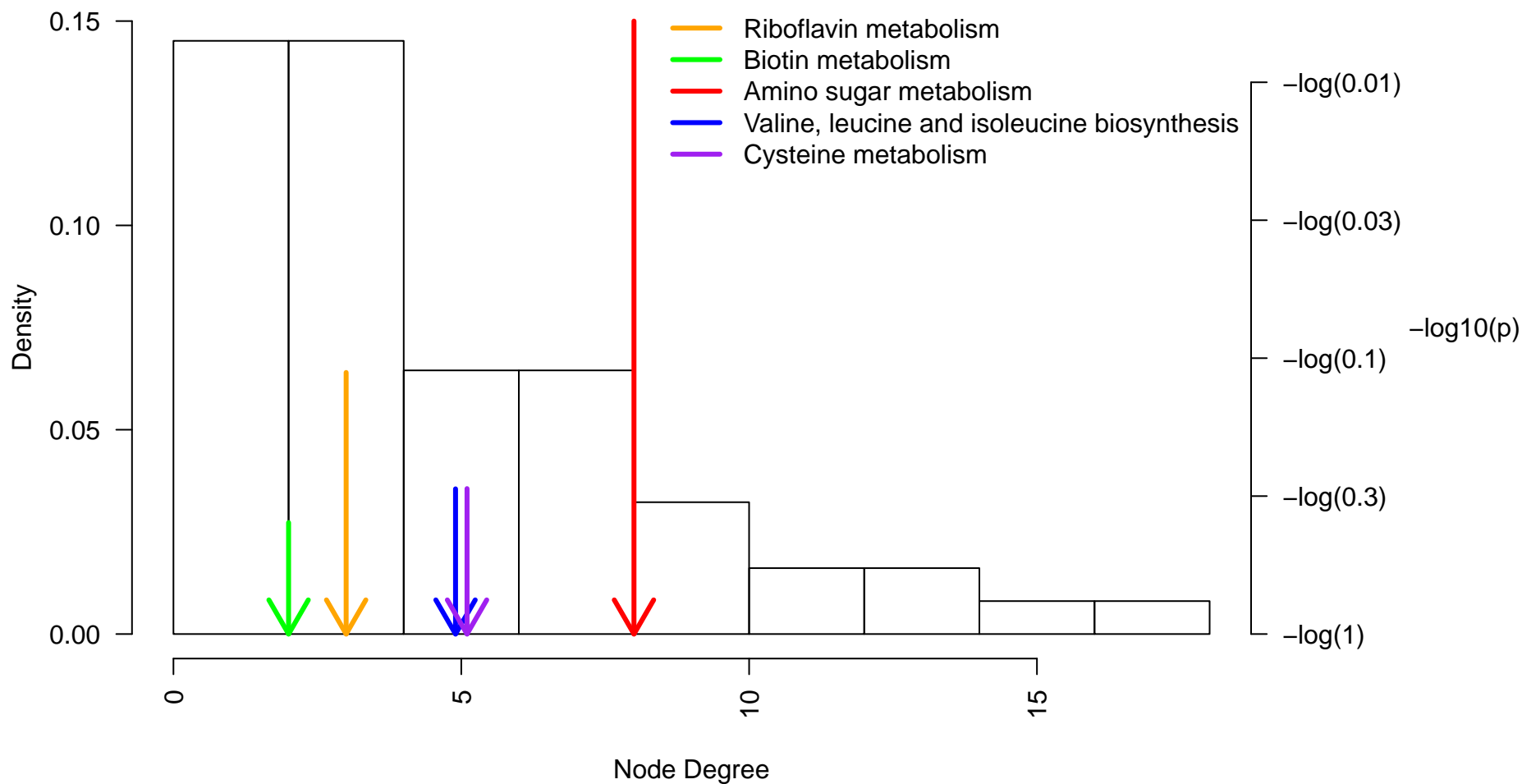

Supplement: Supplementary Software — R software code used for integrative analysis [file ncomms11612-s3.zip › integHBP/Analysis/degdistwsig_010516_seed2.pdf]

## Pathway Network Degree Distribution

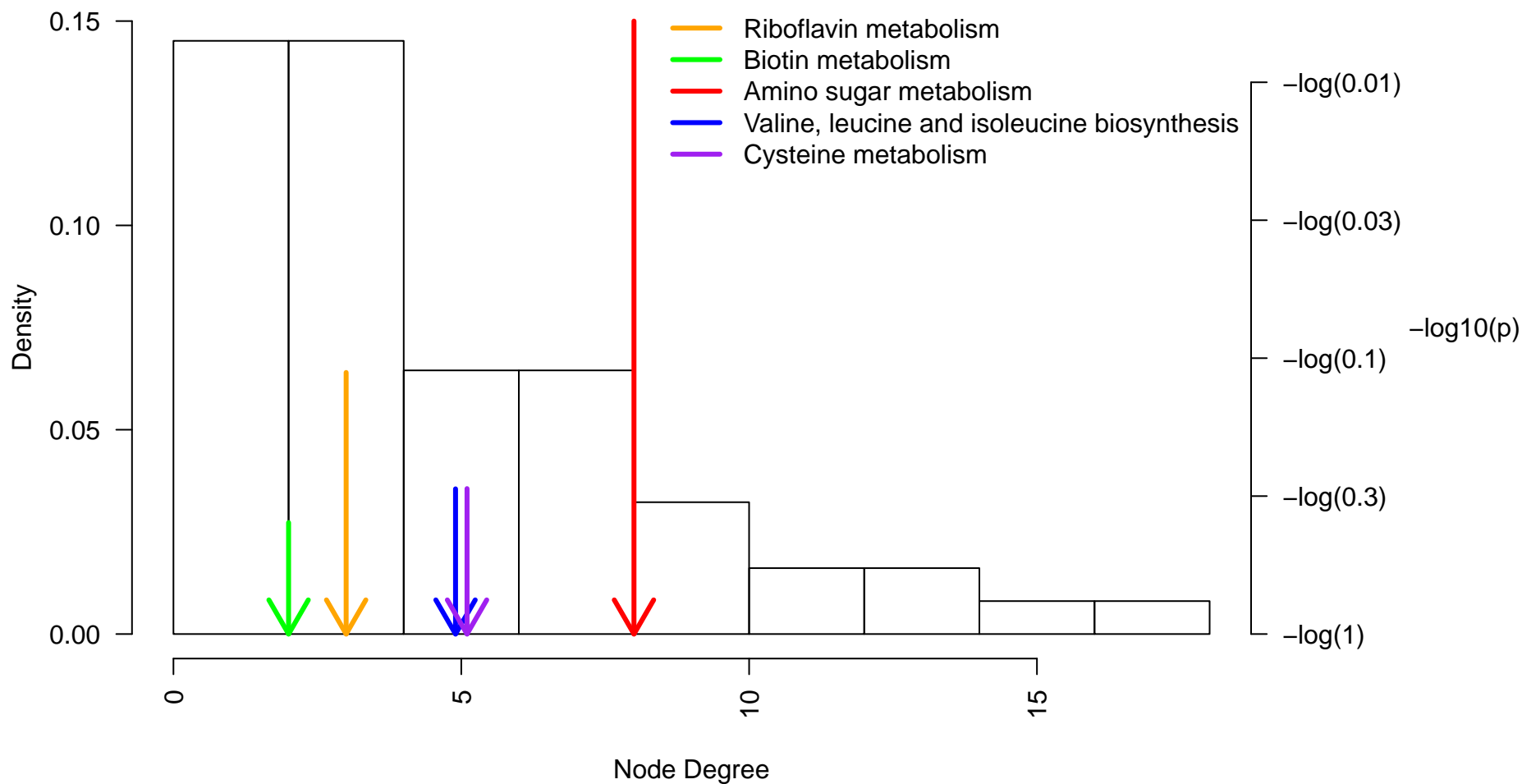

Supplement: Supplementary Software — R software code used for integrative analysis [file ncomms11612-s3.zip › integHBP/Analysis/degdistwsig_010516_seed37.pdf]

## Pathway Network Degree Distribution

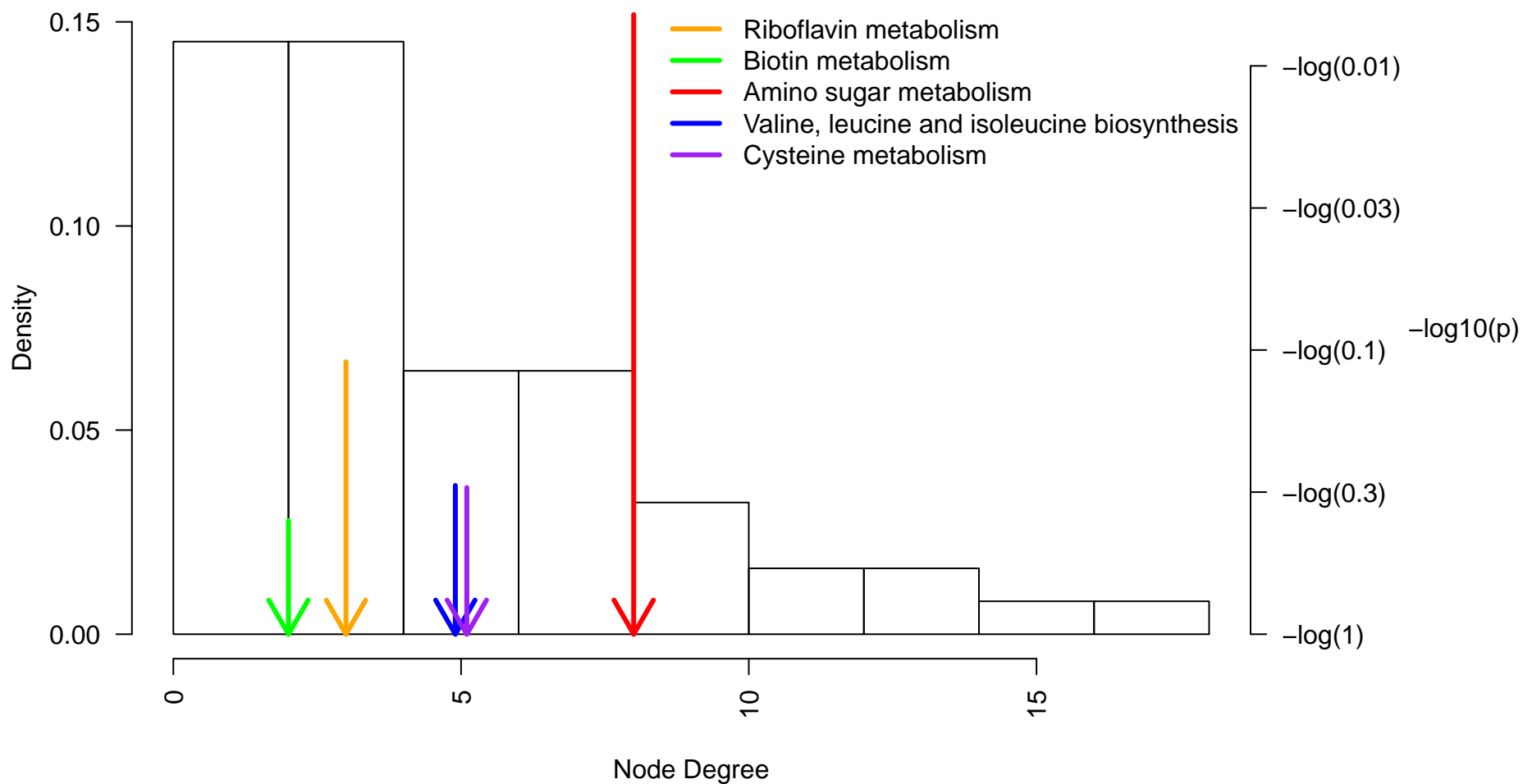

Supplement: Supplementary Software — R software code used for integrative analysis [file ncomms11612-s3.zip › integHBP/Analysis/degdistwsig_042115.pdf]

## Pathway Network Degree Distribution

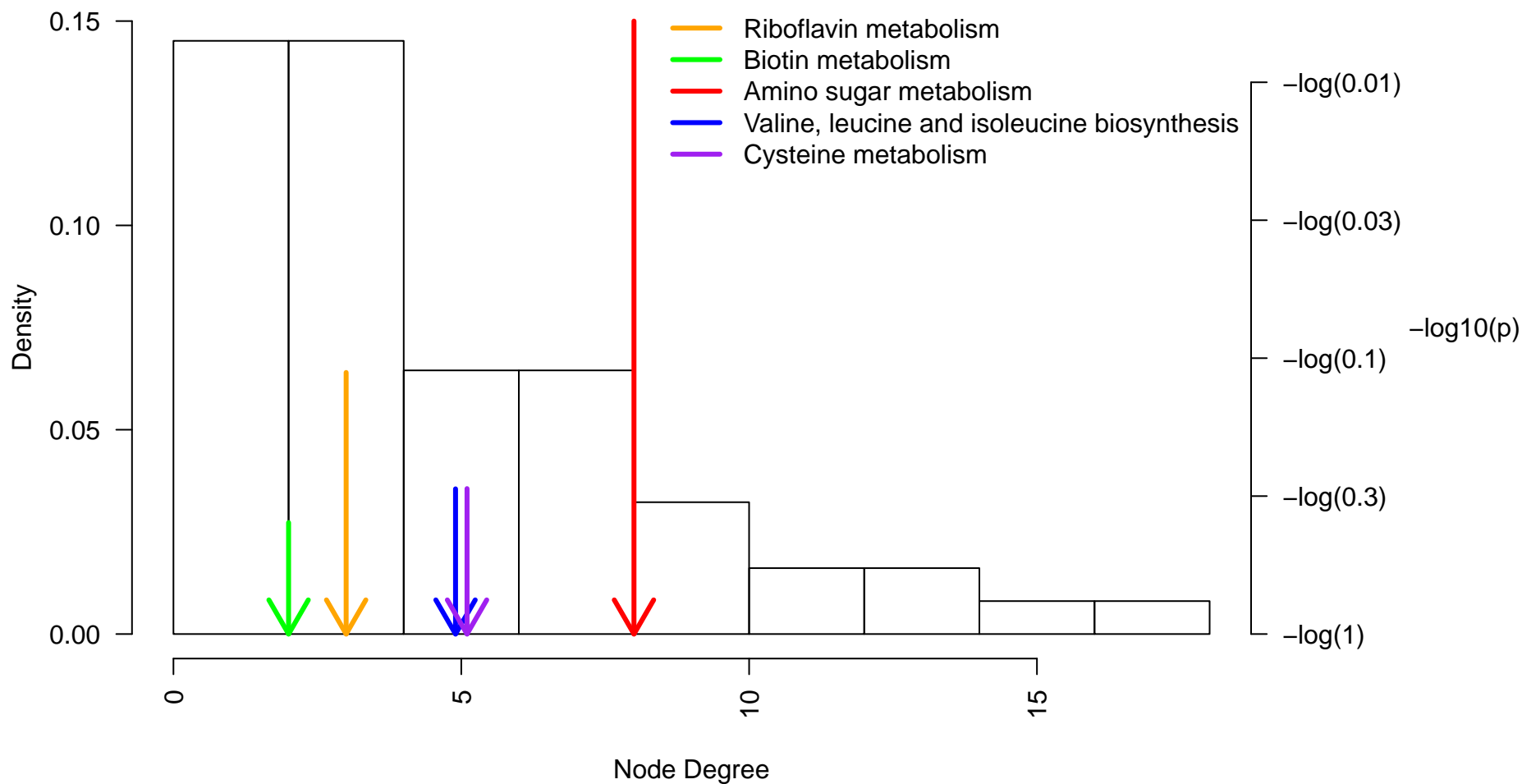

Supplement: Supplementary Software — R software code used for integrative analysis [file ncomms11612-s3.zip › integHBP/Analysis/degdistwsig_120515.pdf]

# Integrative Score

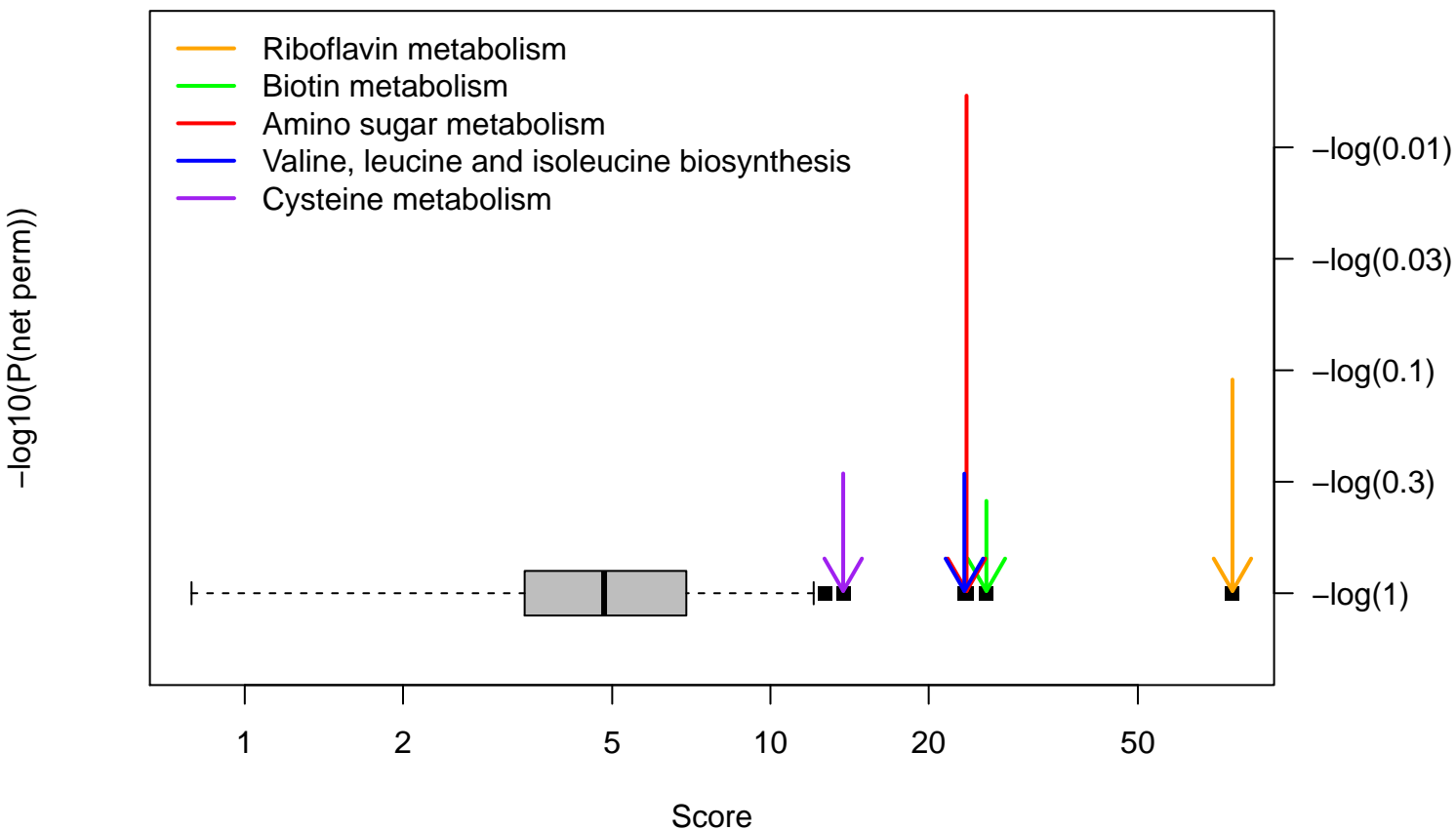

Supplement: Supplementary Software — R software code used for integrative analysis [file ncomms11612-s3.zip › integHBP/Analysis/integscore_permpval_010516_seed2.pdf]

# Integrative Score

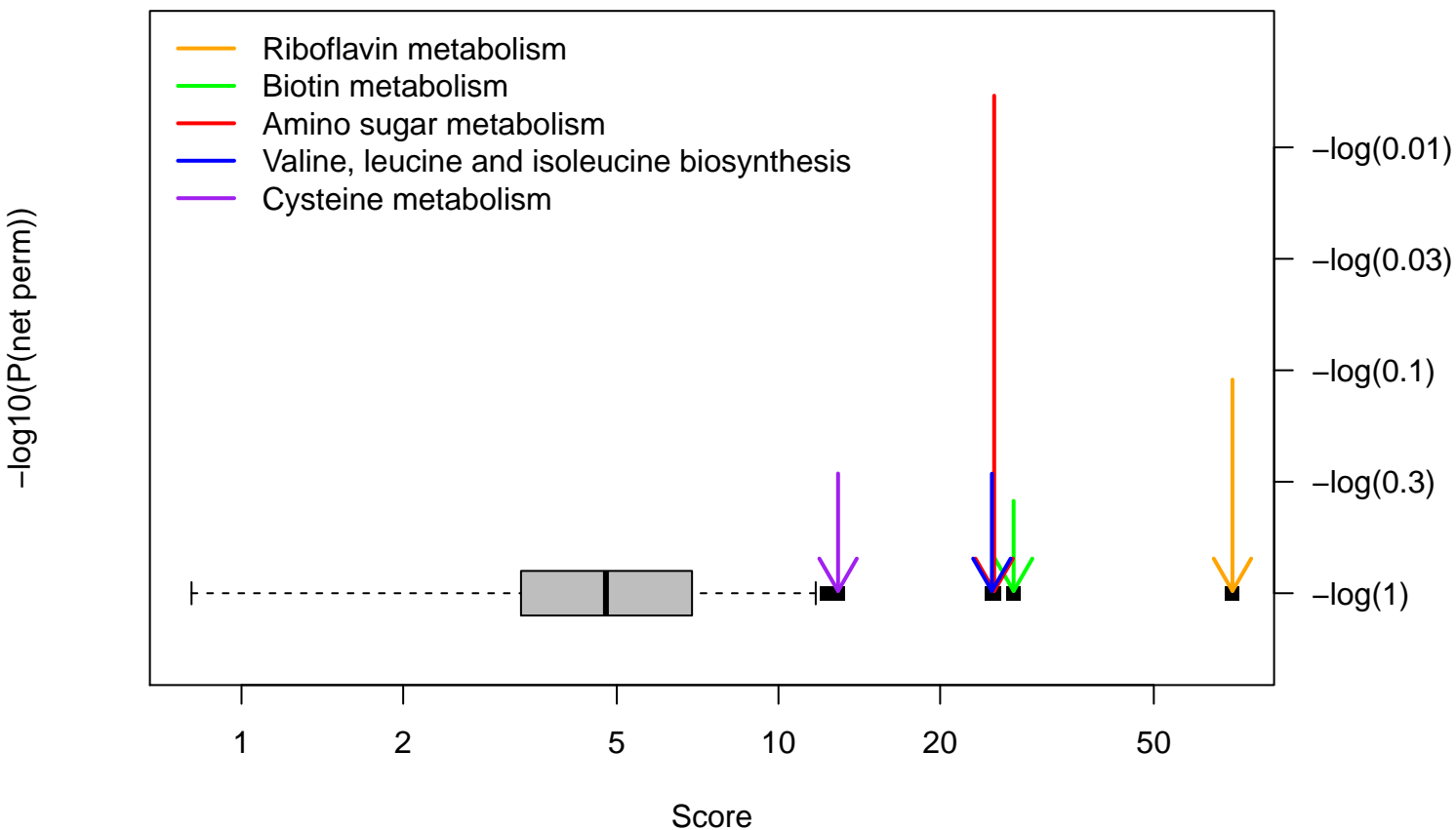

Supplement: Supplementary Software — R software code used for integrative analysis [file ncomms11612-s3.zip › integHBP/Analysis/integscore_permpval_010516_seed37.pdf]

# Integrative Score

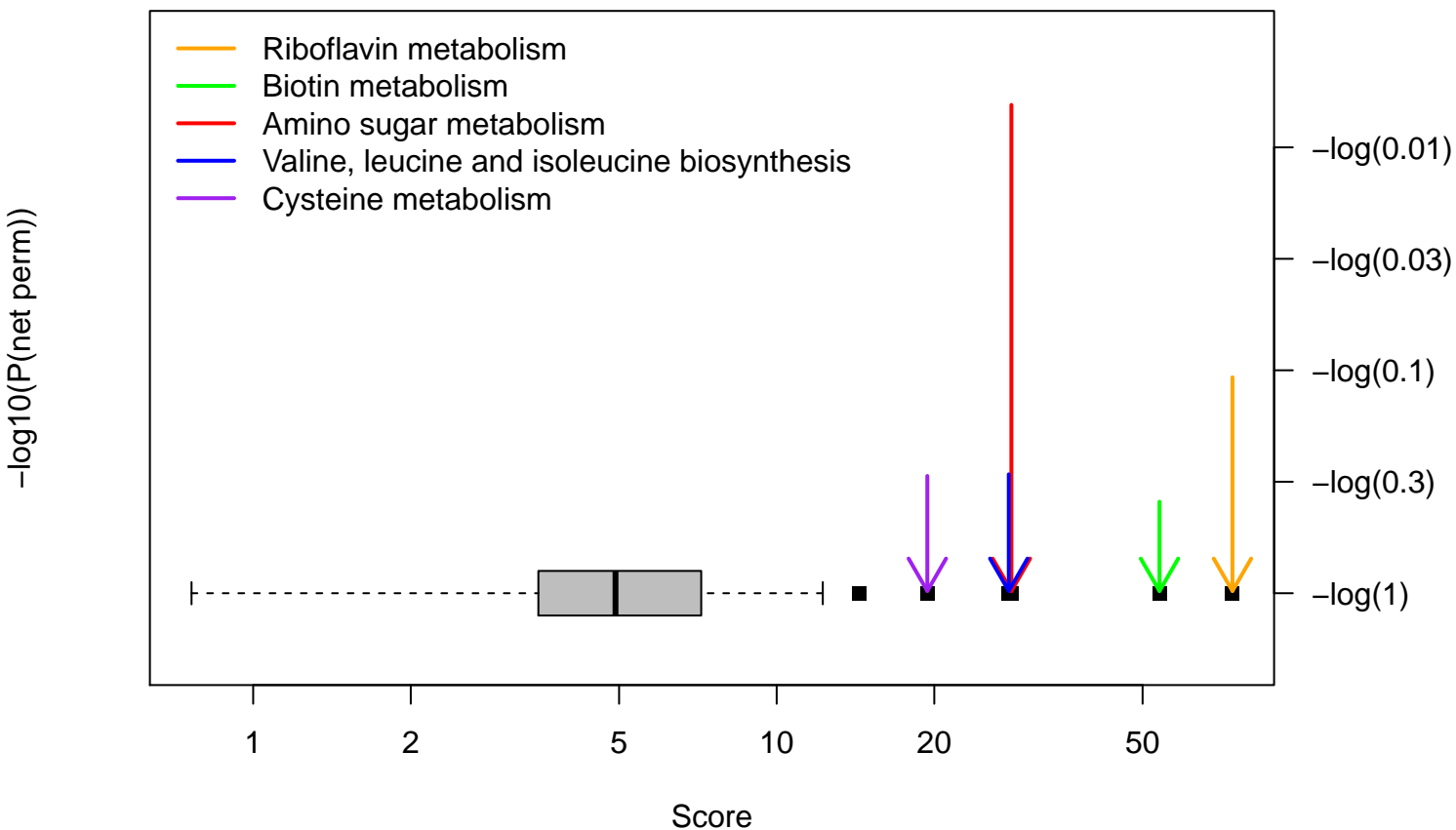

Supplement: Supplementary Software — R software code used for integrative analysis [file ncomms11612-s3.zip › integHBP/Analysis/integscore_permpval_042115.pdf]

# Integrative Score

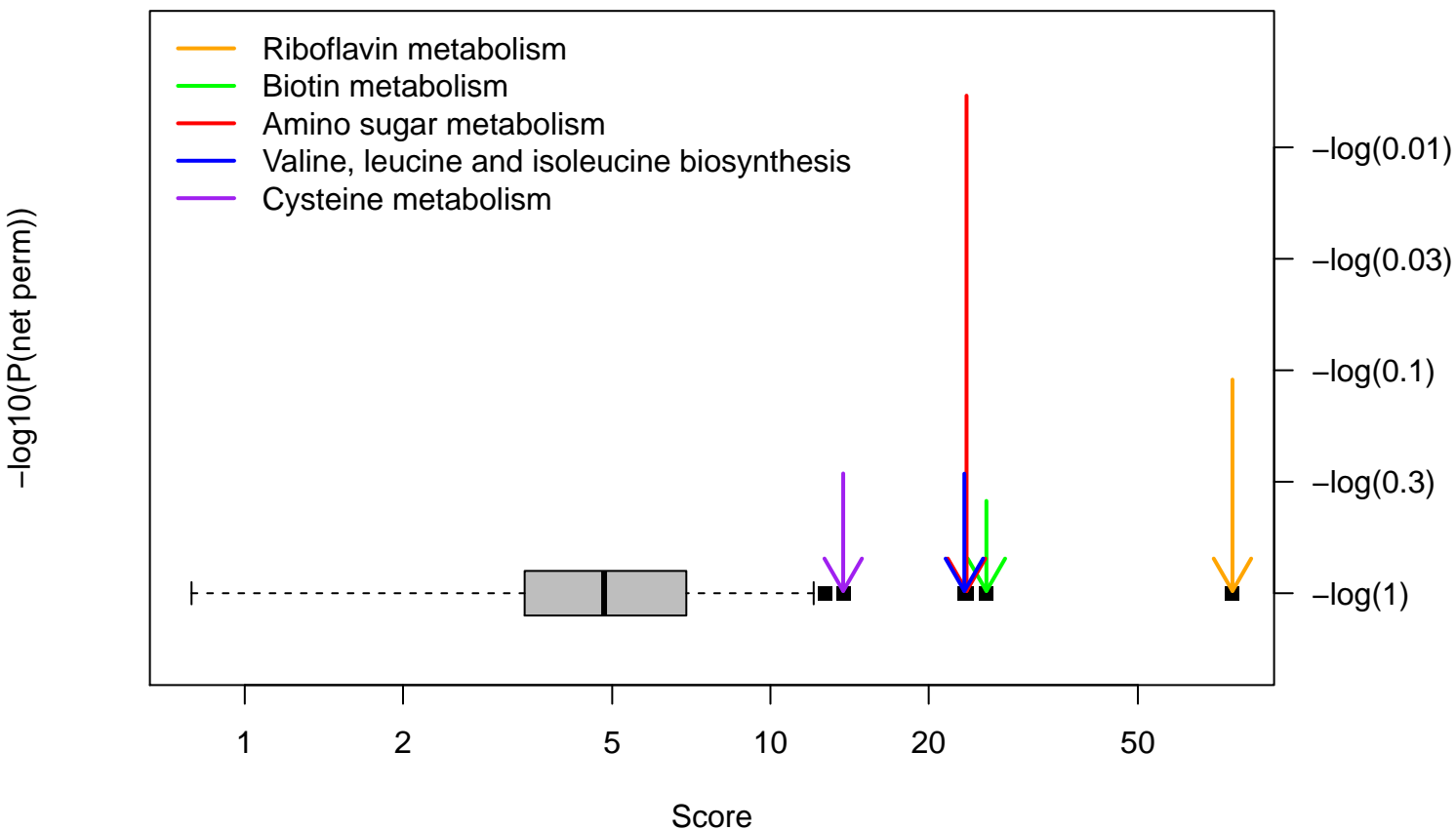

Supplement: Supplementary Software — R software code used for integrative analysis [file ncomms11612-s3.zip › integHBP/Analysis/integscore_permpval_120515.pdf]

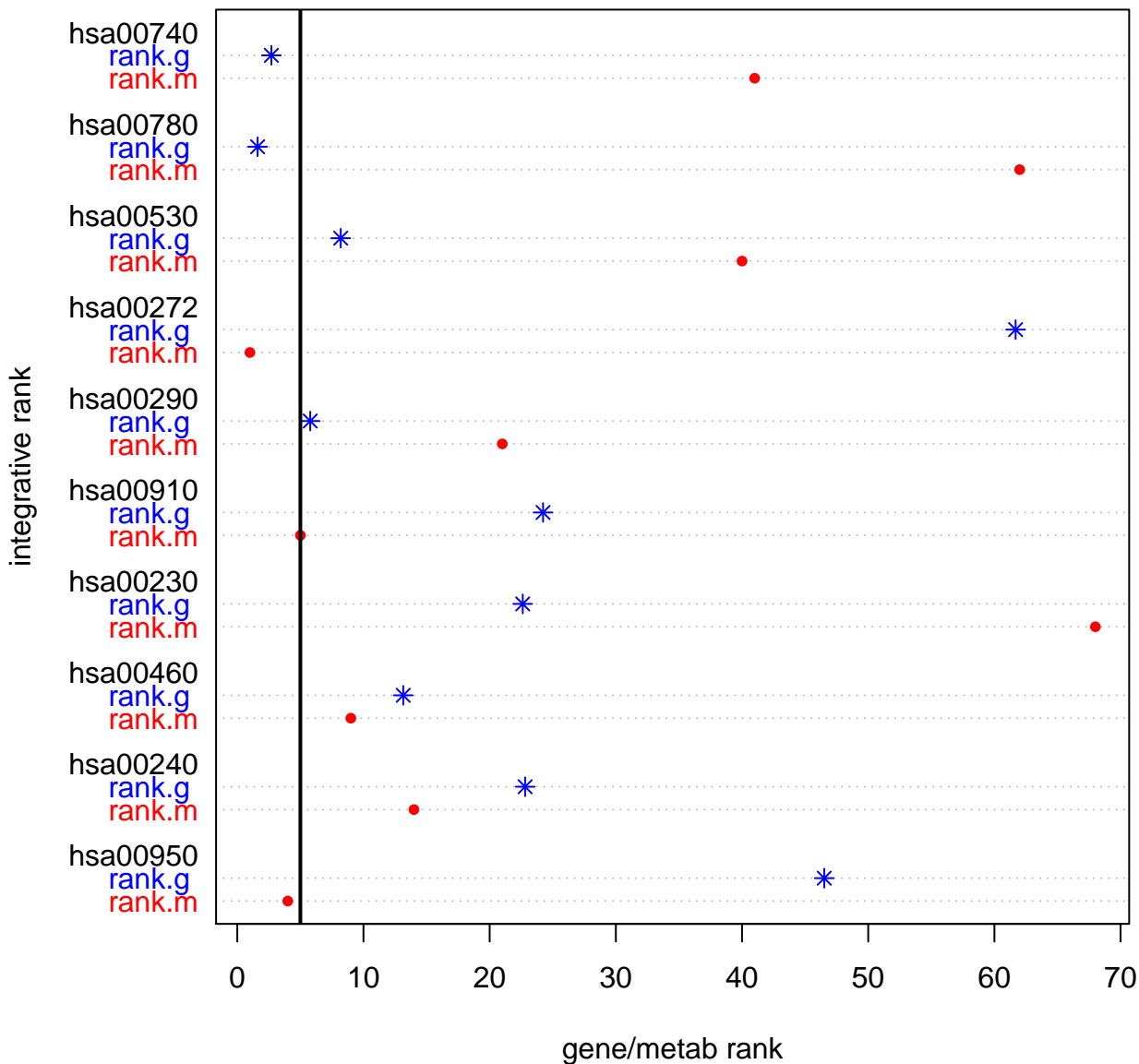

Supplement: Supplementary Software — R software code used for integrative analysis [file ncomms11612-s3.zip › integHBP/Analysis/rankcomp_top10_010516_seed2.pdf]

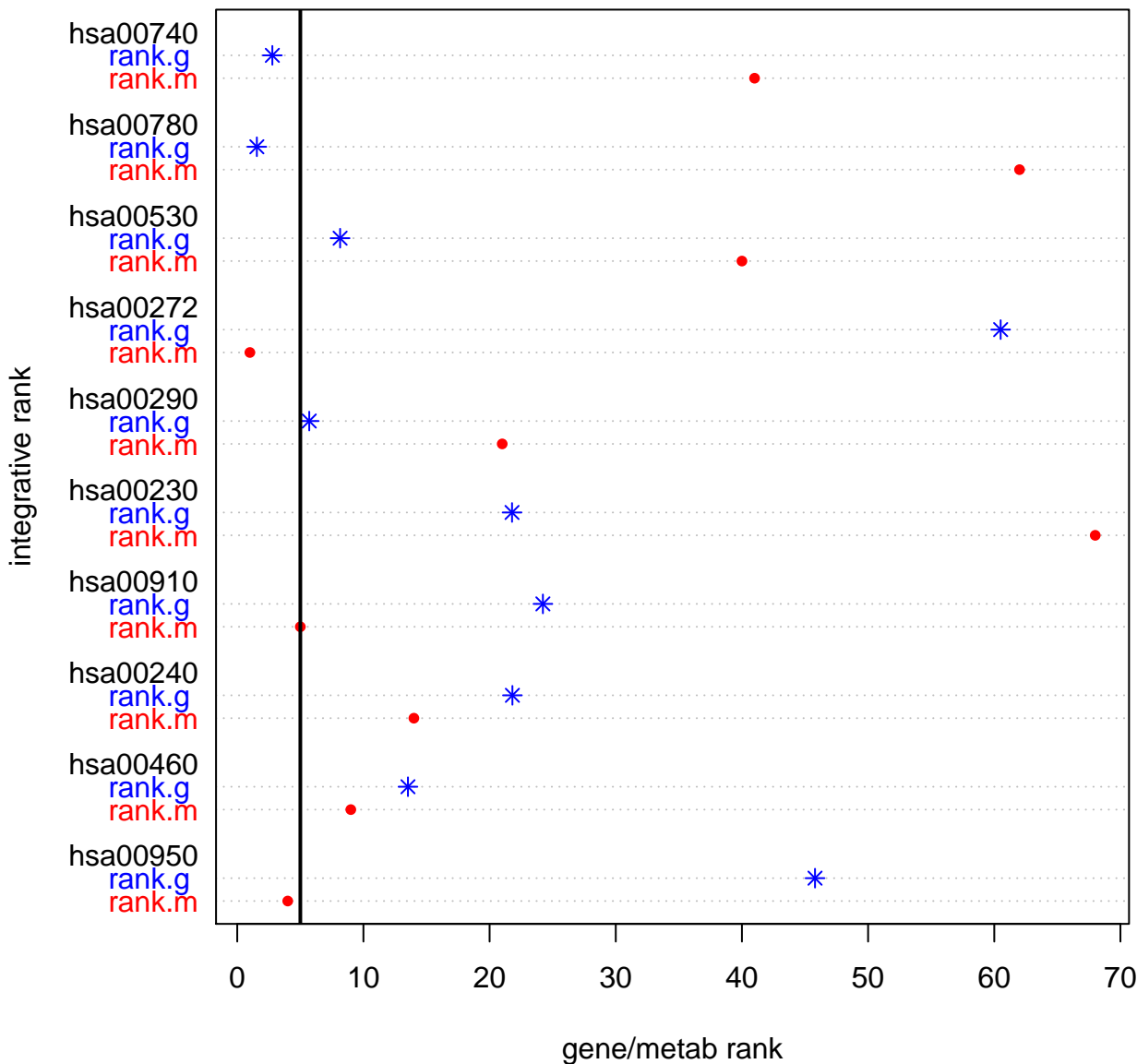

Supplement: Supplementary Software — R software code used for integrative analysis [file ncomms11612-s3.zip › integHBP/Analysis/rankcomp_top10_010516_seed37.pdf]

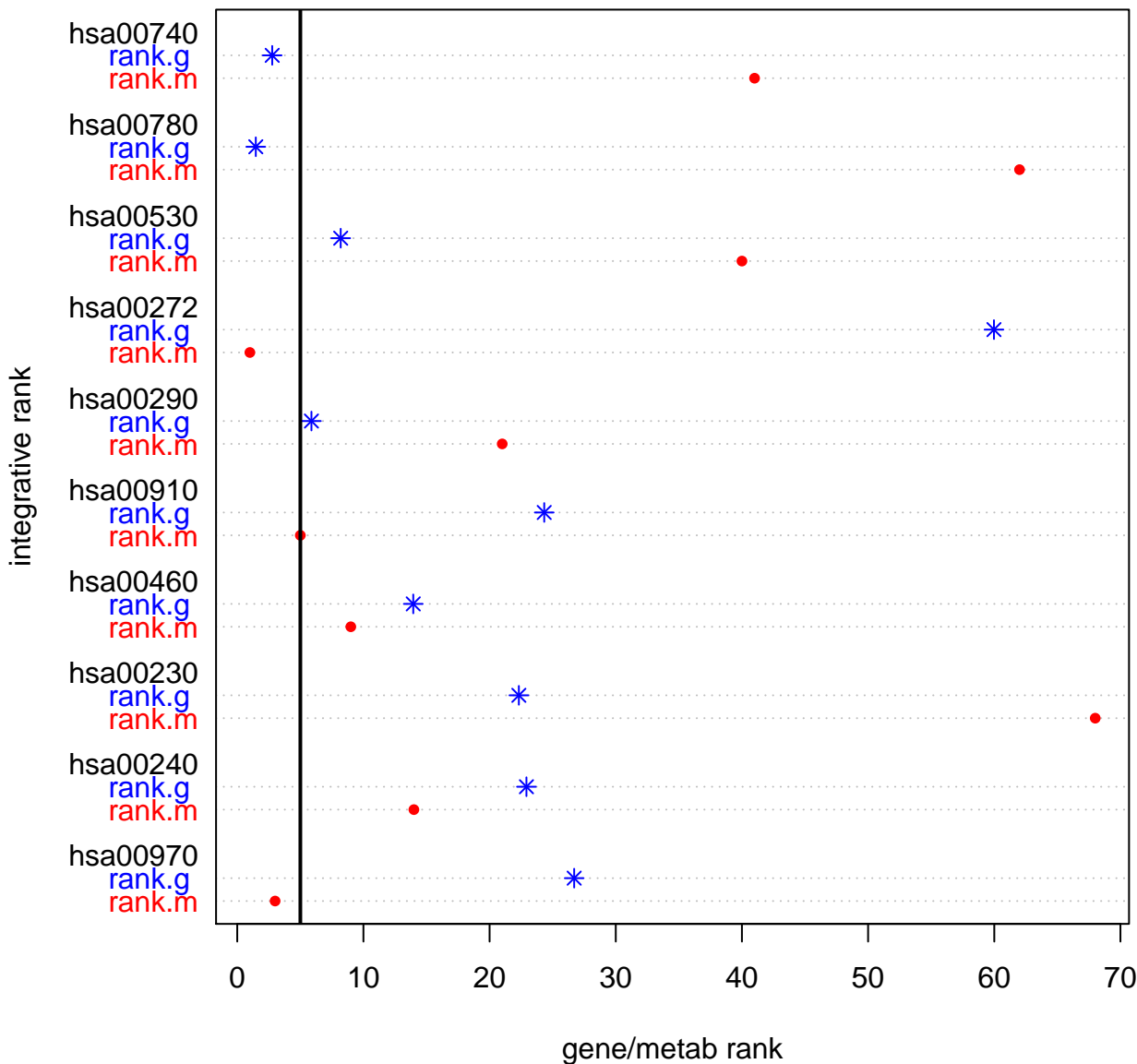

Supplement: Supplementary Software — R software code used for integrative analysis [file ncomms11612-s3.zip › integHBP/Analysis/rankcomp_top10_042115.pdf]

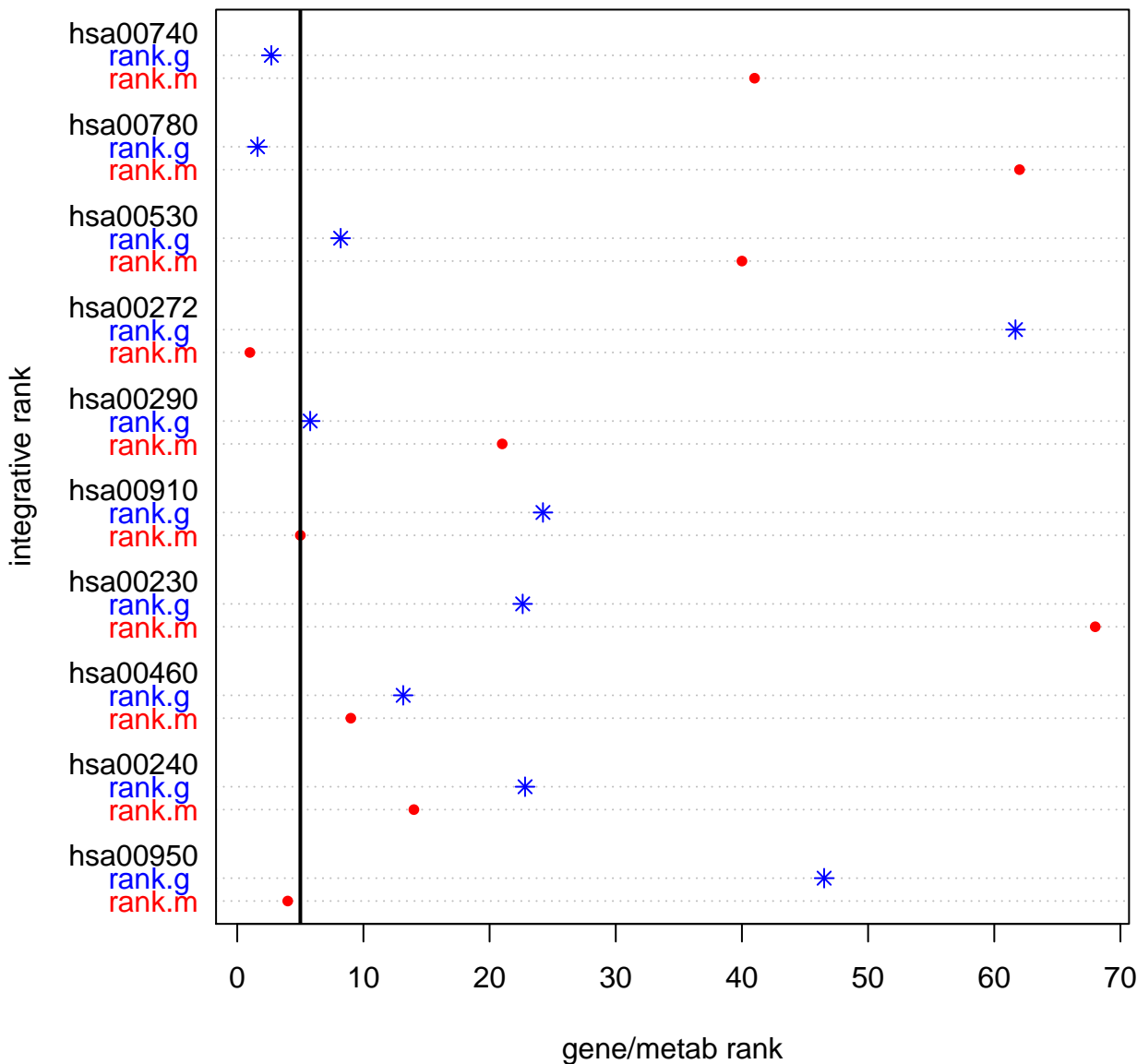

Supplement: Supplementary Software — R software code used for integrative analysis [file ncomms11612-s3.zip › integHBP/Analysis/rankcomp_top10_120515.pdf]
